# Immature stages of Palearctic *Mecinus* species (Coleoptera, Curculionidae, Curculioninae): morphological characters diagnostic at genus and species levels

**DOI:** 10.3897/zookeys.939.50612

**Published:** 2020-06-09

**Authors:** Rafał Gosik, Jiří Skuhrovec, Roberto Caldara, Ivo Toševski

**Affiliations:** 1 Department of Zoology and Nature Protection, Maria Curie-Skłodowska University, Akademicka 19, 20-033 Lublin, Poland Maria Curie-Skłodowska University Lublin Poland; 2 Group Function of Invertebrate and Plant Biodiversity in Agro-Ecosystems, Crop Research Institute, Prague 6–Ruzyně, Czech Republic Crop Research Institute Prague Czech Republic; 3 Center of Alpine Entomology, University of Milan, Via Celoria 2, 20133 Milan, Italy University of Milan Milan Italy; 4 CABI, Rue des Grillons 1, 2800 Delémont, Switzerland Institute for Plant Protection and Environment Zemun Serbia; 5 Institute for Plant Protection and Environment, Banatska 33, 11080, Zemun, Serbia CABI Delémont Switzerland

**Keywords:** biology, mature larva, Mecinini, *
Mecinus
*, morphology, pupa, taxonomy

## Abstract

The immature stages of ten *Mecinus* species are described for the first time and those of two other species are redescribed, adding important chaetotaxy characters that were missing from previous descriptions. These species belong to six of the nine assemblages of *Mecinus* species previously established according to a phylogenetic analysis. All these groupings are confirmed on the basis of several characters of mature larvae and pupae. Moreover, all the species show several characters that are useful for distinguishing them from each other, including cryptic species that previously had few differential characters. Some characters that may be useful for separating *Mecinus* from other genera in the tribe are suggested. To confirm the taxonomic identification of some larvae, the mtCOII gene was obtained and compared with sequences from identified adult specimens. The most important characters for separating the immature stages of the genera and species groups in *Mecinus* are the number of palpomeres of the labial palpi (1 or 2), the number of air tubes of the thoracic and abdominal spiracles (unicameral or bicameral), and the number of epipharyngeal setae. The species studied herein were compared with those known from other genera in the tribe Mecinini. Two keys, one to the described larvae and the other to the pupae, are provided. Detailed biological data, several of which are new, on some species are reported.

## Introduction

The genus *Mecinus* Germar, 1821 belongs to the tribe Mecinini (Curculionidae, Curculioninae) and includes approximately 50 Palearctic species (Alonso-Zarazaga et al. 2017). Adults of this tribe were recently subjected to morphological revision (Caldara and Fogato 2013) and phylogenetic analysis (Caldara et al. 2013). Based on this analysis, seven species groups and two “complexes” of species were recognised. Moreover, a phylogenetic study on the tribe Mecinini, based on morphological characters, suggests that *Mecinus* is the sister group of the remaining Mecinini like *Gymnetron* Schoenherr, 1825 and *Rhinusa* Stephens, 1829 (Caldara 2001). Preliminary molecular studies seem to confirm the systematic separation of these genera (I. Toševski, unpublished data).

All known *Mecinus* species live on angiosperms belonging to the tribes Plantagineae and Antirrhineae of the family Plantaginaceae as recently defined (Olmstead et al. 2001; Albach et al. 2005; APG 2016). The larvae develop inside the ovaries, stems, or roots of the host plants and are sometimes able to induce the formation of galls (Hoffmann 1958; Caldara 2001; Toševski et al. 2011). Several species of the genus have been the subject of detailed ecological studies (De Clerck-Floate and Harris 2002; De Clerck-Floate and Miller 2002; McClay and De Clerck-Floate 2002; Sing et al. 2005; Toševski et al. 2011) as potential biological control agents for some species of toadflax (*Linaria* spp.) that were introduced into North America and have since become invasive (Vujnovic and Wein 1997).

To date, larvae of only approximately 30 Mecinini species have been described, while descriptions of pupae have been made for 15 Mecinini species (see Skuhrovec et al. 2018 for complete references). However, there are only a few detailed descriptions of larvae and pupae that can be used for an adequate taxonomic comparison; these include immatures of three species of *Gymnetron* (Jiang and Zhang 2015), two species of *Rhinusa* (Gosik 2010; Ścibior and Łętowski 2018), five species of *Cleopomiarus* (Skuhrovec et al. 2018; Szwaj et al. 2018) and three species of *Miarus* (Skuhrovec et al. 2018). In fact, the comparison of approximately ten previously described immatures of mecinines, including two *Mecinus* species, *M. heydenii* Wencker, 1866 (Emden 1938) and *M. janthinus* Germar, 1821 (Scherf 1964), is somewhat problematic due to the absence of important details of the chaetotaxy and/or the absence of quality drawings.

Therefore, the aims of this study were to describe several larvae and pupae of *Mecinus* for the first time, to find characters that are diagnostic at genus and species levels, and finally to compare the characters on immature stages of this genus with other genera of the same tribe that might be phylogenetically informative.

## Materials and methods

### Insect collection

The material for this study was collected mainly from June to August 2017, in localities of Serbia, Macedonia, Bulgaria and France. The immature stages, i.e., L3 larvae and pupae from every studied species, were collected from their host plants and subsequently preserved in 2 ml screw-cap micro tubes (Sarstad, Germany) filled with 96% ethanol at 4–6 °C.

### Molecular analysis

In specific cases, when two species inhabit the same host plant and larval development occurs in the same host niche, the taxonomic identity of collected larvae and pupae was confirmed by molecular methods. Since it is known that the immature specimens are unavoidably damaged by these procedures, before their sequencing the specimens were compared with the others used for the morphological study in order to be sure on their conspecificity. Total DNA was extracted using the QIAGEN Dneasy Blood & Tissue Kit (Qiagen, Hilden, Germany) according to the manufacturer’s instructions. The mitochondrial cytochrome oxidase subunit II gene (mtCOII gene) was amplified using the primers TL2-J-3038 (5’-TAATATGGCAGATTAGTGCATTGGA-3’) (Emerson et al. 2000) and TK-N 3782 (5’-GAGACCATTACTTGCTTTCAGTCATCT-3’) (EVA-Harrison Laboratory, Cornell University, Ithaca, NY, USA). The polymerase chain reactions (PCRs) contained NH4 buffer (19), 5 mM MgCl2, 0.8 mM of each dNTP, 0.75 *μ*M of each primer and 0.75 U of Taq polymerase (Fermentas) in a 20 *μ*L final volume. PCR cycles were carried out in a Mastercycler EP Gradient S (Eppendorf, Germany) with the following thermal steps: 95 °C for 5 min (initial denaturation), 40 cycles at 95 °C for 1 min, 1 min at 45 °C (annealing), 72 °C for 2 min and a final extension at 72 °C for 10 min. The amplified products of the COII gene were sequenced with the forward primer only. The sequencing was performed on an ABI Prism 3700 automated sequencer using the commercial services of Macrogen Inc. (Seoul, South Korea). In addition, adult specimens of all species, whose larvae and pupae were described in this study, were identified by two of the authors (RC and IT), based on morphology. Subsequently, specimens were sequenced for the mitochondrial COII gene. The taxonomic identity of the larvae and pupae was done by comparing their sequences with the adult ones. Pairwise distances using the p-distance model were analysed using MEGA5 software (Tamura et al. 2011). The obtained sequences were deposited in the GenBank database under accession numbers MN991999–MN992012.

### Confirmation of taxonomic status using molecular tools

Molecular analysis confirmed the taxonomic identity of the larval and pupal stages of *M. labilis* and *M. pascuorum* which occur together developing in pyxidia of *Plantago lanceolata* L., and also helped to discriminate between the immature stages of *M. pirazzolii* and *M. ictericus* (Gyllenhal, 1838), which sometimes co-occur associated with *P. arenaria* Waldst. & Kit. All *Mecinus* taxa whose immature stages are described in this study were sequenced for the mtCOII gene. Sequences were edited with FinchTV v.1.4.0 (http://www.geospiza.com) and aligned with Clustal*W* integrated in the Mega5 software (Tamura et al. 2011). Aligned sequences were truncated to 655 bp from the 3' end prior to calculating the pairwise distances among the taxa. The recorded divergences among the analysed taxa ranged from 1.5 and 23.4% between *M. janthinus*-*M. janthiniformis* and *M. collaris*-*M. heydenii*, respectively (Table 1). The complete mtCOII gene showed different lengths across *Mecinus* species, ranging from a 678 bp (*M. janthinus*) group to 696 bp. in *M. pyraster*.

**Table 1.** Mitochondrial DNA cytochrome oxidase subunit II (COII) divergence based on pairwise analysis (p-distance method) among *Mecinus* species elaborated in this study. Numbers in brackets represent complete length of the COII gene.

Species	1	2	3	4	5	6	7	8	9	10	11	12
1. *M. circulatus* (696 bp)	–											
2. *M. pyraster* (696 bp)	0.145	–										
3. *M. collaris* (693 bp)	0.177	0.186	–									
4. *M. heydenii* (684 bp)	0.221	0.218	0.227	–								
5. *M. laeviceps* (684 bp)	0.218	0.223	0.214	0.114	–							
6. *M. peterharrisi* (684 bp)	0.214	0.217	0.227	0.114	0.066	–						
7. *M. janthinus* (678 bp)	0.184	0.180	0.184	0.236	0.226	0.236	–					
8. *M. janthiniformis* (678 bp)	0.178	0.178	0.180	0.236	0.233	0.233	0.018	–				
9. *M. sicardi* (678 bp)	0.181	0.168	0.181	0.211	0.208	0.212	0.103	0.100	–			
10. *M. labilis* (693 bp)	0.149	0.156	0.186	0.220	0.211	0.215	0.190	0.187	0.180	–		
11. *M. pascuorum* (693 bp)	0.184	0.187	0.173	0.208	0.204	0.205	0.201	0.193	0.180	0.183	–	
12. *M. pirazzolii* (693 bp)	0.192	0.181	0.190	0.217	0.214	0.215	0.198	0.193	0.193	0.176	0.189	–

### Morphological descriptions

Part of the larval and pupal material was preserved in Pampel fixation liquid (see Skuhrovec and Bogusch 2016) and used for the morphological descriptions. To prepare the slides, we followed May (1994): a larva was decapitated, and the head was cleared in a 10% potassium hydroxide (KOH) solution and then rinsed in distilled water. After clearing, the mouthparts were separated from the head capsule, and the head capsule and all mouthparts were mounted on permanent microscope slides in Euparal. All other body parts were mounted on temporary microscope slides in 10% glycerine.

The observations and measurements were conducted using a light microscope with calibrated ocular lenses (Olympus BX 40 and Nikon Eclipse 80i). The following characters were measured for each larva: head width, body length (larvae fixed in a C-shape were measured in segments), and body width in the widest place (i.e., metathorax or abdominal segments I–IV). For the pupae, the length and width at the widest place were measured. All results of the measurements are given in Table 2 (mature larva) and in Table 3 (pupa). The lengths of all setae are visible in the figures.

**Table 2.** Measurements (in mm) of body parts (mature larva) in studied *Mecinus* species. ^n^ = number of specimens.

*Mecinus* species	Body length	Body width	Head width
*M. pascuorum*	1.60^1^, 1.70^2^, 1.80^2^, 1.90^1^, 1.96^1^	1.00^2^, 1.10^3^, 1.20^2^	0.36^1^, 0.38^2^, 0.40^4^
*M. labilis*	1.40^1^, 1.90^1^, 2.00^1^	0.84^1^, 0.90^1^, 1.00^1^	0.36^1^, 0.38^1^, 0.40^1^
*M. pirazzolii*	1.40^2^, 1.50^4^, 1.60^2^, 1.66^4^, 1.83^2^, 2.00^2^	0.73^8^, 0.86^7^, 1.00^1^	0.36^10^, 0.40^6^
*M. circulatus*	2.33^1^, 2.50^2^, 2.66^1^, 2.73^1^	0.83^1^, 1.00^2^, 1.06^2^	0.50^1^, 0.53^4^
*M. pyraster*	2.00^1^, 2.16^1^, 2.66^1^, 2.83^1^	0.83^2^, 1.00^2^	0.50^1^, 0.53^2^, 0.56^1^
*M. collaris*	2.00^1^, 2.33^1^, 2.66^7^, 2.83^2^, 3.00^3^, 3.16^4^, 3.33^5^, 3.66^2^	0.80^3^, 0.83^8^, 1.00^10^, 1.16^4^	0.56^12^, 0.60^8^, 0.63^4^, 0.66^1^
*M. janthinus*	4.00^2^, 4.10^1^, 4.50^2^, 4.75^1^	1.00^1^, 1.10^1^, 1.25^4^	0.50^1^, 0.52^1^, 0.55^3^, 0.57^1^
*M. janthiniformis*	1.66^1^, 1.83^1^, 2.00^1^, 2.16^2^, 2.50^1^, 2.73^2^, 2.90^2^	0.66^2^, 0.73^2^, 0.83^2^, 1.00^2^, 1.10^2^	0.53^4^, 0.60^2^, 0.63^1^, 0.66^3^
*M. sicardi*	2.71^1^, 3.40^1^, 3.75^2^	1.10^2^, 115^1^, 1.25^1^	0.60^2^, 0.62^1^, 0.65^1^
*M. heydenii*	2.16^2^, 2.20^1^, 2.36^1^, 2.53^1^, 2.66^1^	0.83^1^, 0.90^2^, 0.96^1^, 1.00^2^	0.30^4^, 0.33^2^
*M. laeviceps*	1.67^1^, 1.77^1^, 1.90^1^, 2.00^1^, 2.27^2^, 2.33^1^, 2.67^1^	0.37^1^, 0.40^1^, 0.46^2^, 0.57^3^, 0.83^1^	0.30^4^, 0.33^3^, 0.40^1^
*M. peterharrisi*	2.00^3^, 2.50^4^, 2.75^5^, 3.00^4^, 3.50^6^, 3.75^2^	0.65^5^, 0.75^9^, 1.00^10^	0.35^2^, 0.36^8^, 0.38^3^, 0.40^5^, 0.42^3^, 0.43^3^

**Table 3.** Measurements (in mm) of body parts (pupa) in studied *Mecinus* species. ^n^ = number of specimens; BL = body length; BW = body width; HW = head width.

	Male			Female		
*Mecinus* species	BL	BW	HW	BL	BW	HW
*M. pascuorum*	1.52^1^, 1.70^2^, 1.72^1^, 1.74^1^, 1.90^1^, 1.96^1^	0.90^1^, 0.94^1^, 0.98^1^, 1.00^3^, 1.20^1^	0.32^3^, 0.34^2^, 0.36^1^, 0.40^1^	1.70^1^, 1.76^1^, 1.88^3^, 1.94^1^, 2.00^1^, 2.10^1^	0.94^1^, 1.00^2^, 1.06^1^, 1.10^1^, 1.12^1^, 1.16^1^, 1.20^1^	0.34^2^, 0.36^1^, 0.38^1^, 0.40^4^
*M. labilis*	1.40^2^, 1.80^1^, 2.00^1^	1.00^2^, 1.04^1^, 1.40^1^	0.36^1^, 0.38^3^	1.90^3^, 2.20^1^,	1.00^2^, 1.10^2^	0.38^3^, 0.40^1^
*M. pirazzolii*	1.63^2^, 1.70^1^, 1.73^1^, 1.80^1^	0.93^4^, 0.96^1^	0.33^4^, 0.36^1^	1.80^1^, 1.96^2^, 2.03^1^, 2.10^2^	0.96^3^, 1.03^2^, 1.16^1^	0.36^5^, 0.40^1^
*M. circulatus*	2.56^1^, 2.67^1^	1.20^1^, 1.40^1^	0.46^1^, 0.50^1^	2.50^1^, 2.53^2^, 2.60^1^, 2.66^2^, 2.73^1^, 3.00^1^	1.16^1^, 1.20^1^, 1.23^2^, 1.33^3^, 1.40^1^	0.46^6^, 0.50^2^
*M. pyraster*	3.33^1^, 340^1^	1.40^1^, 1.46^1^	0.53^2^	3.66^1^, 3.83^1^, 4.26^1^	1.66^2^, 1.73^1^	0.60^2^, 0.63^1^
*M. collaris*	1.66^1^,1.83^1^, 2.03^1^, 2.16^2^, 2.20^1^, 2.23^1^, 2.33^2^, 2.26^1^	0.76^1^, 0.96^1^, 1.00^1^, 1.03^1^, 1.06^2^, 1.13^3^, 1.20^1^	0.30^3^, 0.33^4^, 0.36^3^	1.83^2^, 2.00^4^, 2.16^3^, 2.33^2^	0.76^2^, 0.90^1^, 0.93^2^, 1.00^4^, 1.13^2^	0.33^6^, 0.36^5^
*M. janthinus*	3.25^1^, 3.60^1^, 4.00^1^	1.16^2^, 1.23^1^	0.46^1^, 0.50^2^	3.70^1^, 3.75^1^, 3.95^1^, 4.05^1^, 4.25^1^	1.16^1^, 1.20^1^, 1.36^1^, 1.40^1^, 1.50^1^	0.46^1^, 0.50^3^, 0.53^1^
*M. janthiniformis*	3.23^1^, 3.33^1^, 3.66^1^, 3.93^1^, 4.00^1^, 4.33^1^	1.20^1^, 1.33^1^, 1.42^1^,1.43^1^, 1.50^2^	0.46^1^, 0.50^1^, 0.53^1^, 0.60^3^	3.83^1^, 4.00^2^, 4.06^1^, 4.16^1^, 4.26^1^	1.26^1^, 1.43^1^, 1.50^2^, 1.66^1^, 1.80^1^	0.53^1^, 0.60^3^,0.63^1^, 0.66^1^
*M. sicardi*	3.75^1^, 4.25^2^	1.75^2^, 1.80^1^	0.60^2^, 0.65^1^	4.25^1^	2.00^1^	0.70^1^
*M. heydenii*	2.10^1^, 2.20^1^,2.33^1^, 2.60^1^ 2.66^1^	0.63^1^, 0.66^1^, 0.73^1^, 1.06^1^, 1.13^1^	0.30^2^, 0.34^1^, 0.30^1^, 0.36^1^	2.36^1^, 2.60^1^,2.66^1^, 2.73^1^, 2.93^1^	0.70^2^,0.83^1^, 1.00^1^, 1.16^1^	0.30^2^, 0.34^2^, 0.36^1^
*M. laeviceps*	2.12^1^, 2.37^1^	0.87^1^, 1.02^1^	0.35^2^	2.50^2^	1.07^2^	0.40^2^
*M. peterharrisi*	2.46^1^, 2.83^5^, 3.10^3^	0.83^2^, 1.20^6^, 1.33^1^	0.36^3^, 0.40^6^	3.00^3^, 3.23^5^, 3.66^2^	1.20^2^, 1.33^5^, 1.50^3^	0.36^4^, 0.40^3^, 0.43^3^

Drawings were created with a drawing tube on a light microscope and processed by a computer (Adobe Photoshop, Corel Photo-Paint 11, GIMP 2). The numbers of setae in bilateral structures are given for one side.

We used the terms and abbreviations for the setae of the mature larvae and pupae found in Scherf (1964), May (1977, 1994), and Marvaldi (1998, 1999).

The sequence of the species followed that proposed by Caldara and Fogato (2013) and Caldara et al. (2013).

### Botanical taxonomy

For families and subfamilies, we complied with APG (2016) whereas for the complex situation concerning the nomenclature of some common species of *Plantago*, we followed the proposals by Applequist (2006) and Dowel and Shipunov (2017).

## Results

### Morphology of immature stages

#### Genus Mecinus

Taxon classificationAnimaliaColeopteraCurculionidae

Germar, 1821

F8CBA579-0864-51AC-BF10-2571452C9C66

##### Description of the mature larva

**(L3). *Measurements*** (in mm). Body length: 1.66–4.75. Body width (metathorax or abdominal segments I–II) 0.37–1.25. Head width: 0.30–0.66.

***Body*** distinctly white to yellow. Body curved, slender, rounded in cross section. Setae on body thin, in different colouration, distinctly different in length; piliform, integument often with some asperities. Prothorax slightly smaller than meso- and metathorax. Spiracle placed between the pro- and mesothorax (see e.g., Gosik et al. 2016). Abdominal segments I–III(VI) of almost equal length, next abdominal segments decreasing gradually to the terminal parts of the body. Abdominal segment X reduced to three or four anal lobes of unequal size. Anus located terminally. Thoracic spiracles uni- or bicameral, eight abdominal spiracles unicameral, all spiracles functional, close to anterior margin of segment. Prothorax with eight to eleven *prns*; two *ps*; and one *eus.* Mesothorax with one *prs*, two or three *pds*; one or two *as*; three *ss*; one *eps*; one *ps*; and one *eus.* Chaetotaxy of metathorax almost identical to that of mesothorax. Each pedal area of thoracic segments well separated, with three to six *pda.* Abdominal segments I–VIII with one *prs*; three or four *pds*; two or three *ss*; two long *eps*; one *ps*; one *lsts*; and two *eus.* Abdominal segment IX with two to four *ds*; one or two *ps*; and two *sts.* Abdominal segment X without or with up to two minute *ts*.

***Head capsule*** yellow to pale brown, rounded or flattened laterally, endocarinal line distinct, half or more than half the length of frons. Frontal sutures extended to antennae. One or two stemmata (st), anterior stemma in the form of a pigmented spot with convex cornea. Dorsum of the epicranium with five setae; *des_1_* located in the central part of epicranium, *des_2_* lateral, *des_3_* located anteriorly on epicranium close to frontal suture, *des_4_* often medially, *des_5_* located anterolaterally. Frons with three to five *fs*, *fs_1_* sometimes absent, *fs_2_* absent except one exception; *fs_4_* and *fs_5_* subequal. Head with two *les*, one or two *ves*, and one to five *pes. Antennae* located at the end of the frontal suture on each side, membranous and distinctly convex basal article bearing three or four sensilla and a conical sensorium, the later elongated, narrow. ***Clypeus*** trapezium-shaped, with one or two *cls*, and one sensillum (*clss*); all very close to margin with frons. Labrum with three *lms*; anterior margin bisinuated; *lrs_1_* placed posteromedially, *lrs_2_* anteromedially, *lrs_3_* posterolaterally. Epipharynx with three finger-like *als*; with two or three *ams*; and one or two *mes*; labral rods (lr) distinct, kidney-shaped. Mandibles distinctly broad, bifid, teeth of unequal height; slightly truncate; both setae piliform. Maxilla stipes with one *stps*, two *pfs* and one short to minute *mbs*; mala with six or seven finger-like *dms*; four or five *vms*; all *vms* distinctly shorter than *dms.* Maxillary palpi with two palpomeres; basal palpomere with one short *mxps* and two sensilla; distal palpomere with one sensillum and a group of microcuticular apical processes. Prelabium various in shape, with one *prms*; ligula with sinuate margin and two or three *ligs*; premental sclerite well sclerotised but without anterior and posterior extensions, U-shaped or cup-like. Labial palpi with one or two palpomeres; each of the palpomeres with one sensillum, distal palpomere with cuticular apical processes. Postlabium with three *pms*: *pms_1_* usually the shortest, placed anteromedially or anterolaterally, *pms_2_* the longest, placed laterally, and *pms_3_* short or medium, placed posterolaterally.

##### Description of pupa.

***Measurements*** (in mm). Head width: 0.28–0.75. Body width: 0.90–2.15. Body length: 1.20–5.00.

***Body*** stout or elongate; normally white, but sometimes yellowish; cuticle smooth. Rostrum various in length, from two to five times as long as wide. Antennae short or elongate. Pronotum 1.1–2.2 times as wide as long. Meso- and metanotum often equal in length. Abdominal segments I–(IV)VII of equal length; segment VIII almost semicircle, segment IX distinctly reduced. Spiracles on abdominal segments placed dorsolaterally; on segments I–V functional, on segment VI atrophied on next ones invisible. Urogomphi (ur) short or elongate.

***Chaetotaxy*** often well developed, but sometimes almost invisible. Head capsule without or with one *vs*, without or with up to two *sos*, without or with up to two *os*. Rostrum without or with up to two *rs*, and without or with one *pas*. Pronotum without or with up to two *as*, without or with one *ds*, one or two *sls*, without or with up to two *ls*, and two to four *pls.* Dorsal parts of meso- and metathorax with two or three setae. Apex of femora normally with one short *fes.* Abdominal segments I–VIII without or with up to two setae laterally and without or with up to three setae ventrally. Dorsal parts of abdominal segments I–VII with three to seven setae; abdominal segment VIII with three to six setae dorsally. Abdominal segment IX without or with up to four micro-setae ventrally.

### Descriptions of immature stages of the species

#### Mecinus pascuorum

Taxon classificationAnimaliaColeopteraCurculionidae

group

0467110C-DBB5-5F9D-B8CE-18B5550FC076

##### Differential diagnosis.

**Larva.** (1) cuticle of the body tuberculate; (2) pedal lobes prominent, clearly distinct; (3) abdominal segment X reduced to three anal lobes; (4) thoracic spiracle unicameral; (5) abdominal setae various in length, progressively longer from abdominal segment I to VIII; (6) abdominal segments I–VIII with four *pds* and two *ss*; (7) head white, rounded; (8) frontal suture weakly visible; (9) endocarina 4/5 length of frons; (10) *des_4_* short; (11) presence of *fs_1_*; (12) absence of *fs_2_*; (13) *fs_3_* very short; (14) head with one stemma; (15) absence of *cls_1_*; (16) labial palpi one-segmented; (17) premental sclerite cup-like, posterior extension with short, dull apex; (18) surface of postlabium smooth.

**Pupa.** (1) body stout, rather short; (2) urogomphi short; (3) rostrum moderately slender; (4) setae various in length; (5) head with one *os*; (6) rostrum with one or two *rs*; (7) pronotum with two *as*, without or with one *ds*, two *ls*, three *pls*; (8) meso- and metanotum with two setae; (9) abdominal segments I–VII with two or three setae dorsally and three minute setae ventrally.

##### Remarks and comparative notes.

The adults of this assemblage of several taxa are mostly very similar to each other, but, lacking synapomorphies, they were treated by Caldara et al. (2013) as a “complex” of species. Overall, they are characterised by small size (length shorter than 2.5 mm), usually with short, oval elytra, and with integument, at least in part, reddish. The larvae also have a combination of characters that distinguish them from those of the other groups, although with no clear autapomorphies. In contrast, the pupae are unique in having abdominal segments I–VII with 2–3 setae dorsally and three minute setae ventrally. Therefore, we can consider these species as an informal group like the other species groups.

#### Mecinus pascuorum

Taxon classificationAnimaliaColeopteraCurculionidae

(Gyllenhal, 1813)

D339190D-D364-53C5-AB6E-2B13D177D4A1

##### Material examined.

7 L3 larvae and 13 pupae, Serbia, Staničenje, 6.07.2017, 43°12.915'N, 22°30.495'E, 364 m., ex *Plantago lanceolata*, lgt. I. Toševski. Accession numbers of sequenced specimens MN992009 (larva), MN992010 (pupa).

##### Description of mature larva

**Figure 1.**
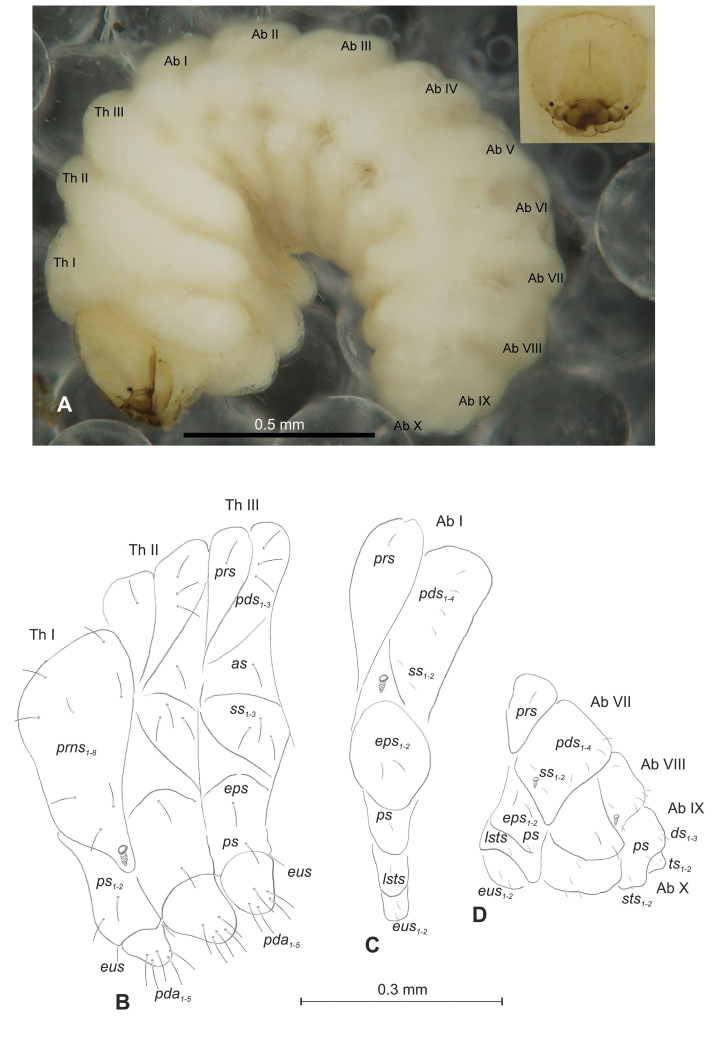
*Mecinus pascuorum* mature larva, habitus and chaetotaxy **A** habitus of the body and frontal view of the head **B** lateral view of thoracic segments **C** lateral view of abdominal segment I **D** lateral view of abdominal segments VII–X. Abbreviations: Th. I–III – number of thoracic segments, Abd. I–X – number of abominal segments, setae: *as* – alar, *ds* – dorsal, *eps* – epipleural, *eus* – eusternal, *lsts* – laterosternal, *pda* – pedal, *pds* – postdorsal, *prns* – pronotal, *prs* – prodorsal, *ps* – pleural, *ss* – spiracular, *sts* – sternal, *ts* – terminal.

**Figure 2.**
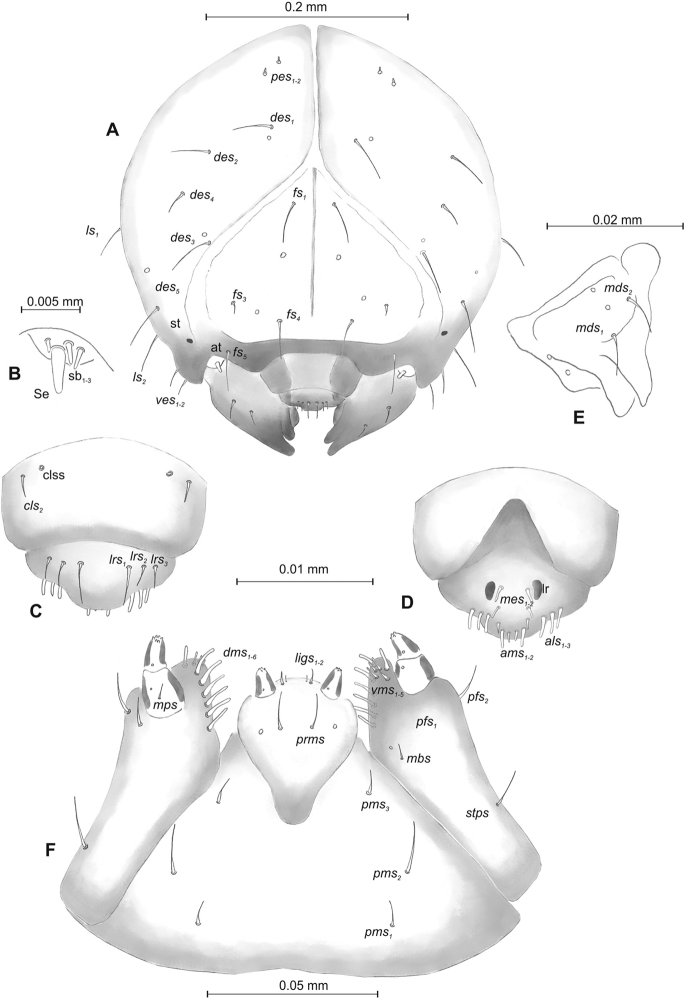
*Mecinus pascuorum* mature larva, head and mouth parts **A** head, frontal view **B** antenna **C** clypeus and labrum, dorsal view **D** epipharynx **E** left mandible **F** maxillolabial complex, ventral aspect. Abbreviations: *at* – antenna, *clss* – clypeal sensorium, *des* – dorsal epicranial, lr – labral rods, sb – sensillum basiconicum, Se – sensorium, st – stemmata, setae: *als* – anterolateral, *ams* – anteromedial, *cls* – clypeal, *dms* – dorsal malar, *fs* – frontal, *ligs* – ligular, *lrs* – labral, *ls* – lateral epicranial, *mbs* – malar basiventral, *mds* – mandibular, *mes* – median, *mxps* – maxillary palp, *pes* – postepicranial, *ves* – ventral, *pfs* – palpiferal, *pms* – postlabial, *prms* – prelabial, *stps* – stipal, *vms* – ventral malar.

(Figures 1A–D, 2A–F). ***Measurements*** (in mm). Body length: 1.60–1.96. Body width (metathorax): 1.00–1.20. Head width: 0.36–0.40.

***Body*** (Figure 1A–D) white, slender, curved. Chaetotaxy of thoracic segments relatively well developed, setae capilliform, different in length, light yellow, on thoracic segments elongated or medium, on abdominal segments very short. Prothorax (Figure 1B) with eight *prns* of almost equal length, two *ps* and one *eus.* Meso- and metathorax (Figure 1B) with one medium *prs*, three medium *pds*, equal in length; one medium *as*, three medium *ss*, equal in length; one long *eps*, one long *ps* and one long *eus.* Pedal area with five long, equal in length *pda.* Abdominal segments I–VIII (Figure 1C, D) with one very short *prs*, four short *pds* (arranged along the posterior margin), two minute *ss*, two short *eps*, one short *ps*, one short *lsts* and two short *eus.* Abdominal segment IX (Figure 1D) with three medium *ds*, all located close to the posterior margin, one short *ps* and two short *sts.* Each of anal lobes with two minute *ts*.

***Head capsule*** (Figures 1A, 2A–F) white, almost rounded. *Des_1–3, 5_* long, *des_4_* two times shorter than *des_1_*. *Fs_1_* as long as *des_1_*, *fs_4, 5_* elongated, equal in length. *Les_1_* medium, *les_2_* long; both *ves* very short, and two very short *pes* (Figure 2A). Antennae (Figure 2B) with conical, elongated sensorium (Se), four times as long as wide, and three sensilla basiconica. Clypeus (Figure 2C) trapezium-shaped, anterior margin slightly concave; *cls_2_* short; *clss* close to *cls_2_*. Labrum (Figure 2C) narrow, trapezium-shaped, anterior margin distinctly sinuate; *lrs_1_* long, *lrs_2_* and *lrs_3_* medium. Epipharynx (Figure 2D) with three finger-like *als* of almost equal length; two finger-like *ams*, different in length; two short finger-like *mes*; surface smooth; labral rods very short, rounded. Mandibles (Figure 2E) conical, rather wide, with divided apex (teeth of different lengths, curved); small protuberance in the middle of the cutting edge; with two medium *mds* capilliform, equal in length, placed transversely. Maxilla (Figure 2F) with one medium *stps* and two medium *pfs*; *mbs* short; mala with six long rod-like *dms* of almost equal size, five *vms* different in length. Maxillary palpi: basal palpomere distinctly wider and longer than distal. Prelabium (Figure 2F) cup-like with one long *prms*; ligula with two short *ligs* of different length; premental sclerite weakly developed, cup-like. Postlabium (Figure 2F) with short *pms_1_*, long *pms_2_*, and short *pms_3_*.

##### Description of pupa

**Figure 3.**
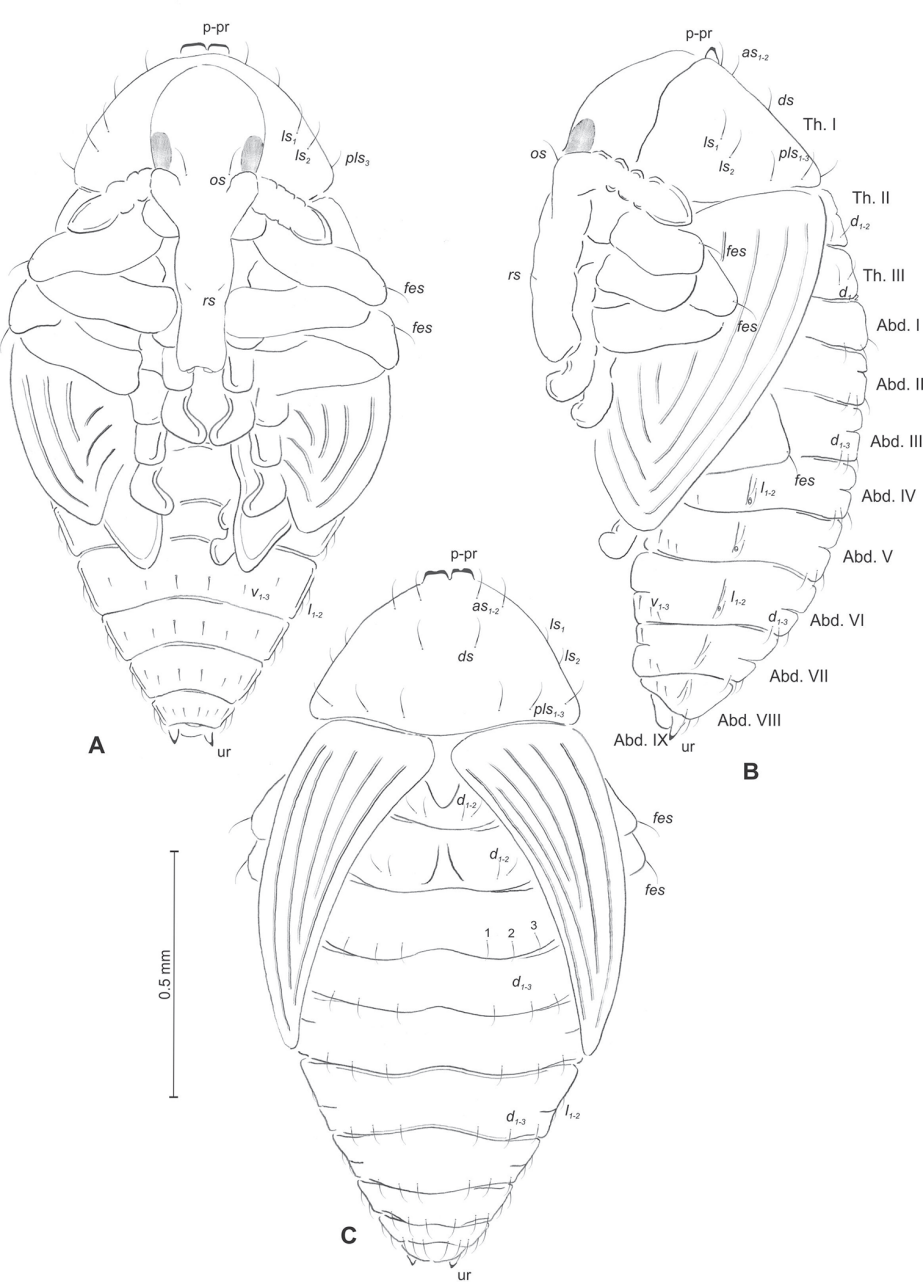
*Mecinus pascuorum* pupa habitus and chaetotaxy **A** ventral view **B** lateral view **C** dorsal view. Abbreviations: Th. I–III – number of thoracic segments, Abd. I–IX – number of abdominal segments, p–pr – pronotal protuberances, ur – urogomphi, setae: *as* – apical, *d* – dorsal, *ds* – discal, *fes* – femoral, *l*, *ls* – lateral, *os* – orbital, *pls* – posterolateral, *rs* – rostral.

(Figure 3A–C). ***Measurements*** (in mm). Head width: 0.32–0.40. Body width: 0.90–1.20. Body length: 1.52–2.10.

***Body*** moderately stout, slightly curved, white. Rostrum moderately slender, medium long, about 2.5 times as long as wide, reaching mesocoxae. Antennae rather short. Pronotum 1.7 times as wide as long, with two, conical, protuberances apically (p–pr). Urogomorpi (ur) short, conical, with sclerotised apex (Figure 3A–C).

***Chaetotaxy*** well visible, all setae (except those on rostrum and ventral part of abdomen) almost equal in length, medium. Head with one *os*. Rostrum with one minute *rs* (Figure 3A). Pronotum with two *as* placed beside protuberances, two *ls*, one *ds* and three *pls.* Dorsal parts of meso- and metathorax with two setae placed laterally. Abdominal segments I–VIII with three setae situated posteriorly, two elongated setae laterally and three short setae ventrally (median setae distinctly bigger than others). Abdominal segment IX with two micro-setae ventrally.

##### Biological notes.

This species lives on *Plantago lanceolata* L. In spring, the female lays one egg per developing pyxidium, and each larva consumes the contents of a pyxidium, usually two seeds, without causing externally visible modification. Pupation takes place within the same pyxidium. Adults emerge from the beginning of summer until September. They overwinter in the soil (Hoffmann 1958; Scherf 1964; Dickason 1968; Nieminen and Vikberg 2015).

##### Remarks and comparative notes.

This species is one of the most common species in the genus, with a very large range of distribution: Europe, the Caucasian states, the Middle East, central Asia, and Algeria (Caldara and Fogato 2013). It has been imported to North America, Australia and New Zealand (O’Brien and Wibmer 1982; Debinski and Holt 2000) and recently collected in South Africa (Caldara et al. 2009). Morphologically, this species is more closely related to other species of the group, i.e., *M. latiusculus* (Jacquelin du Val, 1855) and *M. ictericus* than to *M. labilis* studied herein, and this seems also to be corroborated by the preliminary molecular data (I. Toševski, unpublished data). However, the relationships among the immatures of these species are closer than their relationships with all the other species currently known.

#### Mecinus labilis

Taxon classificationAnimaliaColeopteraCurculionidae

(Herbst, 1795)

E1A563BB-4413-5670-8E88-41534B9FDC52

##### Material examined.

3 L3 larvae and 9 pupae, Serbia, Staničenje, 6.07.2017, 43°12.915'N 22°30.495'E, 364 m., ex *Plantago lanceolata*, lgt. I. Toševski. Accession numbers of sequenced specimen MN992008.

##### Description of mature larva

**Figure 4.**
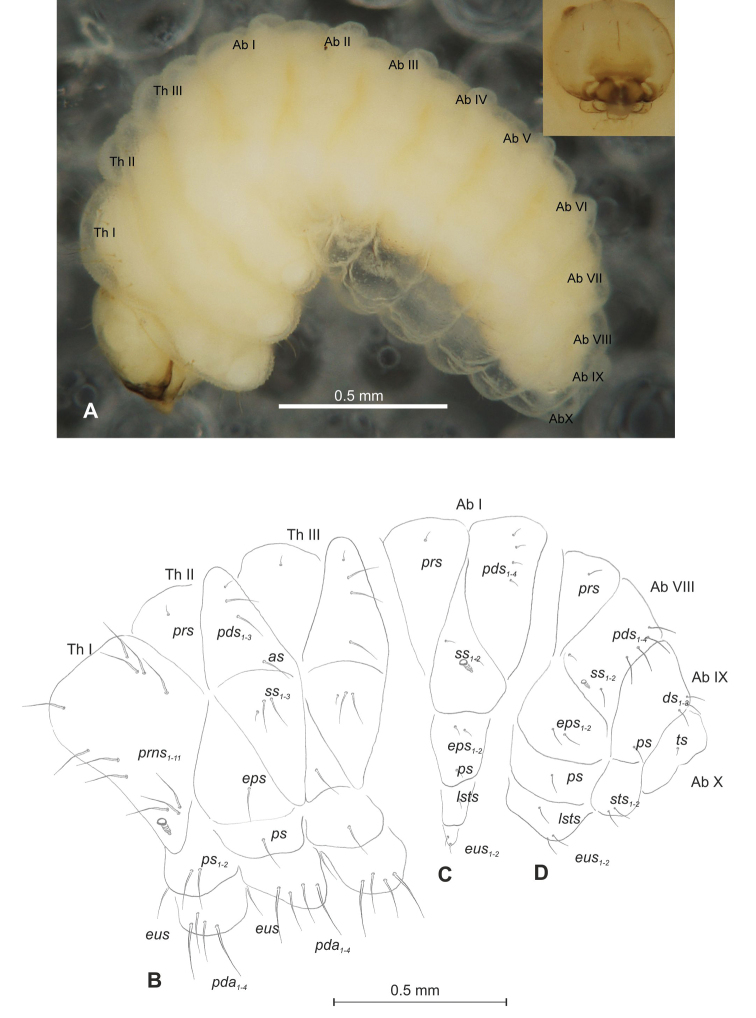
*Mecinus labilis* mature larva, habitus and chaetotaxy **A** habitus of the body and frontal view of the head **B** lateral view of thoracic segments **C** lateral view of abdominal segment I **D** lateral view of abdominal segments VII–X. Abbreviations: Th. I–III – number of thoracic segments, Abd. I–X – number of abominal segments, setae: *as* – alar, *ds* – dorsal, *eps* – epipleural, *eus* – eusternal, *lsts* – laterosternal, *pda* – pedal, *pds* – postdorsal, *prns* – pronotal, *prs* – prodorsal, *ps* – pleural, *ss* – spiracular, *sts* – sternal, *ts* – terminal.

**Figure 5.**
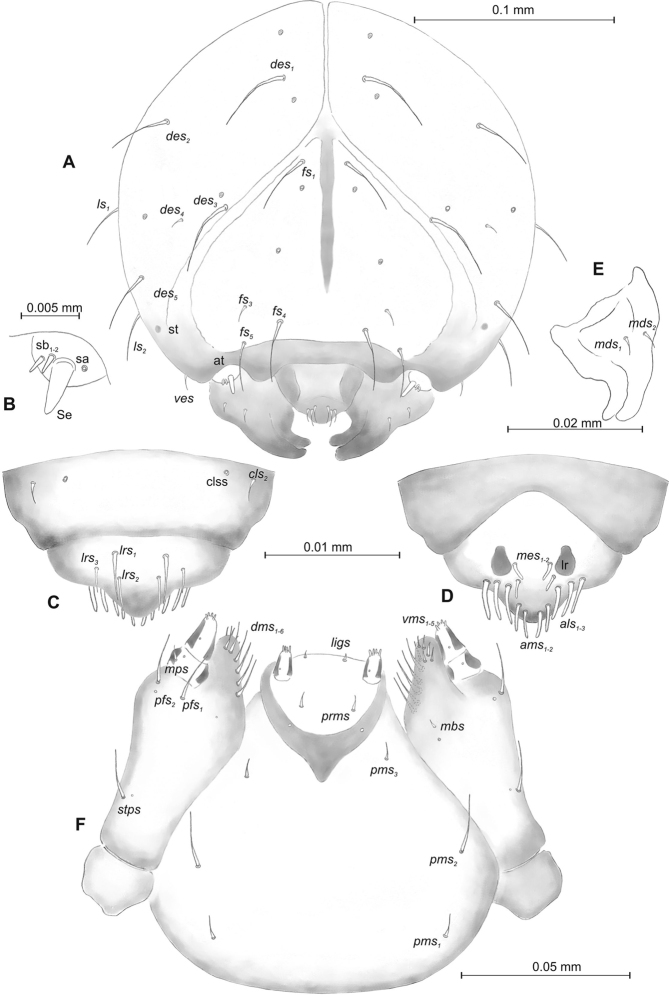
*Mecinus labilis* mature larva, head and mouth parts **A** head, frontal view **B** antenna **C** clypeus and labrum, dorsal view **D** epipharynx **E** left mandible **F** maxillolabial complex, ventral aspect. Abbreviations: *at* – antenna, *clss* – clypeal sensorium, *des* – dorsal epicranial, lr – labral rods, sa – sensillum ampullaceum, sb – sensillum basiconicum, Se – sensorium, st – stemmata, setae: *als* – anterolateral, *ams* – anteromedial, *cls* – clypeal, *dms* – dorsal malar, *fs* – frontal, *ligs* – ligular, *lrs* – labral, *ls* – lateral epicranial, *mbs* – malar basiventral, *mds* – mandibular, *mes* – median, *mxps* – maxillary palp, *pes* – postepicranial, *ves* – ventral, *pfs* – palpiferal, *pms* – postlabial, *prms* – prelabial, *stps* – stipal, *vms* – ventral malar.

(Figures 4A–D, 5A–F). ***Measurements*** (in mm). Body length: 1.40–2.00. Body width (metathorax): 0.84–1.00. Head width: 0.36–0.40.

***Body*** (Figure 4A–D) white, moderately slender, slightly curved. Chaetotaxy of thoracic segments relatively well developed, setae capilliform, different in length, light yellow, on abdominal segments I–VII very short, on segments VIII and IX of medium length. Prothorax (Figure 4B) with eleven long *prns* of almost equal length, two long *ps* and one long *eus.* Meso- and metathorax (Figure 4B) with one short *prs*, three *pds* (*pds_1_* short, *pds_2–3_* long), one long *as*, three *ss* different in length (one minute and two medium), one long *eps*, one long *ps* and one long *eus.* Pedal area with four *pda* (two long and two medium). Abdominal segments I–VIII (Figure 4C, D) with one short *prs*, four short *pds* arranged along posterior margin, two minute *ss*, two short *eps*, one short *ps*, one short *lsts* and two short *eus.* Abdominal segment IX (Figure 4D) with three medium *ds*, all located close to posterior margin, one medium *ps* and two rather short *sts.* Each of anal lobes with one minute seta.

***Head capsule*** (Figures 4A, 5A–F) white, almost rounded. *Des_1–3,5_* long, *des_4_* very short. *Fs_1_* slightly shorter than *des_1_*, *fs_4_* and *fs_5_* equal in length, almost as long as *des_1_*. *Les_1_* and *les_2_* long; one *ves* very short (Figure 5A). Antennae (Figure 5B) with elongated sensorium (Se), four times as long as wide, and two sensilla basiconica and one sensillum ampullaceum. Clypeus (Figure 5C) trapezium-shaped, anterior margin almost straight; *cls_2_* short, *clss* placed close to *cls_2_*. Labrum (Figure 5C) narrow, trapezium-shaped, anterior margin distinctly sinuate; *lrs_1_* long, *lrs_2_* and *lrs_3_* medium. Epipharynx (Figure 5D) with three elongated, finger-like *als* of equal length; two relatively elongated, finger-like *ams*; two short finger-like *mes*; surface smooth; labral rods very short, rounded. Mandibles (Figure 5E) conical, rather wide, with divided apex; both *mds* capilliform, short, equal in length, placed transversely. Maxilla (Figure 5F) with one medium *stps* and two medium *pfs*; *mbs* short; mala with six long rod-like *dms* of almost equal size, five *vms* different in length. Maxillary palpi: basal palpomere distinctly wider than distal. Prelabium (Figure 5F) cup-like with one short *prms*; ligula with one minute *lig*; premental sclerite weakly developed, cup-like. Postlabium (Figure 5F) with very short *pms_1_*, long *pms_2_*, and very short *pms_3_*.

##### Description of pupa

**Figure 6.**
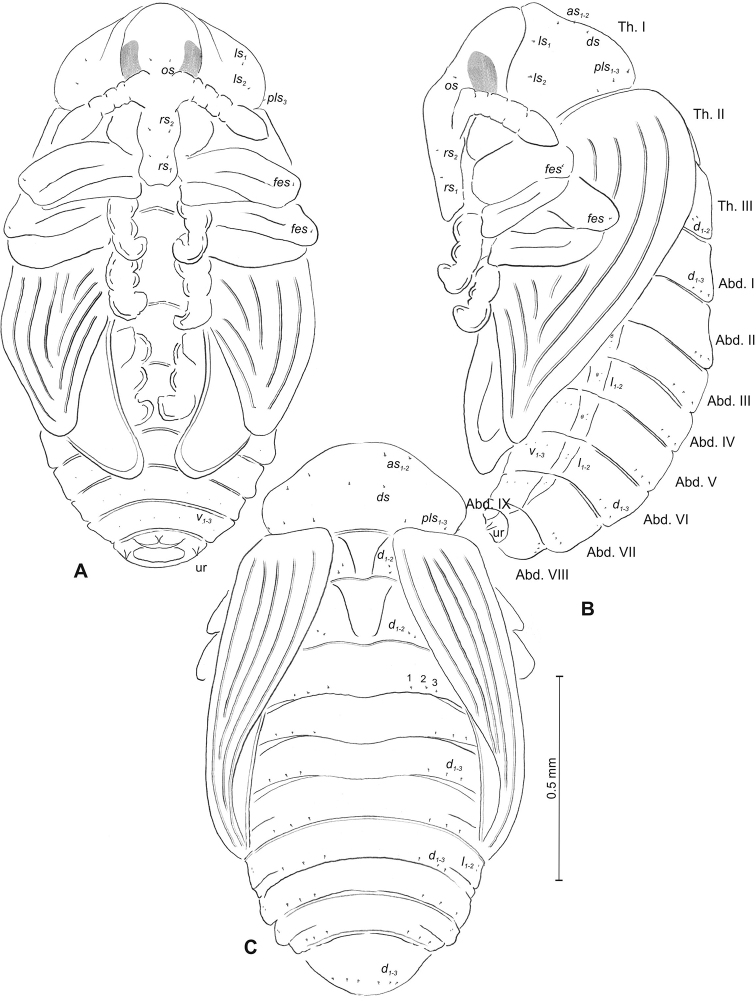
*Mecinus labilis* pupa habitus and chaetotaxy **A** ventral view **B** lateral view **C** dorsal view. Abbreviations: Th. I–III – number of thoracic segments, Abd. I–IX – number of abdominal segments, ur – urogomphi, setae: *as* – apical, *d* – dorsal, *ds* – discal, *fes* – femoral, *l*, *ls* – lateral, *os* – orbital, *pls* – posterolateral, *rs* – rostral, *sls* – superlateral.

(Figure 6A–C). ***Measurements*** (in mm). Head width: 0.36–0.40. Body width: 1.00–1.40. Body length: 1.40–2.20.

***Body*** rather stout, slightly curved, white. Rostrum slender, moderately short, about 2.0 times as long as wide, reaching procoxae. Antennae short. Pronotum 2.2 times as wide as long. Urogomorpi (ur) very short, conical, only slightly reaching outline of the body (Figure 6A–C).

***Chaetotaxy*** almost invisible, all setae minute, possible to observation only under higher magnification. Head with one *os*. Rostrum with two *rs* placed medially (Figure 6A). Pronotum with two *as*, two *ls*, one *ds* and three *pls.* Dorsal parts of meso- and metathorax with two setae placed laterally. Abdominal segments I–VIII with three setae situated posterolaterally, two setae laterally and three setae ventrally. Abdominal segment IX with two micro-setae ventrally.

##### Biological notes.

Larvae feed on *Plantago lanceolata* L. in galled pyxidia, where they pupate in the collar without causing externally visible modifications (Hoffmann 1958).

##### Remarks and comparative notes.

This species is widely distributed in Europe, the Caucasian states, and Turkey. Concerning the adults, the pattern of the elytral integument (reddish with two black oblique bands from interstria 1 to 7) and the shape of the rostrum (in lateral view moderately curved in basal half then straight to apex) allow us to separate these two species from all the others. With regard to the immatures, the differences from the other studied species of the group, *M. pascuorum*, are several and are reported in the key. Molecular data also do not show a close relationship between these two species (I. Toševski, unpublished data).

#### Mecinus simus

Taxon classificationAnimaliaColeopteraCurculionidae

group

A857E4D6-06F5-51F9-8630-1DEB2735BAD4

##### Differential diagnosis.

**Larva.** (1) cuticle of the body smooth; (2) pedal lobes prominent; (3) abdominal segment X reduced to three anal lobes of equal size; (4) thoracic spiracle unicameral; (5) all abdominal setae short or very short, without trend to become progressively longer from abd. segment I to VIII; (6) abdominal segments I–VIII with three *pds* and two *ss*; (7) head white, rounded; (8) frontal suture poorly developed; (9) endocarina 3/4 of the frons; (10) *des_4_* three times shorter than *des_1_*; (11) absence of *fs_1_*; (12) absence of *fs_2_*; (13) *fs_3_* three times shorter than *fs_4_*; (14) head with one stemma; (15) absence of *cls_1_*; (16) labial palpi one-segmented; (17) premental sclerite cup-like, posterior extension with elongated, acute apex; (18) surface of postlabium smooth.

**Pupa.** (1) body stout and short; (2) urogomphi extremely short, not reaching outline of the body; (3) rostrum short, tapering to the top; (4) setae minute, almost invisible; (5) head with one *os*; (6) rostrum with one *rs*; (7) pronotum with two *as*, one *ds*, one *ls*, three *pls*; (8) meso- and metanotum with two setae; (9) abdominal segments I–VII with three setae dorsally and without setae ventrally.

##### Remarks and comparative notes.

The very short, conical and in lateral view straight rostrum, and the protibiae with apical third distinctly enlarged, sometimes with outer margin and apex bearing stout denticles, are truly noteworthy and unique in Mecinini. Both characters are oddly similar to those of a mole, and the tibiae are similar to those of Scarabaeidae. Since nothing was known about their biology except for their host plants, Caldara and Fogato (2013) speculated on the possibility that the species of this group deposit eggs in plant roots. The new biological data on *M. pirazzolii* below reported exclude this hypothesis and suggest that most likely the female is able to approach as close as possible to the pistil of the flower and deposit the egg thanks to the shape of its protibiae, since it is regularly found deeply stuck between *Plantago* inflorescences. This group might be related to the *M. collaris* group on the basis of the morphological characters of the adults (Caldara et al. 2013), whereas it seems more related to the *M. circulatus* group according to the preliminary molecular data (I. Toševski, unpublished data). Unfortunately, the study of immatures did not clarify this situation. In fact, the presence of one palpomere on the labial palpi and of all spiracles unicameral contradicts this hypothesis, and the same combination of these two characters is found only in *M. pascuorum* and *M. heydenii* groups, with the former of which the *M. simus* group might have major similarities. However, the immatures of the *M. simus* group have some autapomorphies, such as a smooth body cuticle and prominent pedal lobes in larvae and abdominal segments I–VII with three setae dorsally and without setae ventrally, apart from an obvious extraordinarily short rostrum tapering to the apex in pupae.

#### Mecinus pirazzolii

Taxon classificationAnimaliaColeopteraCurculionidae

(Stierlin, 1867)

B5BBE85B-3B1F-56AD-9F0B-995DDC6DDC31

##### Material examined.

20 L3 larvae and 10 pupae, Serbia, Veliko Gradište, 14.07.2017, 44°45.039'N, 21°31.426'E, 86 m., *ex Plantago arenaria*, lgt. I. Toševski. Accession numbers of sequenced specimens MN992011 (larva), MN992012 (pupa).

##### Description of mature larva

**Figure 7.**
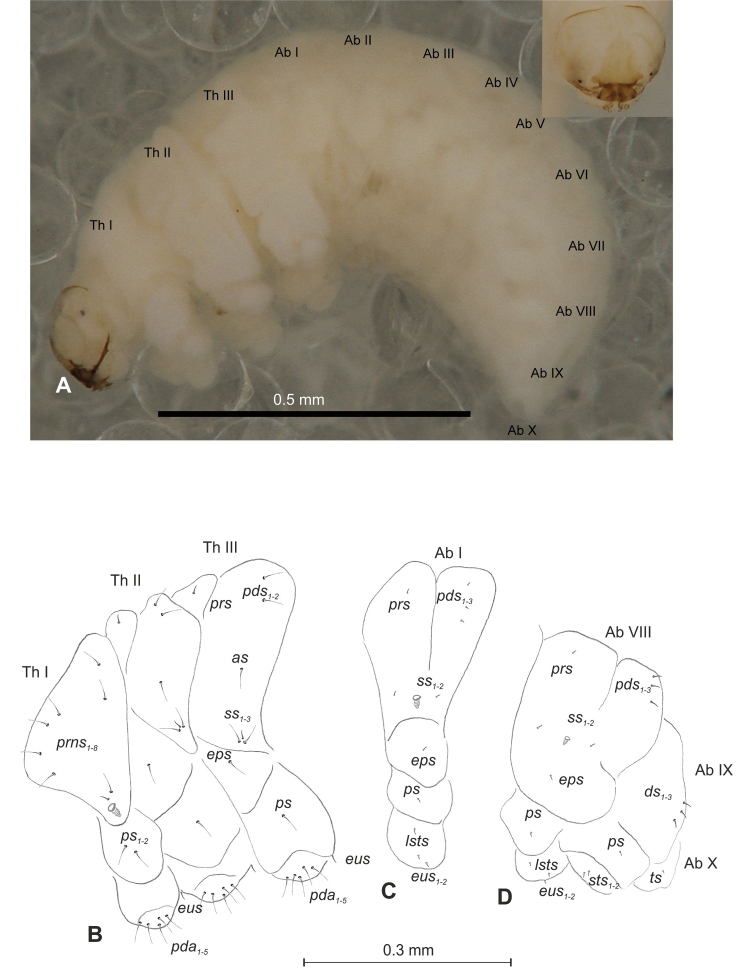
*Mecinus pirazzolii* mature larva, habitus and chaetotaxy **A** habitus of the body and frontal view of the head **B** lateral view of thoracic segments **C** lateral view of abdominal segment I **D** lateral view of abdominal segments VII–X. Abbreviations: Th. I–III – number of thoracic segments, Abd. I–X – number of abominal segments, setae: *as* – alar, *ds* – dorsal, *eps* – epipleural, *eus* – eusternal, *lsts* – laterosternal, *pda* – pedal, *pds* – postdorsal, *prns* – pronotal, *prs* – prodorsal, *ps* – pleural, *ss* – spiracular, *sts* – sternal, *ts* – terminal.

**Figure 8.**
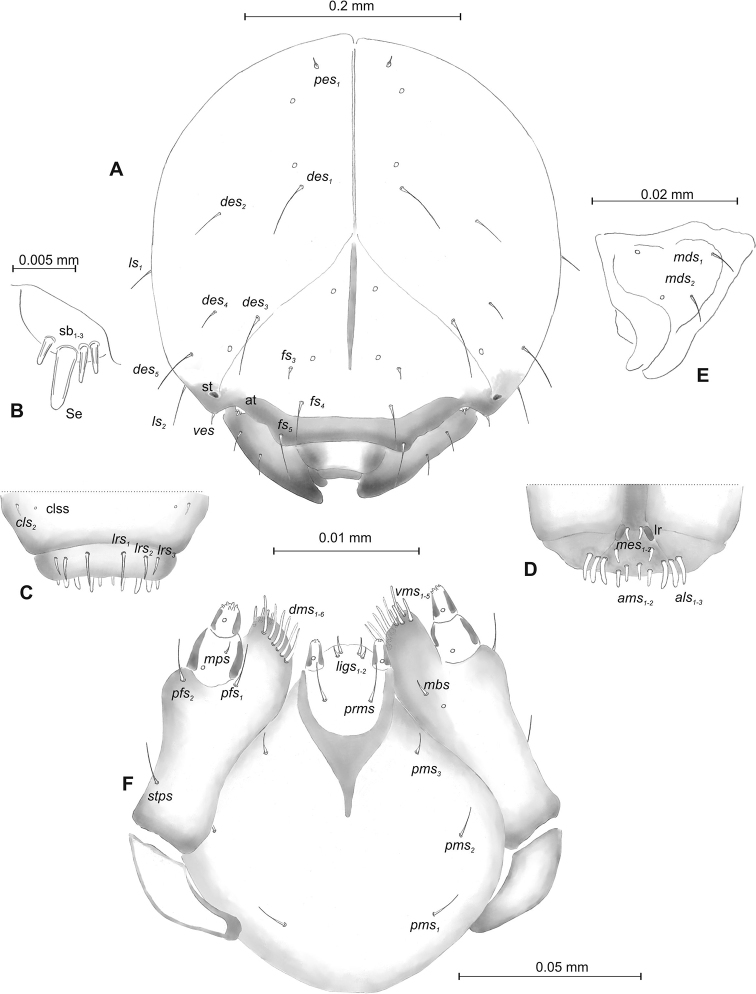
*Mecinus pirazzolii* mature larva, head and mouth parts **A** head, frontal view **B** antenna **C** clypeus and labrum, dorsal view **D** epipharynx **E** left mandible **F** maxillolabial complex, ventral aspect. Abbreviations: *at* – antenna, *des* – dorsal epicranial, lr – labral rods, sb – sensillum basiconicum, Se – sensorium, st – stemmata, setae: *als* – anterolateral, *ams* – anteromedial, *cls* – clypeal, *dms* – dorsal malar, *fs* – frontal, *ligs* – ligular, *lrs* – labral, *ls* – lateral epicranial, *mbs* – malar basiventral, *mds* – mandibular, *mes* – median, *mxps* – maxillary palp, *pes* – postepicranial, *ves* – ventral, *pfs* – palpiferal, *pms* – postlabial, *prms* – prelabial, *stps* – stipal, *vms* – ventral malar.

(Figures 7A–D, 8A–F). ***Measurements*** (in mm). Body length: 1.40–2.00. Body width (metathorax): 0.73–1.00. Head width: 0.36–0.40.

***Body*** (Figure 7A–D) white, slender, curved. Chaetotaxy of thoracic segments relatively well developed, setae capilliform, different in length, light yellow; on abdominal segments almost invisible (except dorsal parts of abdominal segments IX and X). Prothorax (Figure 7B) with eight *prns* of unequal length (seven relatively long and one medium), two relatively long *ps* and one short *eus.* Meso- and metathorax (Figure 7B) with one medium *prs*, two long *pds*, equal in length, one long *as*, three *ss* different in length (two relatively long, one short), one long *eps*, one long *ps* and one short *eus.* Pedal area with five long *pda*, equal in length. Abdominal segments I–VIII (Figure 7C, D) with one very short *prs*, three short *pds* (on segment VIII medium, equal in length), arranged along posterior margin, two minute*ss*, one short *eps*, one short *ps*, one short *lsts* and two short *eus.* Abdominal segment IX (Figure 7D) with three medium *ds*, all located close to posterior margin, one short *ps* and two short *sts.* Each of anal lobes with one minute seta.

***Head capsule*** (Figures 7A, 8A–C) light white, almost rounded. *Des_1,3,5_* long; *des_2_* two times shorter than *des_1_*; *des_4_* three times shorter than *des_1_*. *Fs_4,5_* equal in length, almost as long as *des_1_*. *Les_1_* medium, *les_2_* long; one very short *ves*, and one *pes* (Figure 8A). Antennae (Figure 8B) with conical, elongated sensorium (Se), four times as long as wide, and three sensilla basiconica. Clypeus (Figure 8C) trapezium-shaped, anterior margin slightly concave; *cls_2_* short; *clss* situated close to *cls_2_*. Labrum (Figure 8C) narrow, trapezium-shaped, anterior margin slightly sinuate; *lrs_1_* long, *lrs_2_* medium *lrs_3_* short. Epipharynx (Figure 8D) with three finger-like *als* of almost equal length; two medium finger-like *ams*; two short finger-like *mes*; surface smooth; labral rods very short, rounded. Mandibles (Figure 8E) conical, rather wide, with a small protuberance in the middle of the cutting edge; both *mds* capilliform, medium, equal in length, placed transversely. Maxilla (Figure 8F) with one *stps* and two *pfs* of equal length; *mbs* short; mala with six long rod-like *dms* of almost equal size, five *vms* different in length. Maxillary palpi: basal palpomere distinctly wider and longer than distal. Prelabium (Figure 8F) cup-like with one long *prms*; ligula with two short *ligs*; premental sclerite clearly visible, cup-shaped, posterior extension with acute apex. Postlabium (Figure 8F) with medium *pms_1_*, medium *pms_2_*, and short *pms_3_*.

##### Description of pupa

**Figure 9.**
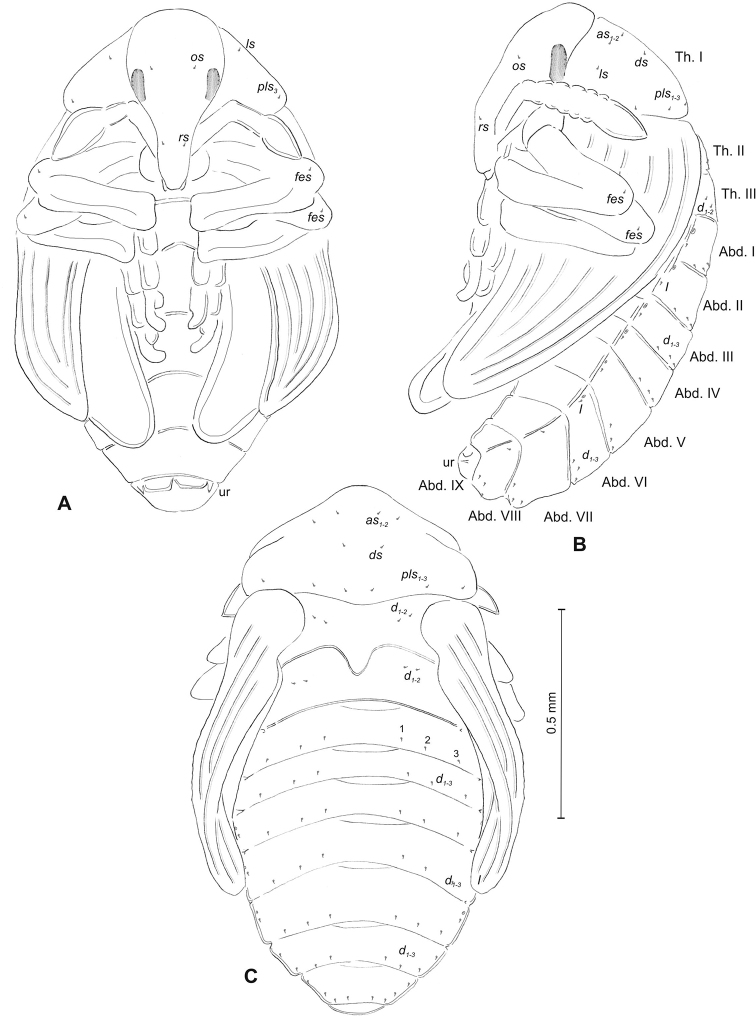
*Mecinus pirazzolii* pupa habitus and chaetotaxy **A** ventral view **B** lateral view **C** dorsal view. Abbreviations: Th. I–III – number of thoracic segments, Abd. I–IX – number of abdominal segments, ur – urogomphi, setae: *as* – apical, *d* – dorsal, *ds* – discal, *fes* – femoral, *l*, *ls* – lateral, *os* – orbital, *pls* – posterolateral, *rs* – rostral.

(Figure 9A–C). ***Measurements*** (in mm). Head width: 0.33–0.40. Body width: 0.93–1.16. Body length: 1.63–2.10.

***Body*** stout, slightly curved, white. Rostrum slender, very short, tapering to its top. Antennae moderately elongated. Pronotum 2.0 times as wide as long. Urogomorpi (ur) very short, conical, not reaching outline of the body (Figure 9A–C).

***Chaetotaxy*** almost invisible, all setae minute, possible to observation only under higher magnification. Head with one *os*. Rostrum with one *rs* placed medially (Figure 9B). Pronotum with two *as*, one *ls*, one *ds* and three *pls.* Dorsal parts of meso- and metathorax with two setae placed laterally. Dorsal parts of abdominal segments I–VIII with three setae situated posterolaterally and one seta laterally. Abdominal segment IX without setae.

##### Biological notes.

This species is associated with the annual plant *Plantago arenaria* Waldst. & Kit. The adult aggregation on plants is followed by the appearance of flowering stems with spikes in late spring. The females lay one egg onto the base of the pistil or the initialised seed. The act of oviposition is followed by proliferative growth of the ovarian tissue in the form of gall but without changes in the external morphology of the pyxidium. During development, the larvae consume all the tissue inside the pyxidium, leaving only the fruit shell intact. The larvae pupate inside the fruit shell, from which adults emerge after being completely sclerotised. Overwintering takes place in the soil litter near the host plant (I. Toševski, pers. obs.). Sympatric occurrence with *M. ictericus* is common (Caldara and Fogato 2013).

##### Remarks and comparative notes.

This species is distributed in eastern Central Europe, southeastern Europe and Turkey. In our keys, this species is closer to the species of the *M. pascuorum* group than to others, as already discussed in the Remarks for the group.

#### Mecinus circulatus

Taxon classificationAnimaliaColeopteraCurculionidae

group

72903B88-3BD6-59A7-B3DD-F001E9AA14D7

##### Differential diagnosis.

**Larva.** (1) body covered with asperities; (2) pedal lobes prominent well isolated; (3) abdominal segment X reduced to three anal lobes of unequal size; (4) thoracic spiracle bicameral; (5) abdominal setae very short, slightly growing from abdominal segment I to VIII; (6) abdominal segments I–VIII with three *pds* and two *ss*; (7) head brown, distinctly flattened laterally; (8) frontal suture poorly or well visible; (9) endocarina 1/2 of the frons; (10) *des_4_* very short or short; (11) presence of *fs_1_*; (12) absence of *fs_2_*; (13) *fs_3_* very short; (14) head with two stemmata; (15) presence of *cls_1_*; (16) labial palpi one-segmented; (17) premental sclerite cup-like; (18) surface of postlabium smooth.

**Pupa.** (1) body elongated or very elongated; (2) urogomphi slender, short or medium, reaching outline of the body, directed downward; (3) rostrum moderately elongated; (4) setae minute or medium; (5) head with one *vs*, one or two *sos*, one or two *os*; (6) rostrum with one *sls* and one *rs*; (7) pronotum with one or two *as*, without or with one *ds*, two *sls*, without or up to two *ls*, two or three *pls*; (8) meso- and metanotum with two setae; (9) abdominal segments I–VII with three or five setae dorsally.

##### Remarks and comparative notes.

The adults of this group are characterised by body elongate, subcylindrical, elytral integument reddish and black to completely black, protibiae with apical part of ventral surface distinctly directed outward. On the basis of these characters, this group might be related to *M. collaris* and especially to the *M. simus* group (Caldara et al. 2013). The study of the immatures does not support this latter relationship. The immatures of this group lack autapomorphies. However, the larvae possess the unique combination of one palpomere + thoracic spiracle bicameral and abdominal spiracles unicameral, which do not share with the species of the *M. simus* group, *M. pirazzolii*, that we have studied.

#### Mecinus circulatus

Taxon classificationAnimaliaColeopteraCurculionidae

(Marsham, 1802)

53E045BB-A67A-575D-8F27-1FFF21F24D36

##### Material examined.

5 L3 larvae and 10 pupae, 1.07.2017, Zemun, Serbia, GPS 44°39.030'N, 21°28.355'E, 162 m., lgt. I. Toševski. Accession number of sequenced specimen MN991999.

##### Description of mature larva

**Figure 10.**
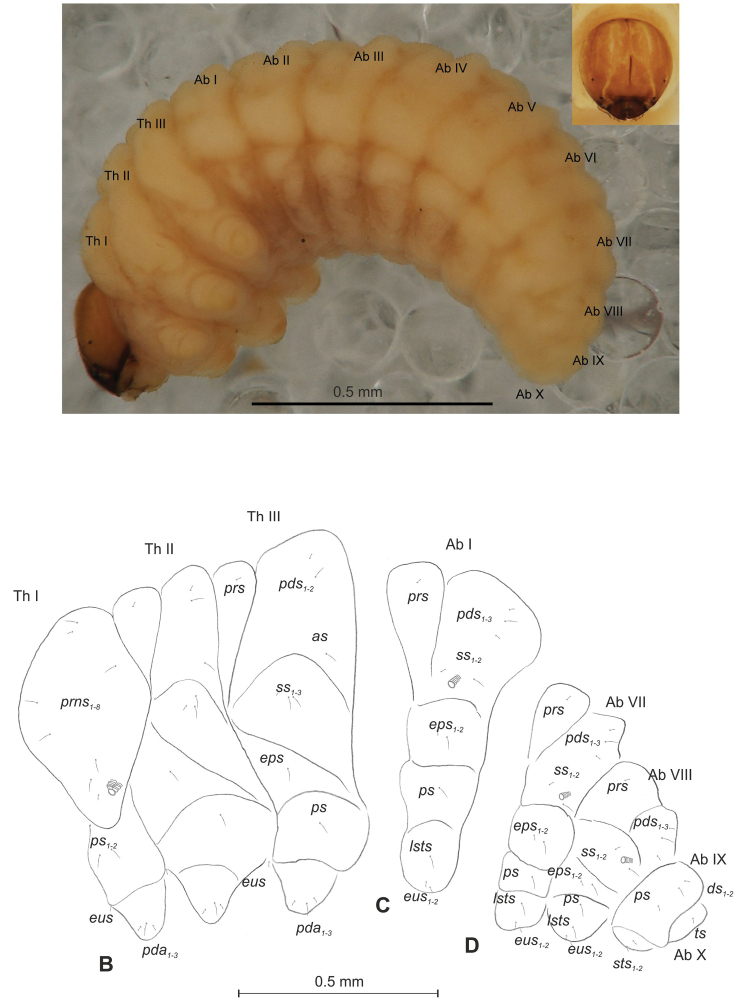
*Mecinus circulatus* mature larva, habitus and chaetotaxy **A** habitus of the body and frontal view of the head **B** lateral view of thoracic segments **C** lateral view of abdominal segment I **D** lateral view of abdominal segments VII–X. Abbreviations: Th. I–III – number of thoracic segments, Abd. I–X – number of abominal segments, setae: *as* – alar, *ds* – dorsal, *eps* – epipleural, *eus* – eusternal, *lsts* – laterosternal, *pda* – pedal, *pds* – postdorsal, *prns* – pronotal, *prs* – prodorsal, *ps* – pleural, *ss* – spiracular, *sts* – sternal, *ts* – terminal.

**Figure 11.**
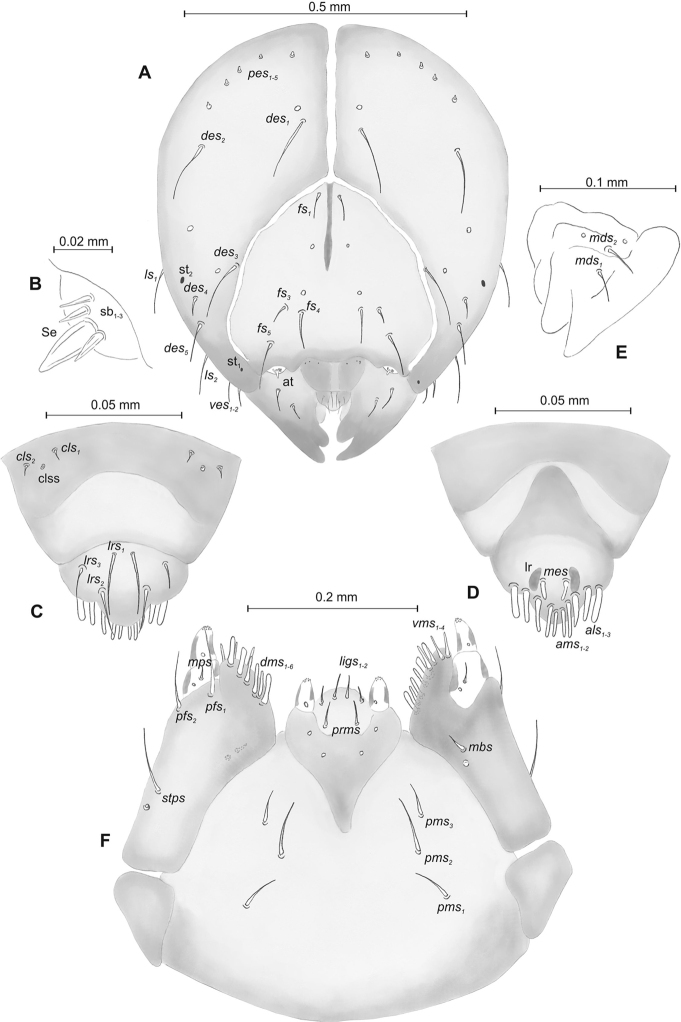
*Mecinus circulatus* mature larva, head and mouth parts **A** head, frontal view **B** antenna **C** clypeus and labrum, dorsal view **D** epipharynx **E** left mandible **F** maxillolabial complex, ventral aspect. Abbreviations: *at* – antenna, *clss* – clypeal sensorium, lr – labral rods, sb – sensillum basiconicum, Se – sensorium, st – stemmata, setae: *als* – anterolateral, *ams* – anteromedial, *cls* – clypeal, *des* – dorsal epicranial, *dms* – dorsal malar, *fs* – frontal, *ligs* – ligular, *lrs* – labral, *ls* – lateral epicranial, *mbs* – malar basiventral, *mds* – mandibular, *mes* – median, *mxps* – maxillary palp, *pes* – postepicranial, *ves* – ventral, *pfs* – palpiferal, *pms* – postlabial, *prms* – prelabial, *stps* – stipal, *vms* – ventral malar.

(Figures 10A–D, 11A–F). ***Measurements*** (in mm). Body length: 2.33–2.73. Body width (metathorax): 0.83–1.06. Head width: 0.50–0.53.

***Body*** (Figure 10A–D), light yellow, slender, curved (Figure 10B). Thoracic segments larger than abdominal segment I. Abdominal segments I–VI of almost equal length; segments VII–IX decreasing gradually to the terminal body part; segment X reduced to three anal lobes of those lateral are the largest, and dorsal the smallest (sometimes absent). Chaetotaxy weakly developed, setae short, transparent, difficult to observe (Figure 10B). Prothorax (Figure 10B) with eight *prns* (six medium and two very short); two medium *ps* and one very short *eus.* Meso- and metathorax (Figure 10B) with one very short *prs*, two *pds* (one very short, one medium), one medium *as*, three *ss* (two medium and one very short), one medium *eps*, one medium *ps* and one very short *eus.* Pedal area with three *pda* (one medium and two very short). Abdominal segments I–VIII (Figure 10C, D) with one very short *prs*, three *pds* arranged along the posterior margin (order: very short, medium and very short), two *ss* different in length, two *eps* different in length, one medium *ps*, one medium *lsts* and two very short *eus.* Abdominal segment IX (Figure 10D) with two *ds* (one medium and one very short), all located close to the posterior margin, one very short *ps* and two very short *sts.* Each lateral anal lobe (abd. seg. X) with one minute seta.

***Head capsule*** (Figures 10A, 11A–F) pale brown, narrowed bilaterally. Frontal suture poorly visible. *Des_1–3_* very long, equal in length, *des_4_* four times shorter than *des_1_*, *des_5_* slightly shorter than *des_1_*. *Fs_1_* short; *fs_3_* short, *fs_4,5_* long. *Les_1_* and *les_2_* equal in length, slightly shorter than *des_1_*; both *ves* short, and five short *pes* (Figure 11A). Antennae (Figure 11B) with conical sensorium (Se) four times as long as wide, and three sensilla basiconica. Clypeus (Figure 11C) trapezium-shaped, anterior margin concave; *cls_1-2_* relatively short; *clss* placed close to *cls_2_*. Labrum (Figure 11C) with distinctly sinuate anterior margin; *lrs_1_* very long, *lrs_2_* slightly shorter than *lrs_1_*, *lrs_3_* two times shorter than *lrs_1_*. Epipharynx (Figure 11D) with three medium, finger-shaped *als* of almost equal length; two rod-like *ams*, equal in length; one finger-like *mes* of medium length; surface smooth; labral rods close to kidney-shaped. Mandibles (Figure 11E) conical, wide, with a small protuberance in the middle of the cutting edge; both *mds* capilliform, medium, equal in length, placed mediolaterally. Maxilla (Figure 11F) with one *stps* and two *pfs* equal length; *mbs* very short; mala with six finger-like *dms* of almost equal size; four *vms* different in length. Maxillary palpi: basal palpomere distinctly wider and slightly longer than distal. Prelabium (Figure 11F) cup-like with one relatively short *prms*; ligula with two *ligs* different in length; premental sclerite well visible, cup-shaped. Postlabium (Figure 11F) with medium *pms_1_*, long *pms_2_*, and medium *pms_3_*.

##### Description of pupa.

**Figure 12.**
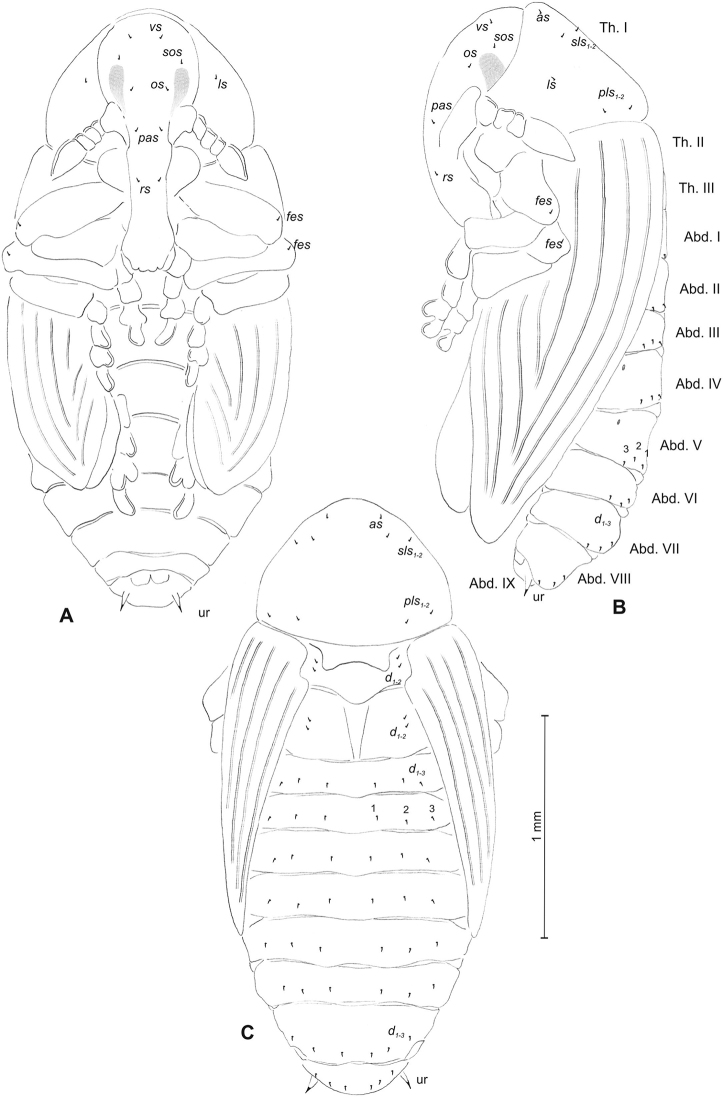
*Mecinus circulatus* pupa habitus and chaetotaxy **A** ventral view **B** lateral view **C** dorsal view. Abbreviations: Th. I–III – number of thoracic segments, Abd. I–IX – number of abdominal segments, ur – urogomphi, setae: *as* – apical, *d* – dorsal, *fes* – femoral, *l*, *ls* – lateral, *os* – orbital, *sls* – postantennal, *pls* – posterolateral, *rs* – rostral, *sls* – superlateral, *sos* – superorbital, *vs* – vertical.

(Figure 12A–C). ***Measurements*** (in mm). Head width: 0.46–0.50. Body width: 1.16–1.40. Body length: 2.46–3.00.

***Body*** moderately elongated, white. Rostrum rather short, about 3.2 times as long as wide, reaching up to mesocoxae. Antennae slender and elongated. Pronotum 1.25 times as wide as long. Mesonotum slightly shorter than metanotum. Urogomphi (ur) short, slender, conical, with sclerotised, sharp apex, slightly reaching outline of the body, directed downward (Figure 12A–C).

***Chaetotaxy*** very sparse, setae short or minute. Head with one *vs*, one *os* and one *sos.* Rostrum with one *rs* and one *pas*. Setae on head and rostrum straight, as long as those on prothorax (Figure 12A). Pronotum with one *as*, one *ls*, two *sls*, and two *pls.* Dorsal parts of meso- and metathorax with two setae placed medially. Apex of femora with one minute *fes* (Figure 12A–C). Dorsal parts of each abdominal segments I–VIII with three setae placed posteromedially along margins of each segments. Abdominal segment IX with two micro-setae ventrally.

##### Biological notes.

This species is very common on *Plantago lanceolata* L., while in southeastern Europe, it is also common on some other closely related species, such as *P. arenaria* (sub *P. psyllium* L.), *P. afra* L. (sub *P. cynops* L.) and *P. subulata* L. (Hoffmann 1958; Sprick 2001). The females oviposit in early spring on young growing vegetative shoot buds. Newly hatched larvae bore through the central part of the shoot bud, forming a 1–2 cm long larval channel that rarely rises above the root crown. The larvae pupate inside the larval channel, and the emerged adult leaves the pupa chamber after a short time. The adult overwinters in the soil litter near the host plant.

##### Remarks and comparative notes.

This is a common species in western, central and southern Europe, northern Africa and the Middle East. By the colour of the elytral integument, with black and reddish vittae, the adults differ from *M. pyraster*, whose integument is completely black. However, both the study of the morphological characters in adults and immatures and the preliminary molecular study (I. Toševski, unpublished data) agree with the hypothesis of close relationships between these two species.

Larvae are easily separable from those of *M. pyraster*: the pronotum has eight *prns* instead of 11, the pedal lobes has three *pda* instead of five, the anal lobes with one *ts* instead of two, the head with five *pes* instead of four, the mandible with two *mds* instead of one, the mala with four *vms* instead of five, and the *prms* are shorter.

Pupae differ from those of *M. pyraster* by the head with one *sos* and one *os* instead of two, the pronotum with a different number of setae in all positions, and the abdominal segments I–VII with three setae dorsally instead of five.

#### Mecinus pyraster

Taxon classificationAnimaliaColeopteraCurculionidae

(Herbst, 1795)

3FE84ECC-2C8E-5C66-BCCF-1390DAA037A7

##### Material examined.

4 L3 larvae and 5 pupae, Serbia, Zemun, 1.07.2017, GPS 44°39.030'N, 21°28.355'E, 162 m., ex l., ex *Plantago lanceolata*, lgt. I. Toševski. Accession number of sequenced specimen MN992000.

##### Description of mature larva

**Figure 13.**
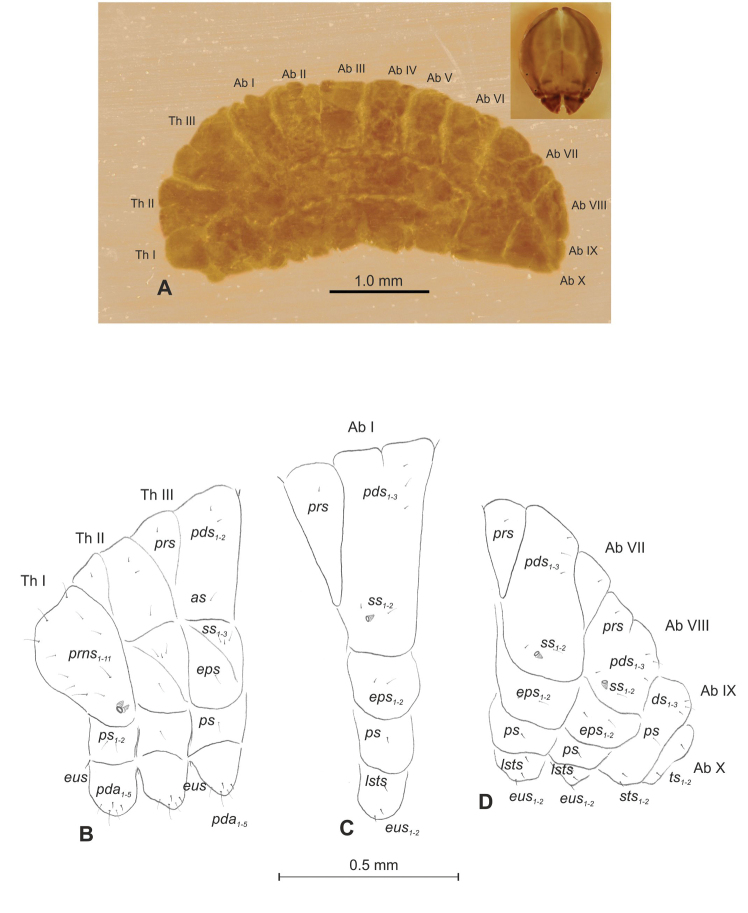
*Mecinus pyraster* mature larva, habitus and chaetotaxy **A** habitus of the body and frontal view of the head **B** lateral view of thoracic segments **C** lateral view of abdominal segment I **D** lateral view of abdominal segments VII–X. Abbreviations: Th. I–III – number of thoracic segments, Abd. I–X – number of abominal segments, setae: *as* – alar, *ds* – dorsal, *eps* – epipleural, *eus* – eusternal, *lsts* – laterosternal, *pda* – pedal, *pds* – postdorsal, *prns* – pronotal, *prs* – prodorsal, *ps* – pleural, *ss* – spiracular, *sts* – sternal, *ts* – terminal.

**Figure 14.**
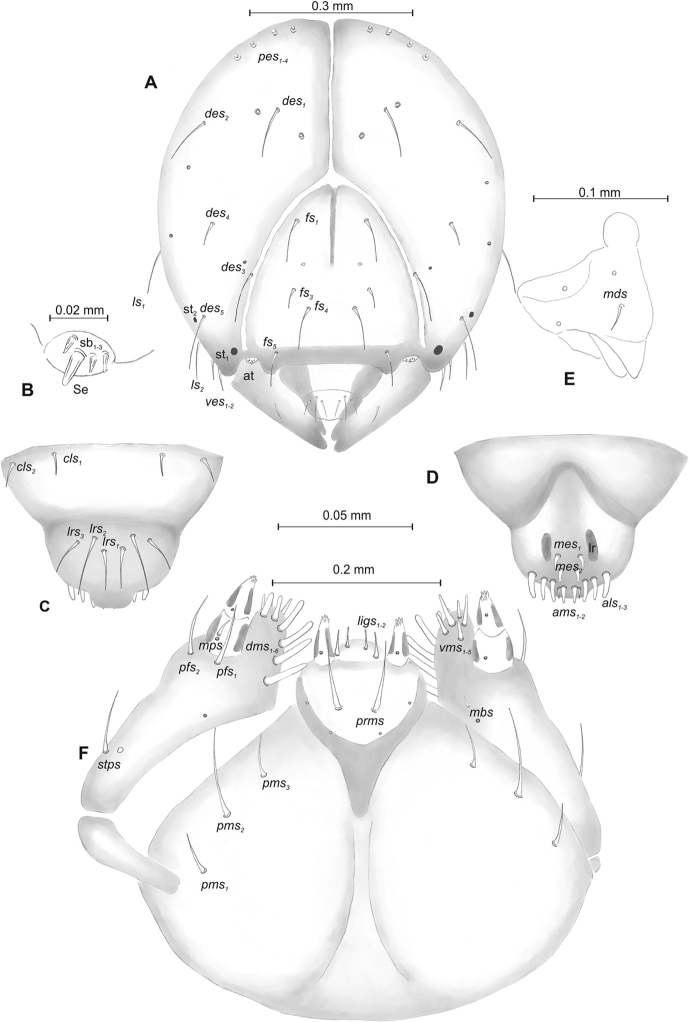
*Mecinus pyraster* mature larva, head and mouth parts **A** head, frontal view **B** antenna **C** clypeus and labrum, dorsal view **D** epipharynx **E** left mandible **F** maxillolabial complex, ventral aspect. Abbreviations: *at* – antenna, lr – labral rods, sb – sensillum basiconicum, Se – sensorium, st – stemmata, setae: *als* – anterolateral, *ams* – anteromedial, *cls*– clypeal, *des* – dorsal epicranial, *dms* – dorsal malar, *fs* – frontal, *ligs* – ligular, *lrs* – labral, *ls* – lateral epicranial, *mbs* – malar basiventral, *mds* – mandibular, *mes* – median, *mxps* – maxillary palp, *pes* – postepicranial, *ves* – ventral, *pfs* – palpiferal, *pms* – postlabial, *prms* – prelabial, *stps* – stipal, *vms* – ventral malar.

(Figures 13A–D, 14A–F). ***Measurements*** (in mm). Body length: 2.00–2.83. Body width (metathorax or abdominal segments I–II): 0.83–1.00. Head width: 0.53–0.56.

***Body*** (Figure 13A–D) yellowish, slender, curved, densely covered with asperities. Metathorax as large as abdominal segment I. Abdominal segments I–VI of almost equal length, abdominal segments VII–IX decreasing gradually to the terminal body part, segment X reduced to three anal lobes of those lateral are the largest, and dorsal the smallest (sometimes absent). Chaetotaxy weakly developed, setae short or medium. Prothorax (Figure 13B) with eleven *prns* (eight medium and three very short); two medium *ps* and one very short *eus.* Meso- and metathorax (Figure 13B) with one very short *prs*, two very short *pds*, one very short *as*, three *ss* (two medium and one very short), one medium *eps*, one medium *ps* and one very short *eus.* Pedal area with five *pda* (three medium and two very short). Abdominal segments I–VIII (Figure 13C, D) with one very short *prs*, three very short *pds* arranged along the posterior margin, two very short *ss*, two *eps* different in length, one medium *ps*, one medium *lsts* and two very short *eus.* Abdominal segment IX (Figure 13D) with three *ds* (one medium and two very short), all located close to the posterior margin, one medium *ps* and two very short *sts.* Each lateral anal lobe with two minute setae.

***Head capsule*** (Figures 13A, 14A–F) dark brown, narrowed bilaterally. Frontal suture visible. *Des_1–3,5_* very long, equal in length; *des_4_* three times shorter than *des_1_*. *Fs_1,4,5_* long; *fs_3_* very short. *Les_1_* and *les_2_* equal in length, slightly shorter than *des_1_*; *ves_1–2_* short; *pes_1–2_* short (Figure 14A). Antennae (Figure 14B) with sensorium (Se) slender, four times as long as wide, and three sensilla basiconica. Clypeus (Figure 14C) trapezium-shaped, anterior margin distinctly sinuated; both *cls* relatively long, *clss* absent. Labrum (Figure 14C) with slightly sinuate anterior margin; *lrs_1_* long, *lrs_2_* and *lrs_3_* medium. Epipharynx (Figure 14D) with three medium, finger-shaped *als* of almost equal length; two rod-like *ams* different in length; two finger-like *mes* of medium length; surface smooth; labral rods close to kidney-shaped. Mandibles (Figure 14E) conical, wide, with small protuberance in the middle of the cutting edge; one capilliform *mds*, medium, placed mediolaterally. Maxilla (Figure 14F) with one *stps* and two *pfs* equal in length; *mbs* very short; mala with six finger-like *dms* of different size (*dms_1–2_* medium, *dms_3–6_* long to very long), five *vms* different in length. Maxillary palpi: basal palpomere slightly wider than distal, both palpomeres almost equal in length. Prelabium (Figure 14F) cup-like with one relatively long *prms*; ligula with two *ligs* different in length; premental sclerite well visible, cup-shaped. Labial palpi elongated, one-segmented. Postlabium (Figure 14F) with medium *pms_1_*, long *pms_2_*, and medium *pms_3_*.

##### Description of pupa

**Figure 15.**
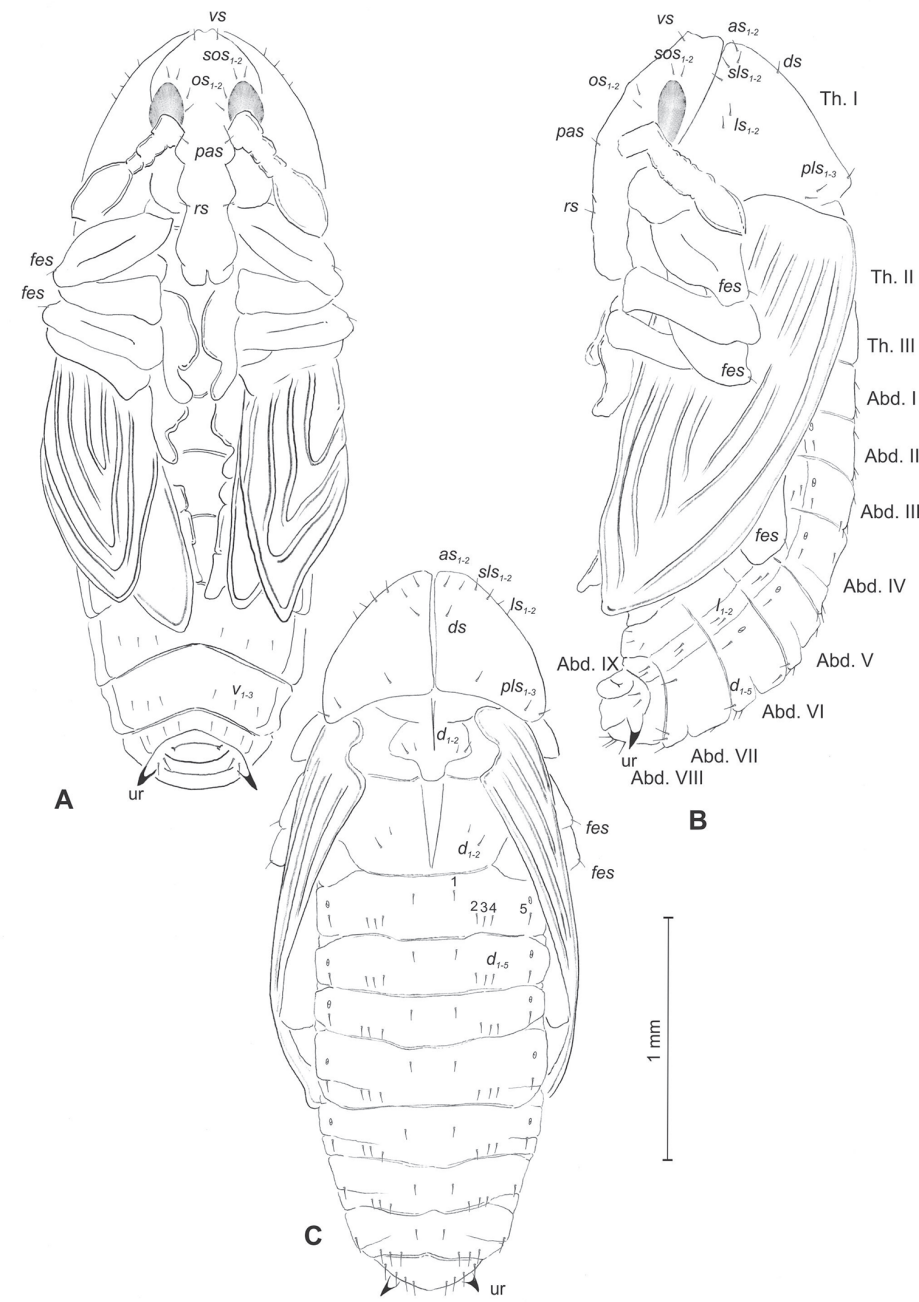
*Mecinus pyraster* pupa habitus and chaetotaxy **A** ventral view **B** lateral view **C** dorsal view. Abbreviations: Th. I–III – number of thoracic segments, Abd. I–IX – number of abdominal segments, ur – urogomphi, setae: *as* – apical, *d* – dorsal, *ds* – discal, *fes* – femoral, *l*, *ls* – lateral, *os* – orbital, *sls* – postantennal, *pls* – posterolateral, *rs* – rostral, *sls* – superlateral, *sos* – superorbital, *vs* – vertical.

(Figure 15A–C). ***Measurements*** (in mm). Head width: 0.53–0.63. Body width: 1.40–1.73. Body length: 3.33–4.26.

***Body*** elongated, white. Rostrum rather slender, about three times as long as wide, reaching almost up to mesocoxae. Antennae slender and elongated. Pronotum 1.8 times as wide as long. Urogomphi (ur) slender and rather elongated, conical, with sclerotised apex, reaching outline of the body, directed downward (Figure 14A–C).

***Chaetotaxy*** well developed, setae rather short. Head capsule with one *vs*, two *sos* equal in length, two *os* equal in length. Rostrum with one *rs* and one *sls* (Figure 14A). Pronotum with two *as*, one *ds*, two *sls*, two *ls*, and three *pls* (Figure 14A–C); equal in length (Figure 14B). Setae on head and rostrum as long as those on prothorax. Dorsal parts of meso- and metathorax with two setae different in length placed medially. Abdominal segments I–VIII with two medium setae laterally and three medium setae ventrally, distributed in regular lines. Dorsal parts of abdominal segments I–VII with five setae (*d_1_* placed anteromedially, *d_2–4_* posteromedially and *d_5_* located posterolaterally); abdominal segment VIII with only four very long setae dorsally. Abdominal segment IX with two micro-setae ventrally.

##### Biological notes.

This species is associated with some *Plantago* species (*P. lanceolata* L., *P. lagopus* L., *P. media* L.) (Hoffmann 1958; Sprick 2001). In west Palearctic larvae are most frequently found in the roots of *P. lanceolata*, boring channels in upper part of the root crown. Larger roots may inhabit several larvae. Pupation takes place during early summer in the pupal chamber situated in the upper part of larval channel. After emergence, adults overwinter in the soil litter nearby host plant.

##### Remarks and comparative notes.

This species is very common and widespread in the Palearctic region. It was also reported in North America (O’Brien and Wibmer 1982). The adult is distinctly variable in the size and shape of the body and vestiture within the same population. It differs from *M. circulatus* by the black integument and ventrite 5 in the male bearing a median tuft of hair. In larvae, the pronotum has eleven *prns* instead of eight, the pedal lobes have five *pda* instead of three, the anal lobes with two *ts* instead of one, the head with four *pes* instead of five, the mandible with one *mds* instead of two, the mala with five *vms* instead of four, and the *prms* are longer. The pupae also differ from those of *M. circulatus* by a different number of setae on head, pronotum and abdominal segments I–VII (see key to the pupae). However, morphological and molecular studies (I. Toševski, unpublished data) demonstrate a clear relationship between *M. pyraster* and *M. circulatus*.

#### Mecinus collaris

Taxon classificationAnimaliaColeopteraCurculionidae

group

8F9B676D-B07E-53B4-86B3-8A5FFF3FD0DD

##### Differential diagnosis.

**Larva.** (1) slightly pressed dorso-ventrally, cuticle densely tuberculate, premental sclerite, pedal lobes and spiracular area of meso- and metathorax dark pigmented; (2) pedal lobes prominent well isolated; (3) abdominal segment X reduced to three anal lobes of equal size; (4) thoracic spiracle bicameral; (5) abdominal setae short; (6) abdominal segments I–VII with four *pds* and two *ss* (abd. segment VIII with one *ss*); (7) head brown, flattened laterally; (8) frontal suture visible; (9) endocarina 1/2 of the frons; (10) *des_4_* minute or short; (11) presence of *fs_1_*; (12) absence of *fs_2_*; (13) *fs_3_* minute; (14) head with one stemma; (15) presence of *cls_1_*; (16) labial palpi two-segmented; (17) premental sclerite cup-like; (18) surface of postlabium smooth.

**Pupa.** (1) body elongated; (2) urogomphi slender, rather short, reaching outline of the body, directed downward; (3) rostrum moderately elongated; (4) setae different in length; (5) head with one *sos*; (6) rostrum with one *rs*; (7) pronotum with two *as*, one *ds*, one *sls*, one *ls*, four *pls*; (8) meso- and metanotum with two setae; (9) abdominal segments I–IV without setae dorsally; segments V–VII dorsally with five growing setae.

##### Remarks and comparative notes.

The adults of this monobasic group are easily distinguishable from all other species of *Mecinus* by several autapomorphies, such as rostrum short and wide, straight in lateral view, scrobe not reaching anterior margin of eye, elytra elongate, broad scales densely covering base of pronotum, epimera and episterna. In contrast, immatures have few autapomorphies, i.e., larvae are slightly pressed dorsoventrally, with a densely tuberculate cuticle, whereas premental sclerite, pedal lobes and spiracular area of meso- and metathorax are dark pigmented; the pupae have abdominal segments I–IV lacking setae dorsally, whereas segments V–VII dorsally possess five growing setae.

Presently, it is unclear to which species *M. collaris* is more closely related. The other species with short and straight rostrum, such as those of the *M. simus* group, do not apparently share other synapomorphies with *M. collaris*. In contrast, the larvae of the latter share the number of palpomeres of the labial palpi (two) and the shape of the thoracic spiracle (bicameral) and abdominal spiracles (unicameral) with the *M. janthinus* group. The pupae of *M. collaris* differ from all the others studied here by the dorsal setae of the abdominal segments because segments I–IV are without setae and segments V–VII have setae growing gradually.

#### Mecinus collaris

Taxon classificationAnimaliaColeopteraCurculionidae

Germar, 1821

12F52FCB-DC6B-522B-8D5A-41CBCF080F6E

##### Material examined.

26 L3 larvae and 21 pupae, Serbia, Zavojskojezero, Pirot, 15.07.2017, GPS 43°12.508'N, 22°35.590'E, 675 m., ex *Plantago media*, lgt. I. Toševski. Accession numbers of sequenced specimen MN992001.

##### Description of mature larva

**Figure 16.**
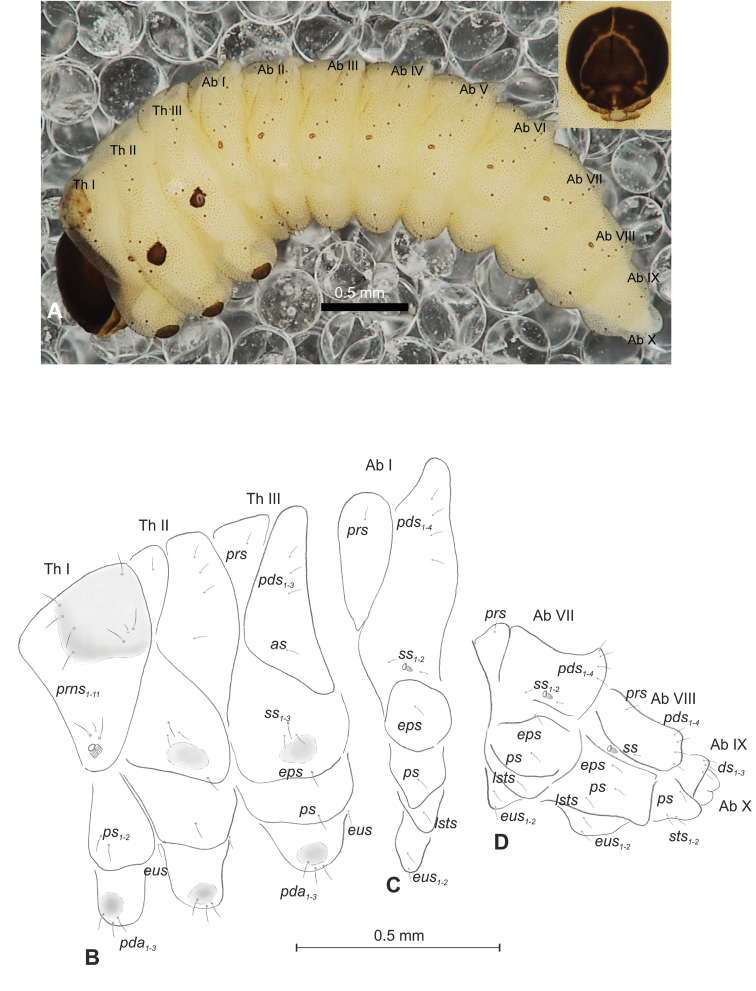
*Mecinus collaris* mature larva, habitus and chaetotaxy **A** habitus of the body and frontal view of the head **B** lateral view of thoracic segments **C** lateral view of abdominal segment I **D** lateral view of abdominal segments VII–X. Abbreviations: Th. I–III – number of thoracic segments, Abd. I–X – number of abominal segments, setae: *as* – alar, *ds* – dorsal, *eps* – epipleural, *eus* – eusternal, *lsts* – laterosternal, *pda* – pedal, *pds* – postdorsal, *prns* – pronotal, *prs* – prodorsal, *ps* – pleural, *ss* – spiracular, *sts* – sternal, *ts* – terminal.

**Figure 17.**
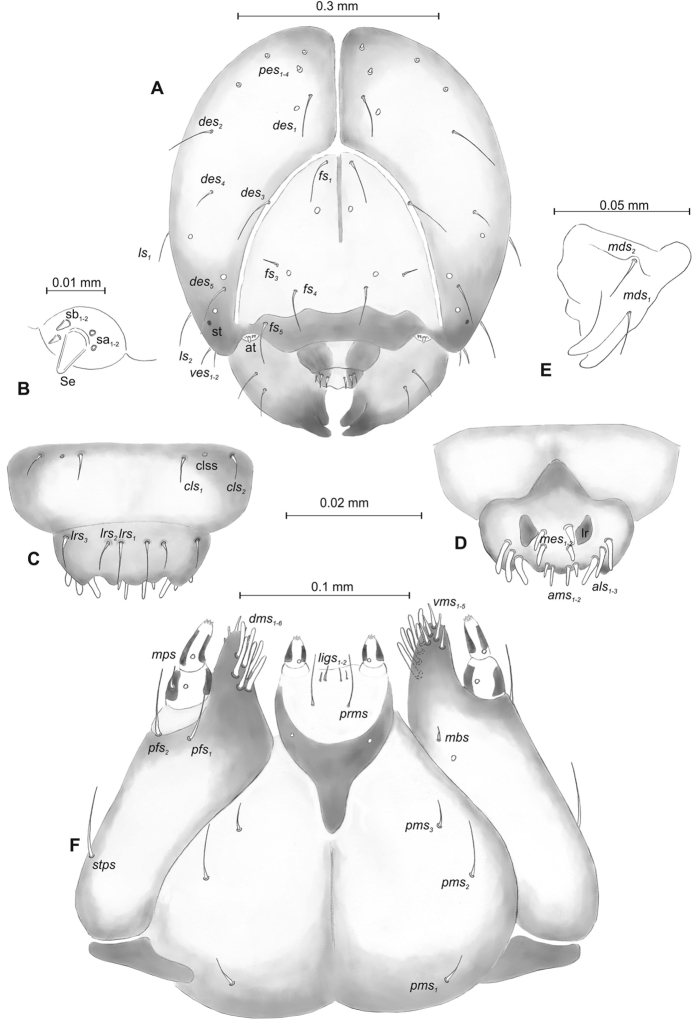
*Mecinus collaris* mature larva, head and mouth parts **A** head, frontal view **B** antenna **C** clypeus and labrum, dorsal view **D** epipharynx **E** left mandible **F** maxillolabial complex, ventral aspect. Abbreviations: *at* – antenna, *clss* – clypeal sensorium, *des* – dorsal epicranial, lr – labral rods, sa – sensillum ampullaceum, sb – sensillum basiconicum, Se – sensorium, st – stemmata, setae: *als* – anterolateral, *ams* – anteromedial, *cls* – clypeal, *dms* – dorsal malar, *fs* – frontal, *ligs* – ligular, *lrs* – labral, *ls* – lateral epicranial, *mbs* – malar basiventral, *mds* – mandibular, *mes* – median, *mxps* – maxillary palp, *pes* – postepicranial, *ves* – ventral, *pfs* – palpiferal, *pms* – postlabial, *prms* – prelabial, *stps* – stipal, *vms* – ventral malar.

(Figures 16A–D, 17A–F). ***Measurements*** (in mm). Body length: 2.00–3.66. Body width (metathorax): 0.80–1.16. Head width: 0.56–0.66.

***Body*** (Figure 16A–D) light yellow, slender, curved, slightly pressed dorso-ventrally. Premental sclerite, pedal lobes and spiracular area of meso- and metathorax dark pigmented. Chaetotaxy of thoracic segments relatively well developed, setae capilliform, variable in length, light yellow, on thoracic segments medium or relatively long, on abdominal segments I–IX short or medium. Prothorax (Figure 16B) with eleven *prns* (six long and one short placed on premental sclerite), next three close to spiracle; two medium *ps* and one short *eus.* Meso- and metathorax (Figure 16B) with one medium *prs*, three medium *pds* of equal length, one medium *as*, three medium *ss*, equal in length, one long *eps*, one long *ps* and one short *eus.* Pedal area with three *pda*, long or medium. Abdominal segments I–VIII (Figure 16C, D) with one short *prs*, four short *pds* arranged along the posterior margin, two minute*ss*, one short *eps*, one short *ps*, one short *lsts* and two short *eus.* Abdominal segment IX (Figure 16D) with three short *ds*, all located close to posterior margin, one short *ps* and two rather short *sts.* Anal lobes without setae.

***Head capsule*** (Figures 16A, 17A–C) dark brown, slightly narrowed bilaterally. *Des_1–3,5_* long, *des_4_* short; *des_4_* located in the central part of epicranium. *Fs_1_* long, *fs_3_* very short, *fs_4,5_* equal in length, almost as long as *des_1_*. *Les_1_* and *les_2_* slightly shorter than *des_1_*; two *ves*, and four *pes* very short (Figure 17A). Antennae (Figure 17B) with conical, elongated sensorium (Se), three times as long as wide, and two sensilla basiconica and two sensilla ampullacea. Clypeus (Figure 17C) trapezium-shaped, anterior margin almost straight; *cls_1-2_* medium, equal in length; *clss* well visible. Labrum (Figure 17C) narrow, trapezium-shaped, anterior margin distinctly sinuate; *lrs_1_* long, *lrs_2_* and *lrs_3_* medium. Epipharynx (Figure 17D) with three elongated, finger-like *als* of equal length; two medium, straight *ams*; two short finger-like *mes*; surface smooth; labral rods very short, close to kidney-shaped. Mandibles (Figure 17E) conical, rather wide; both *mds* capilliform, medium, equal in length, placed transversely. Maxilla (Figure 17F) with one *stps* and two *pfs* of equal length; *mbs* short; mala with six long rod-like *dms* of almost equal size, five *vms* various in length. Maxillary palpi: basal palpomere distinctly wider and slightly shorter than distal. Prelabium (Figure 17F) cup-like with one long *prms*; ligula with two minute *ligs*, premental sclerite well developed, with elongated median part. Labial palpi two-segmented; basal palpomere wider and shorter than distal. Postlabium (Figure 17F) with short *pms_1_*, long *pms_2_*, and short *pms_3_*.

##### Description of pupa

**Figure 18.**
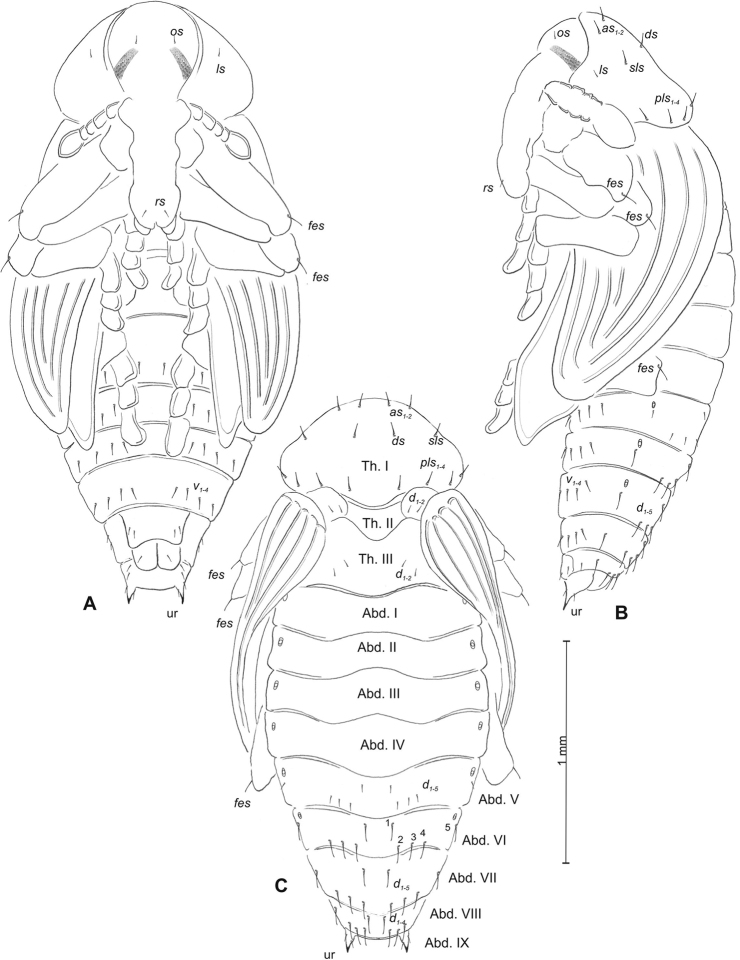
*Mecinus collaris* pupa habitus and chaetotaxy **A** ventral view **B** lateral view **C** dorsal view. Abbreviations: Th. I–III – number of thoracic segments, Abd. I–IX – number of abdominal segments, ur – urogomphi, setae: *as* – apical, *d* – dorsal, *ds* – discal, *fes* – femoral, *l*, *ls* – lateral, *pls* – posterolateral, *rs* – rostral, *sls* – superlateral, *sos* – superorbital.

(Figure 18A–C). ***Measurements*** (in mm). Head width: 0.30–0.36. Body width: 0.76–1.20. Body length: 1.66–2.33.

***Body*** moderately elongated, light yellowish. Rostrum moderately stout, about 2.1 times as long as wide, reaching up to mesocoxae. Antennae relatively short. Pronotum 1.6 times as wide as long. Mesonotum distinctly shorter than metanotum. Urogomphi (ur) short, conical, with sclerotised, sharp apex, slightly reaching outline of the body, directed downward (Figure 18A–C).

***Chaetotaxy*** sparse, setae short, unequal length. Head with only one *sos.* Rostrum with one *rs.* Setae on head and rostrum straight, much shorter than those on prothorax (Figure 18A). Pronotum with two *as*, one *sls*, one *ls*, one *ds* and four *pls.* Dorsal parts of meso- and metathorax with two setae placed medially. Dorsal parts of abdominal segments I–IV without setae; segments V–VII with five setae (*d_1_* placed anteromedially, *d_2–4_* posteromedially, *d_5_* posterolaterally, under spiracle); segment VIII with four setae dorsally. Abdominal segments I–VIII with four long setae ventrally, distributed in regular lines. Abdominal segment IX with two micro-setae ventrally, and next two on urogomphi.

##### Biological notes.

Larvae feed on various species of *Plantago*, but mainly on *P. media* L. and *P. maritima* L. *Plantago lanceolata*, *P. coronopus* L., and *P. major* L. are also known as host plants. The adults are active from mid-spring following the growth of the flowering stems of the host plant. The female oviposits inside the upper parts of the flowering stem that are covered with floral spikes, which induces clearly visible oblong galls. Very often, several larvae develop in a single flowering shoot. The larvae pupate inside the galls and the adults emerge during summer. Overwintering takes place in the soil litter near the host plant.

##### Remarks.

This species, which is widely distributed in the Palearctic region except in North Africa (Alonso-Zarazaga et al. 2017), is unique in *Mecinus*, being characterised by long elytra and whitish to orange, wide scales covering the base of the pronotum, the epimera and the episterna. For the differences from the immatures of the other species, see the remarks for the group.

#### Mecinus janthinus

Taxon classificationAnimaliaColeopteraCurculionidae

group

2EBBEF93-91B2-5B88-9372-2194DDC865D8

##### Differential diagnosis.

**Larva.** (1) body densely covered with asperities; (2) pedal lobes weakly isolated; (3) abdominal segment X reduced to three anal lobes of unequal size; (4) thoracic spiracle bicameral; (5) abdominal setae medium to very long, distinclty growing from abdominal segment I to VIII; (6) abdominal segments I–VIII with four *pds* and usually three *ss*; (7) head brown, flattened laterally; (8) frontal suture distinct; (9) endocarina 4/5 of the frons; (10) *des_4_* usually shorter than *des_1_*; (11) presence of *fs_1_*; (12) absence of *fs_2_*; (13) *fs_3_* as long as half of *fs_4_*; (14) head with two stemmata; (15) presence of *cls_1_*; (16) labial palpi two-segmented; (17) premental sclerite cup-like; (18) surface of postlabium densely covered with asperities.

**Pupa.** (1) body very slender and elongated; (2) urogomphi rather elongated, distinctly reaching outline of the body, directed outside; (3) rostrum elongated and slender; (4) setae more or less elongated; (5) head with one *vs*, two *sos*, two *os*; (6) rostrum with one or two *sls* and without or with one *rs*; (7) pronotum with two *as*, one *ds*, two *sls*, two *ls*, three or four *pls*; (8) meso- and metanotum with two or three setae; (9) abdominal segments I–VII dorsally with six or seven elongated, growing setae.

##### Remarks and comparative notes.

The adult of this species is characterised by elytra distinctly elongate, dorsal integument black or blue, sometimes with metallic reflections.

The shape of the body together with the colour of the dorsal integument are characters that this group shares only with *M. heydenii*. These two groups include the species of *Mecinus* not living on *Plantago*. Nevertheless, they seem to be not closely related on the basis of both a phylogenetic study of the adults and of molecular data as well as the examination of the immatures. The adults of the species related to *M. janthinus* are distinguishable from those related to *M. heydenii* by the less curved rostrum in lateral view, the shape of the penis, the distinctly longer flagellum and the completely unusual shape of the spermatheca that is reminiscent of the Cionini. The larvae and pupae differ in a series of characters in the chaetotaxy. Moreover, the immatures of this group possess some autapomorphies, i.e., in larvae four *pds* and usually three *ss* on the abdominal segments I–VIII and the surface of postlabium densely covered with asperities, and in pupae, the more or less elongated setae on the body and the abdominal segments I–VII dorsally with six or seven elongated, growing setae.

#### Mecinus janthinus

Taxon classificationAnimaliaColeopteraCurculionidae

Germar, 1821

BFDC1297-8E5B-51C2-B7BE-0B190FD35FFE

##### Material examined.

9 L3 larvae and 8 pupae, Serbia, Mihajlovac, 5.07.2009, 44°21.541'N, 22°28.650'E, 130 m., ex *L. vulgaris*; Serbia, Negotin, Tamnič, 2.08. 2007, 44°06.033'N, 22°30.105'E, 126 m., ex *L. vulgaris*; 8 pupae, Serbia, Mihajlovac, 5.07.2009, 44°21.683'N, 22°28.697'E, 125 m., ex *L. vulgaris*; 1 pupa, Serbia, DonjaKamenica, Kalna, 22.08.2011, 43°29.450'N, 22°19.712'E, 278 m., ex *L. vulgaris*. All collected by I. Toševski. Accession numbers of sequenced specimen MN992005.

##### Description of mature larva

**Figure 19.**
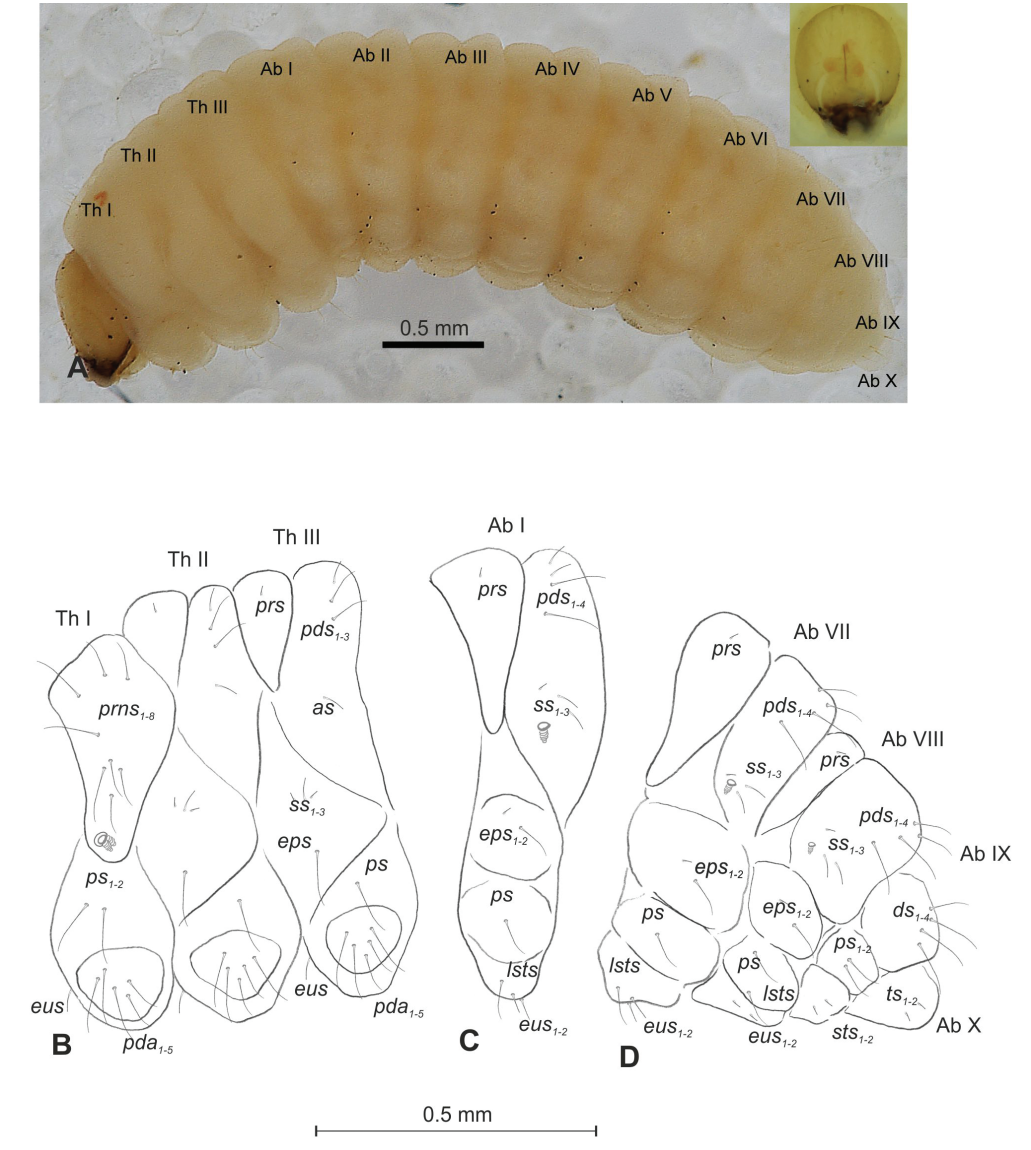
*Mecinus janthinus* mature larva, habitus and chaetotaxy **A** habitus of the body and frontal view of the head **B** lateral view of thoracic segments **C** lateral view of abdominal segment I **D** lateral view of abdominal segments VII–X. Abbreviations: Th. I–III – number of thoracic segments, Abd. I–X – number of abominal segments, setae: *as* – alar, *ds* – dorsal, *eps* – epipleural, *eus* – eusternal, *lsts* – laterosternal, *pda* – pedal, *pds* – postdorsal, *prns* – pronotal, *prs* – prodorsal, *ps* – pleural, *ss* – spiracular, *sts* – sternal, *ts* – terminal.

**Figure 20.**
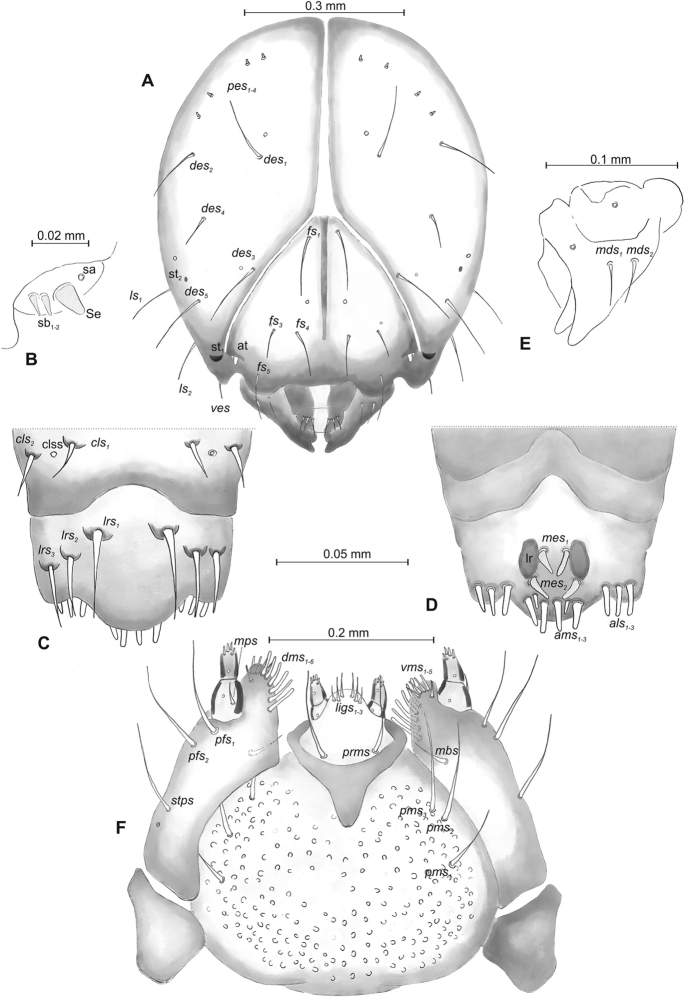
*Mecinus janthinus* mature larva, head and mouth parts **A** head, frontal view **B** antenna **C** clypeus and labrum, dorsal view **D** epipharynx **E** left mandible **F** maxillolabial complex, ventral aspect. Abbreviations: *at* – antenna, *clss* – clypeal sensorium, *des* – dorsal epicranial, lr – labral rods, sa – sensillum ampullaceum, sb – sensillum basiconicum, Se – sensorium, st – stemmata, setae: *als* – anterolateral, *ams* – anteromedial, *cls* – clypeal, *dms* – dorsal malar, *fs* – frontal, *ligs* – ligular, *lrs* – labral, *ls* – lateral epicranial, *mbs* – malar basiventral, *mds* – mandibular, *mes* – median, *mxps* – maxillary palp, *pes* – postepicranial, *ves* – ventral, *pfs* – palpiferal, *pms* – postlabial, *prms* – prelabial, *stps* – stipal, *vms* – ventral malar.

(Figures 19A–D, 20A–F). ***Measurements*** (in mm). Body length: 4.00–4.75. Body width (metathorax and abdominal segments I–II): 1.10–1.25. Head width: 0.50–0.57.

***Body*** (Figure 19A–D) yellowish, very slender, densely covered with asperities. Prothorax smaller than meso- and metathorax. Abdominal segments I–V of almost equal length; segments VI–IX decreasing gradually to the terminal body part; segment X reduced to three anal lobes of those lateral are the largest, and dorsal the smallest (sometimes absent). Chaetotaxy well developed, setae capilliform, variable in length, greyish or yellow. Prothorax (Figure 19B) with eight long *prns* of equal length; two long *ps* and one short *eus.* Meso- and metathorax (Figure 19B) with one very short *prs*, three *pds*, variable in length (*pds_1_* short, *pds_2–3_* medium), one short *as*, three short *ss*, one long *eps*, one long *ps* and one long *eus.* Pedal area with five long *pda.* Abdominal segments I–VIII (Figure 19C, D) with one very short *prs*, four *pds* of different length (on segments I–V: *pds_1–2_* short, *pds_3–4_* long; on segments VI–VIII all *pds* very long, almost equal in length) and arranged along posterior margin; one minute and two medium *ss*, one short and one long *eps*, one long *ps*, one long *lsts* and two medium *eus.* Abdominal segment IX (Figure 19D) with four very long *ds*, all located close to the posterior margin, two long *ps* and two short *sts.* Each of lateral anal lobe with two minute setae.

***Head capsule*** (Figures 19A, 20A–F) yellow, distinctly narrowed bilaterally. *Des_1–3,5_* very long, equal in length; *des_4_* half the length of other *des*; *des_4_* medially. *Fs_1,4,5_* long, *fs_3_* medium. *Les_1_* and *les_2_* long, equal in length; one *ves*, and four *pes* short (Figure 20A). Two stemmata of different size. Antennae (Figure 20B) with sensorium (Se) conical, twice as long as wide, and three sensilla of different types: one sa and two sb. Clypeus (Figure 20C) trapezium-shaped, anterior margin distinctly concave; two *cls* relatively long, located on protuberances; *clss* placed medially between *cls.* Labrum (Figure 20C) with sinuate anterior margin; *lrs_1-3_* almost equal in length, all placed on protuberances. Epipharynx (Figure 20D) with three medium, finger-shaped *als* of almost equal length; two finger-like, different in length *ams*; two medium finger-like *mes*; surface smooth; labral rods close to kidney-shaped. Mandibles (Figure 20E) conical, wide, an elongated protuberance in the middle of the cutting edge; both *mds* capilliform, medium, equal in length, placed mediolaterally. Maxilla (Figure 20F) with one *stps* and two *pfs* of equal length; *mbs* medium; mala with six rod-like *dms* of almost equal size, five *vms* equal in length. Maxillary palpi: basal palpomere distinctly wider than distal, both of almost equal length. Prelabium (Figure 20F) cup-like with one very long *prms*; ligula with three medium *ligs*; premental sclerite clearly visible, cup-shaped. Labial palpi two-segmented; basal palpomere slightly wider and distinctly shorter than distal. Postlabium (Figure 20F) with three capilliform medium to long *pms*.

##### Description of pupa

**Figure 21.**
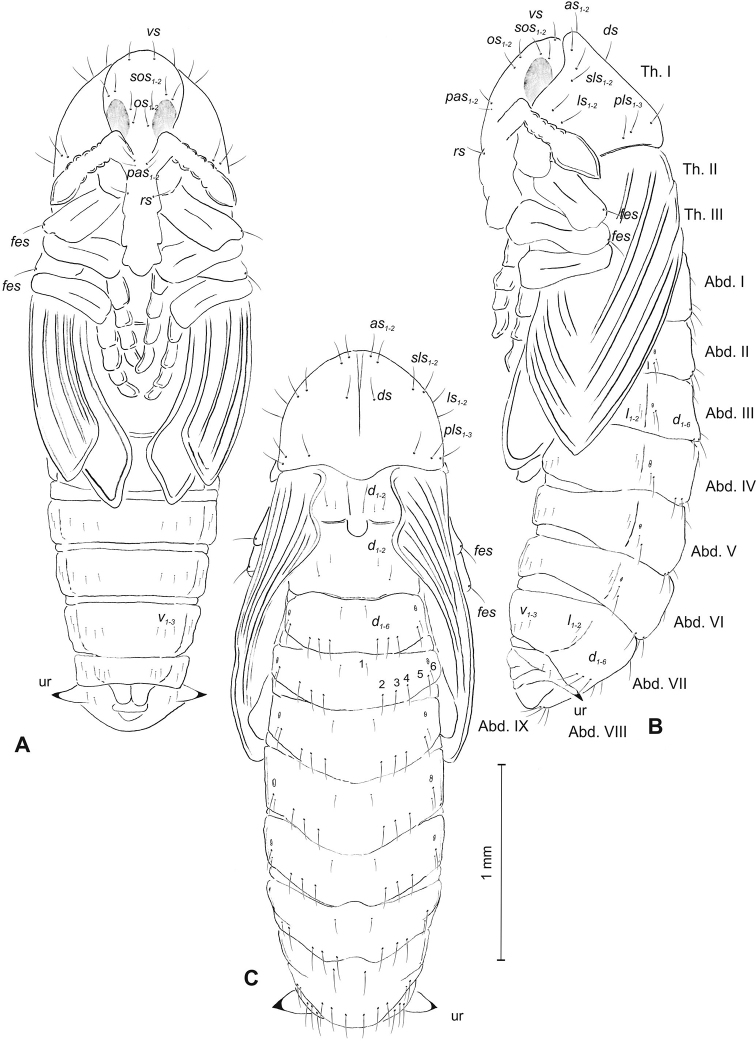
*Mecinus janthinus* pupa habitus and chaetotaxy **A** ventral view **B** lateral view **C** dorsal view. Abbreviations: Th. I–III – number of thoracic segments, Abd. I–IX – number of abdominal segments, ur – urogomphi, setae: *as* – apical, *d* – dorsal, *ds* – discal, *fes* – femoral, *l*, *ls* – lateral, *os* – orbital, *sls* – postantennal, *pls* – posterolateral, *rs* – rostral, *sls* – superlateral, *sos* – superorbital, *vs* – vertical.

(Figure 21A–C). ***Measurements*** (in mm). Head width: 0.46–0.56. Body width: 1.16–1.50. Body length: 3.70–4.05.

***Body*** elongated, white. Rostrum slender, about 3.4 times as long as wide, reaching almost up to mesocoxae. Antennae slender and elongated. Pronotum 1.1 times as wide as long. Mesonotum slightly shorter than metanotum. Urogomphi (ur) slender and elongated, conical, with sclerotised apex, distinctly reaching outline of the body, directed outside (Figure 21A–C).

***Chaetotaxy*** well developed, setae short or medium long. Head with one *vs*, two *sos*, two *os* and two *pas*. Rostrum with one *rs* placed medially. All setae of head equal in length (Figure 21A, B). Pronotum with two *as*, one *ds*, two *sls*, two *ls*, and three *pls* (Figure 21B, C). All setae on pronotum elongated, equal in length (Figure 21C). Dorsal parts of meso- and metathorax with two setae placed medially. Abdominal segments I–VIII with two setae laterally and three medium long setae ventrally. Dorsal parts of abdominal segments I–VII with six setae (*d_1_* placed anteromedially, *d_2–4_* placed posteromedially, *d_5–6_* posterolaterally); segment VIII with five very long setae dorsally. Abdominal segment IX with two micro-setae ventrally.

##### Biological notes.

The host plant of *M. janthinus* is the yellow toadflax, *Linaria vulgaris* Mill. This species is distributed in temperate regions of the eastern Palearctic region, inhabiting lowlands and hilly slopes up to 500 m altitude. From beginning of the 1990s, *M. janthinus* was introduced as biological control agent for the control of invasive toadflaxes in North America (Toševski et al. 2018). The adults emerge in early March and feed intensively on the newly growing shoots of the host plant. Oviposition occurs on actively growing shoots, and the preferred oviposition site is the widest part of the stem. Females lay one or, rarely, two eggs per shoot. This species is a true stem borer with larval feeding and mining in the central part of the stem. The adults overwinter in the stems of the host plant inside an elongated pupal chamber built by the last instar larva prior to pupation.

##### Remarks and comparative notes.

*Mecinus janthinus* is largely distributed in northern, central and southeastern Europe, Russia from the western borders to southern central Siberia, the Caucasian states, and Turkey. This species was introduced in North America for the biological control of toadflaxes in 1991–1999 (Wilson et al. 2005). The adults can be easily confused with *M. janthiniformis*, both sympatric in part of their range of distribution, since the differences between them are few and subtle. In contrast, the larvae of these two species show numerous differences in the number of setae in many parts of the body, such as the head, antenna, pronotum and thoracic segments (see key).

#### Mecinus janthiniformis

Taxon classificationAnimaliaColeopteraCurculionidae

Toševski & Caldara, 2011

C622AE8B-331F-542E-95CF-60DDA1B32B96

##### Material examined.

2 L3 larvae, Mecedonia, Prilep, 25.07.2017, (41°17.354'N, 21°29.983'E, 618 m.) ex *Linaria dalmatica macedonica* 1 L3 larva, 4 pupae, Bulgaria, Harmanli, 17.08.2008, 41°53.117'N, 25°52.373'E, 310 m., ex *Linaria genistifolia* 12 L3 larva, Bulgaria, Harmanli, 17.07.2011, 41°53.117'N, 25°52.373'E, 310 m., ex *L. genistifolia*; 2 L3 larvae, 1 pupa, Bulgaria, Slatino, 7.08.2011, 42°09.981'N, 23°02.371'E, 390 m., ex *L. genistifolia*; 1 pupa, Serbia, Kalna, 1.09.2010., 43°29.450'N, 22°19.712'E, 278 m., ex *L. genistifolia*; 3 pupae, Serbia, Bovansko Jezero, Aleksinac, 12.08.2010, 43°37.735'N, 21°42.917'E, 231 m., ex *L. genistifolia*; North Macedonia, Veles, 10.09.2009, 41°44.332'N, 21°46.893'E, 201 m., ex *L. genistifolia*; 1 pupa, Serbia, Vranje, Golemo Selo, 20.08.2009, 42°44.203'N, 21°50.696'E, 523 m., ex *L. genistifolia*; 3 pupae, Bulgaria, Slatino, 7.08.2007, 42°09.981'N, 23°02.371'E, 390 m., ex *L. genistifolia*. All collected by I. Toševski. Accession numbers of sequenced specimen MN992006.

##### Description of mature larva

**Figure 22.**
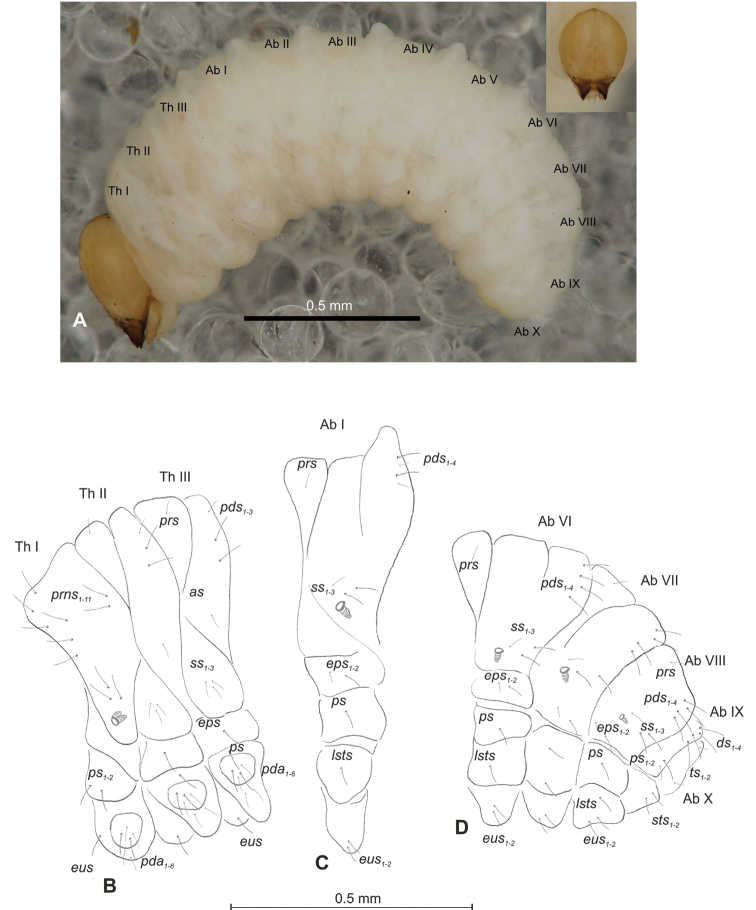
*Mecinus janthiniformis* mature larva, habitus and chaetotaxy **A** habitus of the body and frontal view of the head **B** lateral view of thoracic segments **C** lateral view of abdominal segment I **D** lateral view of abdominal segments VII–X. Abbreviations: Th. I–III– number of thoracic segments, Abd. I–X – number of abominal segments, setae: *as* – alar, *ds* – dorsal, *eps* – epipleural, *eus* – eusternal, *lsts* – laterosternal, *pda* – pedal, *pds* – postdorsal, *prns* – pronotal, *prs* – prodorsal, *ps* – pleural, *ss* – spiracular, *sts* – sternal, *ts* – terminal.

**Figure 23.**
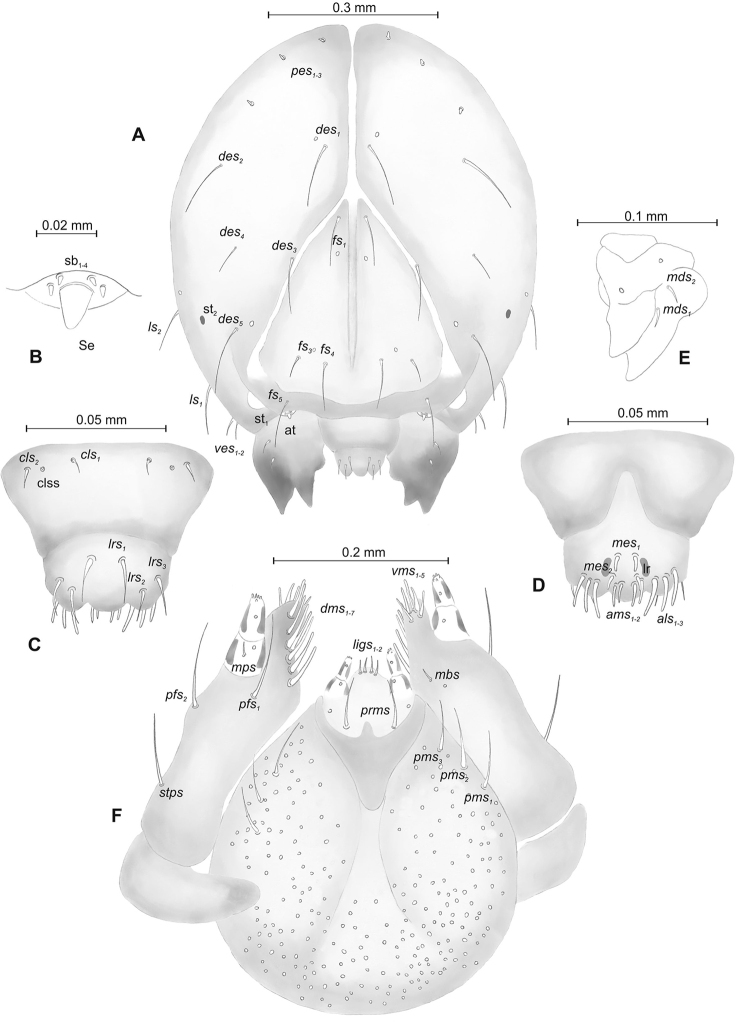
*Mecinus janthiniformis* mature larva, head and mouth parts **A** head, frontal view **B** antenna **C** clypeus and labrum, dorsal view **D** epipharynx **E** left mandible **F** maxillolabial complex, ventral aspect. Abbreviations: *at* – antenna, *clss* – clypeal sensorium, *des* – dorsal epicranial, lr – labral rods, sb – sensillum basiconicum, Se – sensorium, st – stemmata, setae: *als* – anterolateral, *ams* – anteromedial, *cls* – clypeal, *dms* – dorsal malar, *fs* – frontal, *ligs* – ligular, *lrs* – labral, *ls* – lateral epicranial, *mbs* – malar basiventral, *mds* – mandibular, *mes* – median, *mxps* – maxillary palp, *pes* – postepicranial, *ves* – ventral, *pfs* – palpiferal, *pms* – postlabial, *prms* – prelabial, *stps* – stipal, *vms* – ventral malar.

(Figures 22A–D, 23A–F). ***Measurements*** (in mm). Body length: 1.66–2.90. Body width (abdominal segments I–II): 0.66–1.10. Head width: 0.53–0.67.

***Body*** (Figure 22A–D) yellowish. Prothorax smaller than meso- and metathorax. Abdominal segments I–VII of almost equal length; segments VIII and IX decreasing gradually to the terminal body part; segment X reduced to three anal lobes of those lateral are the largest, and dorsal the smallest (sometimes absent). Dorsum of abdominal segments I–VI divided into three lobes; on seventh into two lobes. Chaetotaxy well developed, setae various in length. Prothorax (Figure 22B) with eleven long *prns*; two medium *ps* and one medium *eus.* Meso- and metathorax (Figure 22B) with one short *prs*, three *pds* (*pds_1_* short, *pds_2–3_* medium), one medium *as*, three medium *ss*, one medium *eps*, one medium *ps* and one medium *eus.* Pedal area with six *pda* of different length (four of them placed on well isolated pedal sclerite). Abdominal segments I–VIII (Figure 22C, D) with one short *prs*, four *pds* (on segments I–V: *pds_1,3_* medium, *pds_2,4_* short; on segments VI–VIII all *pds* very long, equal in length), always arranged along the posterior margin, one minute and two long *ss*, one short and one medium e*ps*, one medium *ps*, one medium *lsts* and two medium *eus.* Abdominal segment IX (Figure 22D) with four long *ds* located close to posterior margin, two *ps* different in length, and two short *sts.* Each of anal lobe (abd. segment X) with two minute setae.

***Head capsule*** (Figures 22A, 23A–F) dark yellow, narrowed bilaterally. *Des_1–3,5_* very long, equal in length, *des_4_* twice shorter than other *des*; *des_4_* medially. *Fs_1_* as long as *des_1_, fs_3_* short, *fs_4,5_* long. *Les_1_* and *les_2_* equal in length, slightly shorter than *des_1_*; two *ves* and three *pes* very short (Figure 23A). Two stemmata of different size. Antennae (Figure 23B) with sensorium (Se) conical, twice as long as wide, and four sensilla basiconica (sb). Clypeus (Figure 23C) trapezium-shaped, anterior margin concave; two medium *cls*, *clss* clearly visible. Labrum (Figure 23C) with sinuate anterior margin; *lrs_1_* long, *lrs_2_* and *lrs_3_* medium. Epipharynx (Figure 23D) with three relatively long, finger-shaped *als* of almost equal length; two finger-shaped *ams*, equal in length; two rod-like *mes* of medium length; surface smooth; labral rods short, kidney shaped. Mandibles (Figure 23E) conical, wide, with a small protuberance in the middle of the cutting edge; both *mds* capilliform, relatively short, equal in length, placed mediolaterally. Maxilla (Figure 23F) with one *stps* and two *pfs* of equal length; *mbs* very short; mala with seven long finger-like *dms* of almost equal size, five *vms* different in length. Maxillary palpi: basal palpomere wider than distal, both of almost equal length. Prelabium (Figure 23F) cup-shaped with one very long *prms*; ligula with two relatively long *ligs*; premental sclerite clearly visible, cup-like. Labial palpi two-segmented; basal palpomere distinctly wider than distal, both almost equal in length. Postlabium (Figure 23F) with three medium *pms*.

##### Description of pupa

**Figure 24.**
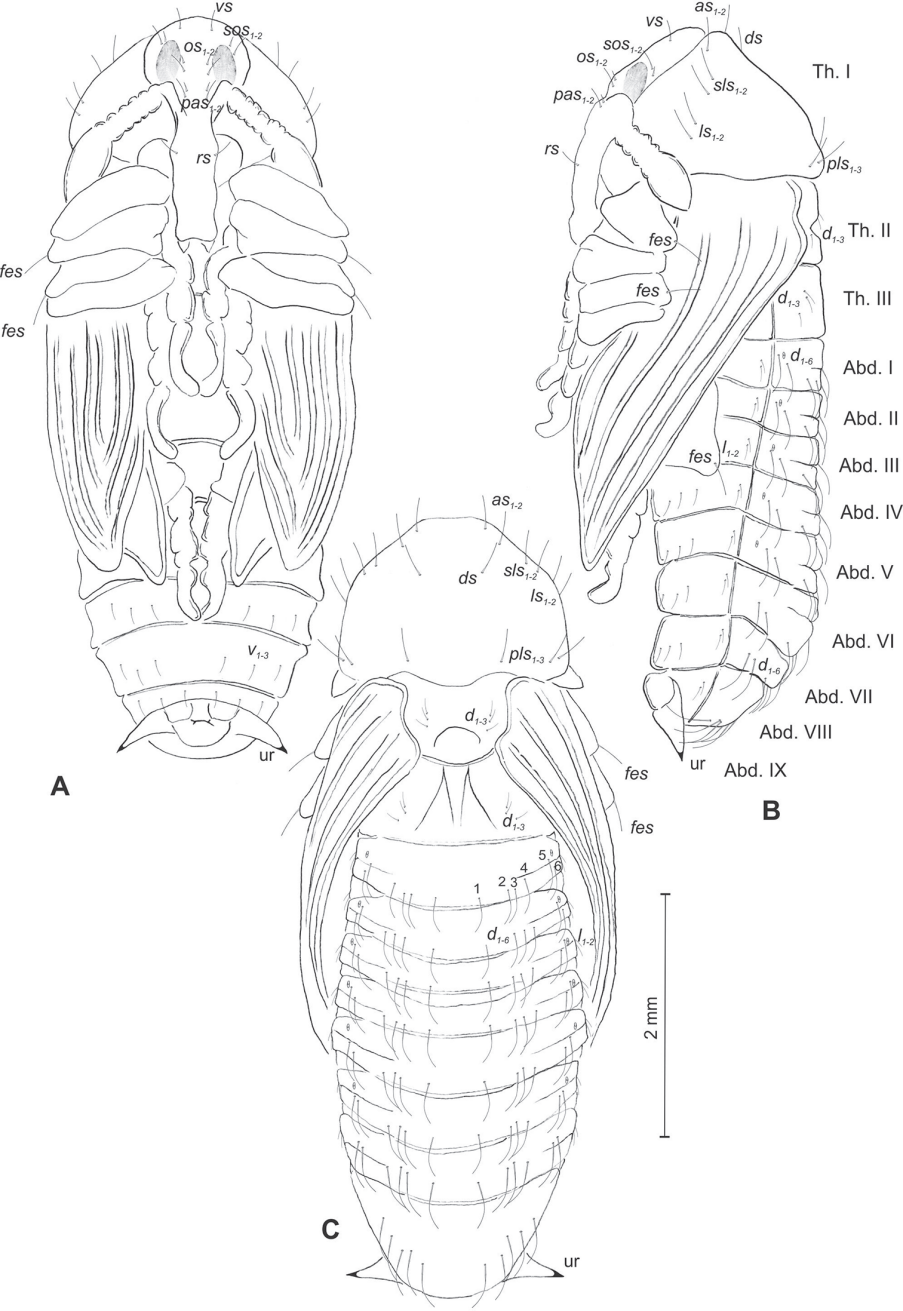
*Mecinus janthiniformis* pupa habitus and chaetotaxy **A** ventral view **B** lateral view **C** dorsal view. Abbreviations: Th. I–III – number of thoracic segments, Abd. I–IX – number of abdominal segments, ur – urogomphi, setae: *as* – apical, *d* – dorsal, *ds* – discal, *fes* – femoral, *l*, *ls* – lateral, *os* – orbital, *sls* – postantennal, *pls* – posterolateral, *rs* – rostral, *sls* – superlateral, *sos* – superorbital, *vs* – vertical.

(Figure 24A–C). ***Measurements*** (in mm). Head width: 0.46–0.66. Body width: 1.20–1.66. Body length: 3.22–4.16.

***Body*** elongated, white. Rostrum slender, about four times as long as wide, reaching almost up to mesocoxae. Antennae slender and elongated. Pronotum 1.3 times as wide as long. Urogomphi (ur) slender and elongated, conical, with sclerotised apex, distinctly reaching outline of the body, directed outside (Figure 24A, C).

***Chaetotaxy*** well developed, setae medium long or elongated, unequal length. Head with one long *vs*, two *sos* different in length, two *os* different in length and two *sls* different in length. Rostrum with one *rs* (Figure 24A). Pronotum with two *as*, one *ds*, two *sls*, two *ls*, and three *pls* (Figure 24B, C). All setae on prothorax elongated, equal in length (Figure 24C). Setae on head and rostrum shorter than those on prothorax. Dorsal parts of meso- and metathorax with three setae placed medially. Abdominal segments I–VIII with two setae placed laterally and three medium long setae ventrally. Dorsal parts of abdominal segments I–VII with six setae (*d_1–4_* placed posteromedially, *d_5–6_* posterolaterally); segment VIII with five very long setae dorsally. Abdominal segment IX with two micro-setae ventrally.

##### Biological notes.

The host plants of *M. janthiniformis* are *Linaria genistifolia* (L.) Mill. and *L. dalmatica* (L.) Mill., as well as all variable forms and hypothetical hybrids between these two plant species. *Mecinus janthiniformis* inhabits stands from lowlands to mountain pastures and meadows up to 1500 m. At the beginning of the 1990s, this species was introduced as a biological control agent for the control of invasive toadflaxes in North America (Toševski et al. 2018). Adults emerge in early spring and feed on the apical part of newly growing shoots. The females lay eggs over the next three months on the upper part of the main stem, including the lateral branches of the plant. Oviposition and larval development induce a slightly elongate gall in which the larvae pupate. The adults of this species overwinter inside the main stem of the host plant or inside induced galls on lateral branches (Toševski et al. 2011).

##### Remarks and comparative notes.

The distribution of *M. janthiniformis* follows that of the two host plants, *L. genistifolia* (L.) Mill. and *L. dalmatica* (eastern part of central and southeastern Europe to southern central Siberia, the northern Caucasian states and Turkey). Its separation from *M. janthinus* at the species level was clearly shown based on very careful biological and genetic studies (Toševski et al. 2011), but unfortunately, easy identification is only possible by collecting the specimens together with their host plants. Usually, in *M. janthiniformis*, the body is larger (length 3.2–6.0 mm), the apical part of the rostrum in females in lateral view is more curved, the punctures of the pronotum are slightly smaller and more densely adpressed, and the scales of the elytral interstriae are denser, arranged in two rows on part of several interstriae. The larvae of these two species show numerous differences in the number of setae in many parts of the body, whereas the differences are few in the pupae (see keys).

#### Mecinus sicardi

Taxon classificationAnimaliaColeopteraCurculionidae

Hustache, 1920

1F9A5873-FBF6-5574-AD9C-89CC02C7F8FB

##### Material examined.

6 L3 larvae and 1 pupa, France, Provence-Alpes-Côte d’Azur, Alpes Maritimes, road Èze-La Turbie, 20.07.2014, on *Antirrhinum latifolium* Mill. stems, lgt. and det. R. Caldara. Accession numbers of sequenced specimen MN992007.

##### Description of mature larva

**Figure 25.**
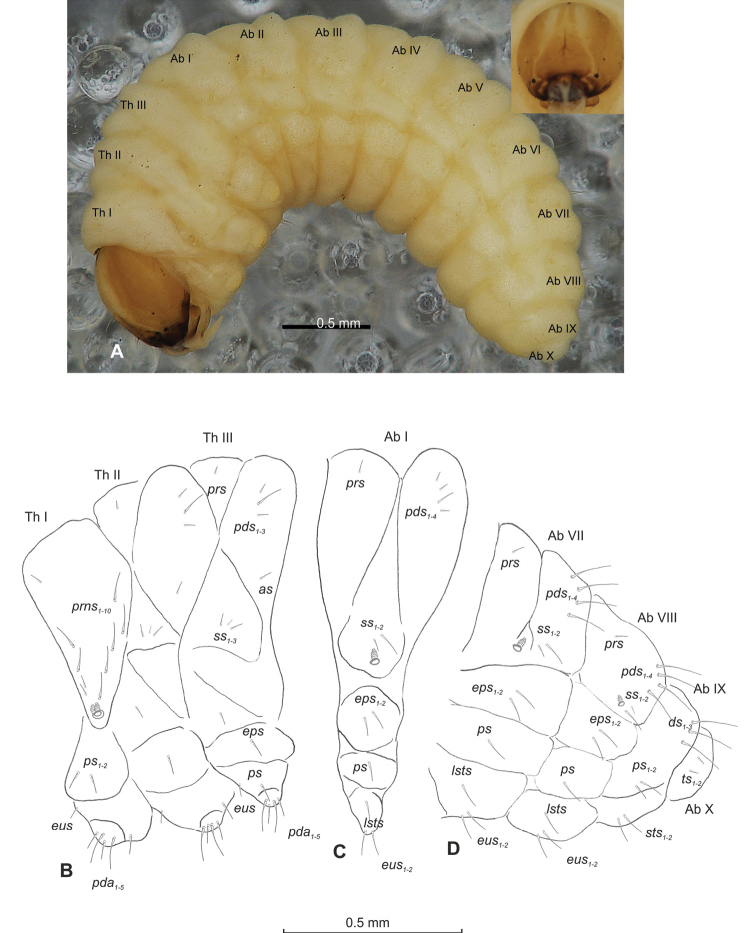
*Mecinus sicardi* mature larva, habitus and chaetotaxy **A** habitus of the body and frontal view of the head **B** lateral view of thoracic segments **C** lateral view of abdominal segment I **D** lateral view of abdominal segments VII–X. Abbreviations: Th. I–III – number of thoracic segments, Abd. I–X – number of abominal segments, setae: *as* – alar, *ds* – dorsal, *eps* – epipleural, *eus* – eusternal, *lsts* – laterosternal, *pda* – pedal, *pds* – postdorsal, *prns* – pronotal, *prs* – prodorsal, *ps* – pleural, *ss* – spiracular, *sts* – sternal, *ts* – terminal.

**Figure 26.**
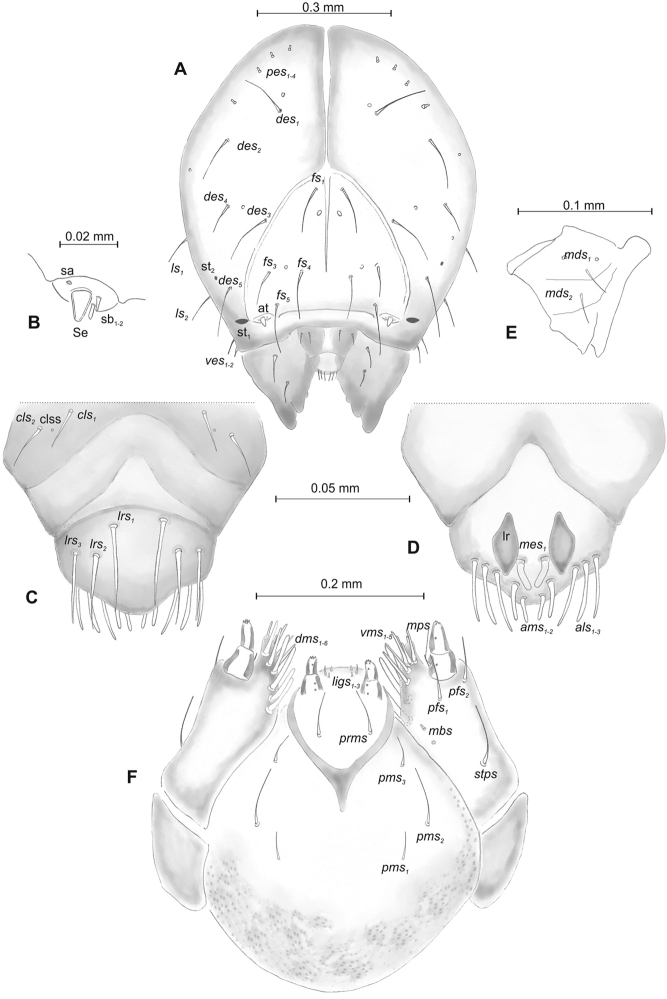
*Mecinus sicardi* mature larva, head and mouth parts **A** head, frontal view **B** antenna **C** clypeus and labrum, dorsal view **D** epipharynx **E** left mandible **F** maxillolabial complex, ventral aspect. Abbreviations: *at* – antenna, *clss* – clypeal sensorium, *des* – dorsal epicranial, lr – labral rods, sa – sensillum ampullaceum, sb – sensillum basiconicum, Se – sensorium, st – stemmata, setae: *als* – anterolateral, *ams* – anteromedial, *cls* – clypeal, *dms* – dorsal malar, *fs* – frontal, *ligs* – ligular, *lrs* – labral, *ls* – lateral epicranial, *mbs* – malar basiventral, *mds* – mandibular, *mes* – median, *mxps* – maxillary palp, *pes* – postepicranial, *ves* – ventral, *pfs* – palpiferal, *pms* – postlabial, *prms* – prelabial, *stps* – stipal, *vms* – ventral malar.

(Figures 25A–D, 26A–F). ***Measurements*** (in mm). Body length: 2.71–3.75. Body width (abdominal segments I–II): 1.10–1.25. Head width: 0.60–0.65.

***Body*** (Figure 25A–D) yellowish, slender. All thoracic segments almost equal in length. Abdominal segments I–V of almost equal length; segments VI–IX decreasing gradually to the terminal body part; segment X reduced to three anal lobes of those lateral are the largest, and dorsal the smallest (sometimes absent). Chaetotaxy weakly developed, setae capilliform, variable in length, yellow. Prothorax (Figure 25B) with ten *prns* of unequal length (seven medium length, three short); two medium *ps* and one medium *eus.* Meso- and metathorax (Figure 25B) with one very short *prs*, three *pds*, different in length (*pds_1,3_* very short, *pds_2_* medium); one short *as*, three short *ss*, one medium long *eps*, one medium long *ps* and one medium *eus.* Pedal area with five *pda*, different in length. Abdominal segments I–VIII (Figure 25C, D) with one short *prs*, four *pds* of different length (*pds_1,2,4_* short, *pds_3_* medium; all *pds* on segments VI–VIII very long, equal in length) arranged along the posterior margin, one short and one medium *ss*, two medium *eps*, one medium *ps*, one medium *lsts* and two relatively long *eus.* Abdominal segment IX (Figure 25D) with three very long *ds*, all located close to the posterior margin, one medium *ps* and two medium *sts.* Each of lateral anal lobe with two minute setae.

***Head capsule*** (Figures 25A, 26A–F) yellow, distinctly narrowed bilaterally. *Des_1–3,5_* equal in length, *des_4_* twice shorter than other *des. Fs_1,4,5_* long, equal in length, *fs_3_* medium. *Les_1_* and *les_2_* medium, equal in length; two *ves* short; four *pes* spine-like (Figure 26A). Two stemmata of different size. Antennae (Figure 26B) with sensorium (Se) conical, twice as long as wide, located medially, and three sensilla of different types: one sa and two sb. Clypeus (Figure 26C) trapezium-shaped, anterior margin slightly concave; two medium *cls*, located posteromedially; *clss* clearly visible. Labrum (Figure 26C) close to semi-circular, anterior margin sinuate; *lrs_1-3_* almost equal in length. Epipharynx (Figure 26D) with three rod-shaped *als* of almost equal length; two medium, finger-like *ams*; one medium, finger-like *mes*; surface smooth; labral rods short and relatively wide. Mandibles (Figure 26E) conical, wide, with small protuberance in the middle of the cutting edge; both *mds* capilliform, medium, equal in length, placed transversely. Maxilla (Figure 26F) with one *stps* and two *pfs* of equal length; *mbs* short; mala with six rod-like *dms* of almost equal size, five *vms* different in size. Maxillary palpi: basal palpomere slightly wider than distal, both of almost equal length. Prelabium (Figure 26F) cup-like with one long *prms*; ligula with three minute *ligs*; premental sclerite clearly visible, cup-shaped, posterior extension with acute apex. Labial palpi two-segmented; basal palpomere distinctly wider than distal, both of almost equal length. Postlabium (Figure 26F) with three *pms*; *pms_1_* and *pms_3_* short, *pms_2_* three times as long as *pms_1_*.

##### Description of pupa

**Figure 27.**
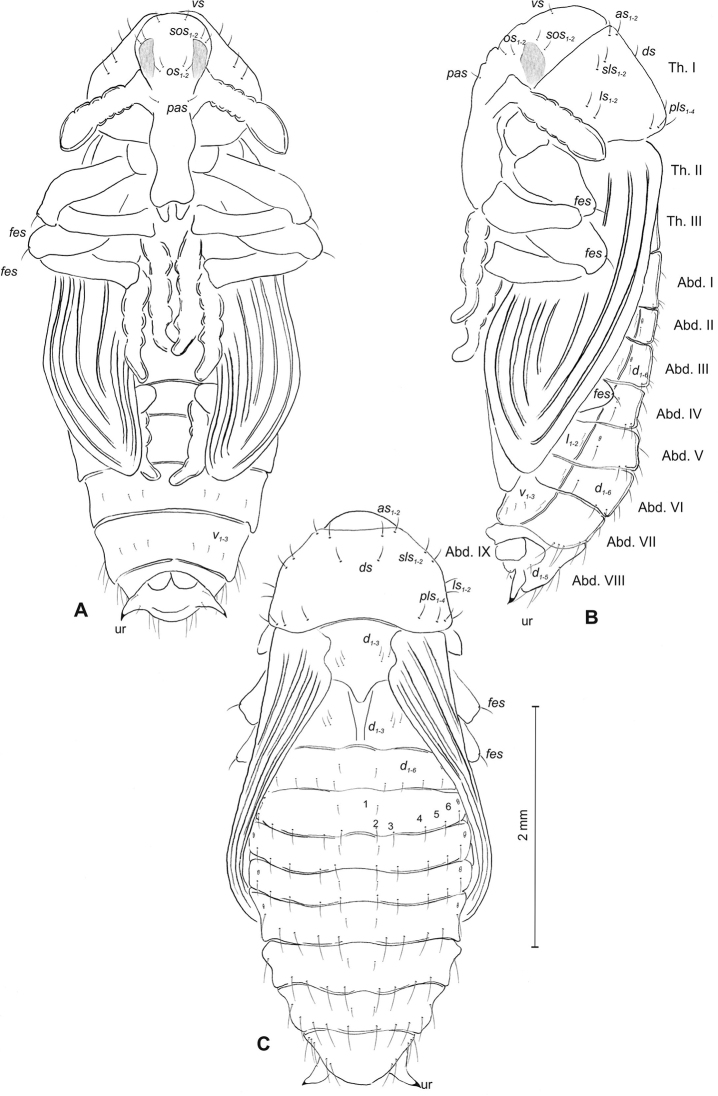
*Mecinus sicardi* pupa habitus and chaetotaxy **A** ventral view **B** lateral view **C** dorsal view. Abbreviations: Th. I–III – number of thoracic segments, Abd. I–IX – number of abdominal segments, ur – urogomphi, setae: *as* – apical, *d* – dorsal, *ds* – discal, *fes* – femoral, *l*, *ls* – lateral, *os* – orbital, *sls* – postantennal, *pls* – posterolateral, *rs* – rostral, *sls* – superlateral, *sos* – superorbital, *vs* – vertical.

(Figure 27A–C). ***Measurements*** (in mm). Head width: 0.60–0.70. Body width: 1.75–2.00. Body length: 3.75–4.50.

***Body*** elongated, white. Rostrum moderately slender, about 3.5 times as long as wide, reaching up to mesocoxae. Antennae elongated. Pronotum 1.8 times as wide as long. Urogomphi (ur) slender, conical, with sclerotised apex, both directed outside, distinctly reaching outline of the body (Figure 27A–C).

***Chaetotaxy*** setae medium or elongated. Head with one *vs*, two *sos* and two *os*. Rostrum with one *pas*. Setae on head and rostrum straight, as long as those on prothorax (Figure 27A). Pronotum with two *as*, two *sls*, two *ls*, one *ds* and four *pls.* Dorsal parts of meso- and metathorax with three setae equal in length setae placed medially. Abdominal segments I–VIII with three very short setae ventrally and two setae laterally. Dorsal parts of abdominal segments I–VII with six setae growing gradually from segment I to VII (*d_1_* placed anteromedially, *d_2–5_* placed posteromedially, *d_6_* posterolaterally); segment VIII with five elongated setae dorsally. Abdominal segment IX with two micro-setae ventrally.

##### Biological notes.

The host plant of this species, at least in Côte d’Azur, is *Antirrhinum latifolium* Mill. As reported by Caldara and Fogato (2013), larvae feed on the larger stems of the plant and dig tunnels, causing at most very small lateral deformations. They pupate in summer, and adults stay inside the plant until the spring of the following year. Before pupation, however, the mature larvae leave the main tunnel, which runs longitudinally, and produce a small oblique tunnel that ends just in proximity of the external cuticle of the stem. Therefore, when leaving their cells, adults have only to bore a thin layer, although in the meantime, the plant has become dry and hard.

##### Remarks and comparative notes.

The adults of this rare species, with a narrow range of distribution – in fact, it is known in a few localities of southeastern Spain, eastern and southern France, and north-western Italy – differ from the other species studied here by the black elytra instead of blue. Due to this character, this species may be superficially confused with *M. pyraster*, from which it is easily distinguishable by the elytral vestiture composed of scales that are uniformly arranged and are all recumbent. The rostrum (in dorsal view) is distinctly wider, and the pronotum has sides slightly more rounded and is usually widest towards the middle. Finally, ventrite 5 of the male lacks a tuft of hairs, and the shape of the penis is different.

The larvae of this species differ from the others of the group by the abdominal segments I–VIII with two *ss* (instead of three) and asperities covering only the posterior part of postlabium, whereas pupae differ in having the rostrum with only one *sls* (instead of two) and without *rs* and the pronotum with four *pls* (instead of three).

#### Mecinus heydenii

Taxon classificationAnimaliaColeopteraCurculionidae

group

62F3EA79-7951-5CBE-A302-28147A7578FD

##### Differential diagnosis.

**Larva.** (1) cuticle of the body tuberculate; (2) pedal lobes weakly isolated; (3) abdominal segment X reduced to three anal lobes of those lateral are the largest, and dorsal the smallest (sometimes absent); (4) thoracic spiracle unicameral; (5) abdominal setae very short to medium, become progressively longer from abdominal segment I to VIII; (6) abdominal segments I–VIII with three *pds* and two *ss*; (7) head brown, flattened laterally; (8) frontal suture poorly or well visible; (9) endocarina 4/5 of the frons; (10) *des_4_* three times shorter than *des_1_*; (11) *fs_1_* usually absent; (12) absence of *fs_2_* except one species; (13) *fs_3_* very short; (14) head with two stemmata; (15) absence of *cls_1_*; (16) labial palpi one-segmented; (17) premental sclerite U-shaped; (18) surface of postlabium smooth.

**Pupa.** (1) body very slender and elongated; (2) urogomphi short, only slightly reaching outline of the body, directed downward; (3) rostrum slender and elongated; (4) setae minute; (5) head with one *os*; (6) rostrum with without or with one *sls* and one *rs*; (7) pronotum with without or with up to two *as*, one *ds*, without or with one *sls*, without or with one *ls*, three *pls*; (8) meso- and metanotum with three setae; (9) abdominal segments I–VII with three or five setae dorsally.

##### Remarks and comparative notes.

The adults of this group are characterised by the rostrum in the basal half strongly and abruptly curved, the elytra distinctly elongate, and the dorsal integument black or blue, usually with metallic reflections apart from several characters of the male and female genitalia. In immatures, the autapomorphies seem limited to a U-shaped premental sclerite in larvae.

This group seems more closely related to the *M. janthinus* group than to other groups of *Mecinus* in both morphological characters (shape of body and colour of dorsal integument) and biology (hosts in Plantaginaceae other than *Plantago*). However, the species of the *M. heydenii* group clearly differ from those of the *M. janthinus* group by the rostrum being strongly curved in the basal half and by the shape of the penis and spermatheca. The study of immatures also did not show close relationships between these two unique groups living on Antirrhineae, since the species of the *M. heydenii* group have one palpomere on the labial palpi instead of two, and all spiracles are unicameral. The pupae also differ somewhat in the shape of the urogomphi, which are shorter, only slightly reaching the outline of the body, and directed downward. The setae of the head and pronotum are also shorter, and the dorsal setae of abdominal segments I–VII are less numerous.

#### Mecinus heydenii

Taxon classificationAnimaliaColeopteraCurculionidae

Wencker, 1866

11489967-CABB-5F2E-9366-4AD39495153F

##### Material examined.

4 L3 larvae and 6 pupae, Serbia, Negotin, 1.07.2017, 44°16.610'N, 22°30.480'E, 71 m., ex *L. vulgaris*, lgt. I. Toševski. Accession numbers of sequenced specimen MN992002.

##### Description of mature larva

**Figure 28.**
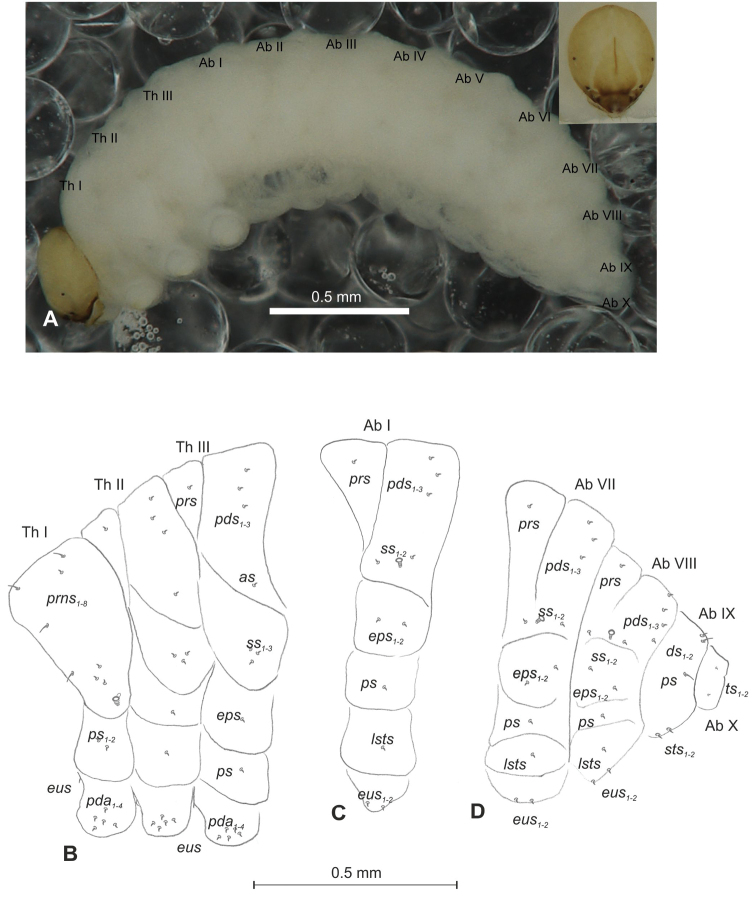
*Mecinus heydenii* mature larva, habitus and chaetotaxy **A** habitus of the body and frontal view of the head **B** lateral view of thoracic segments **C** lateral view of abdominal segment I **D** lateral view of abdominal segments VII–X. Abbreviations: Th. I–III – number of thoracic segments, Abd. I–X – number of abominal segments, setae: *as* – alar, *ds* – dorsal, *eps* – epipleural, *eus* – eusternal, *lsts* – laterosternal, *pda* – pedal, *pds* – postdorsal, *prns* – pronotal, *prs* – prodorsal, *ps* – pleural, *ss* – spiracular, *sts* – sternal, *ts* – terminal.

**Figure 29.**
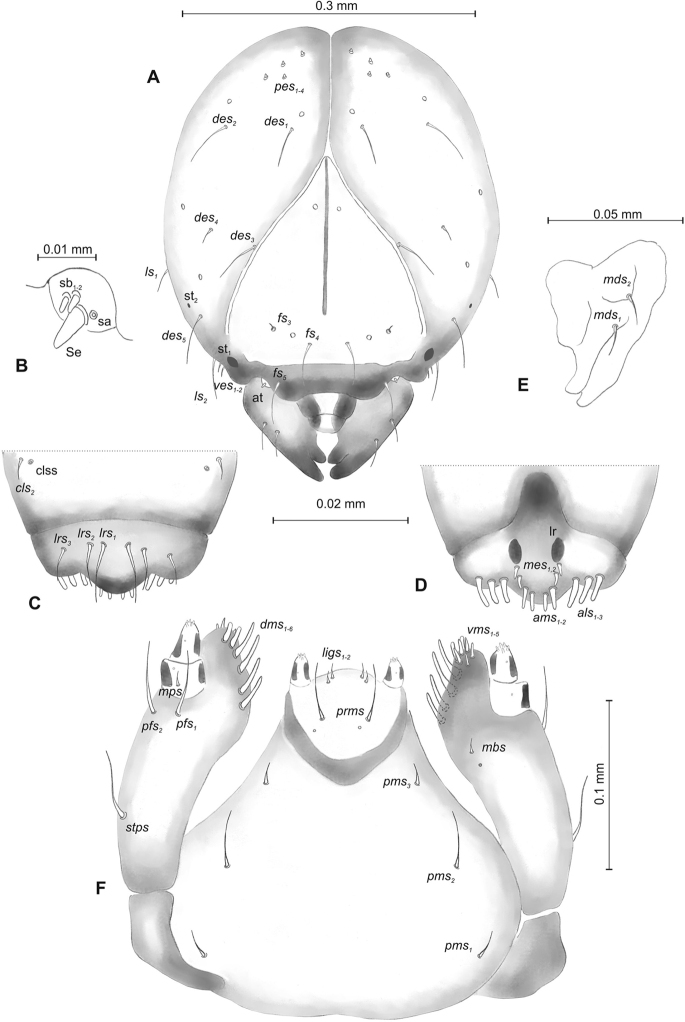
*Mecinus heydenii* mature larva, head and mouth parts **A** head, frontal view **B** antenna **C** clypeus and labrum, dorsal view **D** epipharynx **E** left mandible **F** maxillolabial complex, ventral aspect. Abbreviations: *at* – antenna, *clss* – clypeal sensorium, *des* – dorsal epicranial, lr – labral rods, sa – sensillum ampullaceum, sb – sensillum basiconicum, Se – sensorium, st – stemmata, setae: *als* – anterolateral, *ams* – anteromedial, *cls* – clypeal, *dms* – dorsal malar, *fs* – frontal, *ligs* – ligular, *lrs* – labral, *ls* – lateral epicranial, *mbs* – malar basiventral, *mds* – mandibular, *mes* – median, *mxps* – maxillary palp, *pes* – postepicranial, *ves* – ventral, *pfs* – palpiferal, *pms* – postlabial, *prms* – prelabial, *stps* – stipal, *vms* – ventral malar.

(Figures 28A–D, 29A–F). ***Measurements*** (in mm). Body length: 2.16–2.66. Body width (metathorax or abdominal segments I–II): 0.83–1.00. Head width: 0.30–0.33.

***Body*** (Figure 28A–D) white-yellowish, very slender. Chaetotaxy weakly developed, setae (except pronotum and dorsal part of abdominal segment IX) extremely short, difficult to observe. Prothorax (Figure 28B) with four medium and four very short *prns*, two very short *ps* and one very short *eus.* Meso- and metathorax (Figure 28B) with one short *prs*, three short *pds*, one very short *as*, three minute *ss*, one very short *eps*, one very short *ps* and one very short *eus.* Pedal area with four very short *pda.* Abdominal segments I–VIII (Figure 28C, D) with one very short *prs*, three very short *pds* arranged along the posterior margin, two minute *ss*, two very short *eps*, one very short *ps*, one very short *lsts* and two very short *eus.* Abdominal segment IX (Figure 28D) with two *ds* (one medium and one very short), all located close to the posterior margin, one medium *ps* and two very short *sts.* Each lateral anal lobe with two minute setae.

***Head capsule*** (Figures 28A, 29A–F) pale yellow, distinctly narrowed bilaterally. Frontal suture poorly visible. *Des_1–3,5_* very long, equal in length; *des_4_* four times shorter than other *des_1_*. *Fs_1_* and *fs_2_* absent, *fs_3_* very short, *fs_4_* and *fs_5_* long. *Les_1_* shorter than *les_2_*; two *ves* and four *pes* short (Figure 29A). Antennae (Figure 29B) with sensorium (Se) conical, thrice as long as wide, and three sensilla of different types: one sa and two sb. Clypeus (Figure 29C) trapezium-shaped, anterior margin slightly concave; *cls_2_* relatively long; *clss* clearly visible. Labrum (Figure 29C) with sinuate anterior margin; *lrs_1_* long, *lrs_2_* slightly shorter than *lrs_1_*, *lrs_3_* two times shorter than *lrs_1_*. Epipharynx (Figure 29D) with three medium, finger-shaped *als* of almost equal length; two rod-like, different in length *ams*; two finger-like *mes* of medium length; surface smooth; labral rods close to kidney-shaped. Mandibles (Figure 29E) conical, wide, with a small protuberance in the middle of the cutting edge; both *mds* capilliform, medium, equal in length, placed mediolaterally. Maxilla (Figure 29F) with one *stps* and two *pfs* long, of equal length; *mbs* very short; mala with six finger-like *dms* different in length (*dms_1,2_* medium, *dms_3–6_* long to very long), five *vms* different in length. Maxillary palpi: basal palpomere distinctly wider than distal, both of almost equal length. Prelabium (Figure 29F) almost rounded with one very long *prms*; ligula with two *ligs* different in length; premental sclerite clearly visible, U-shaped. Postlabium (Figure 29F) with short *pms_1_*, long *pms_2_*, and short *pms_3_*.

##### Description of pupa

**Figure 30.**
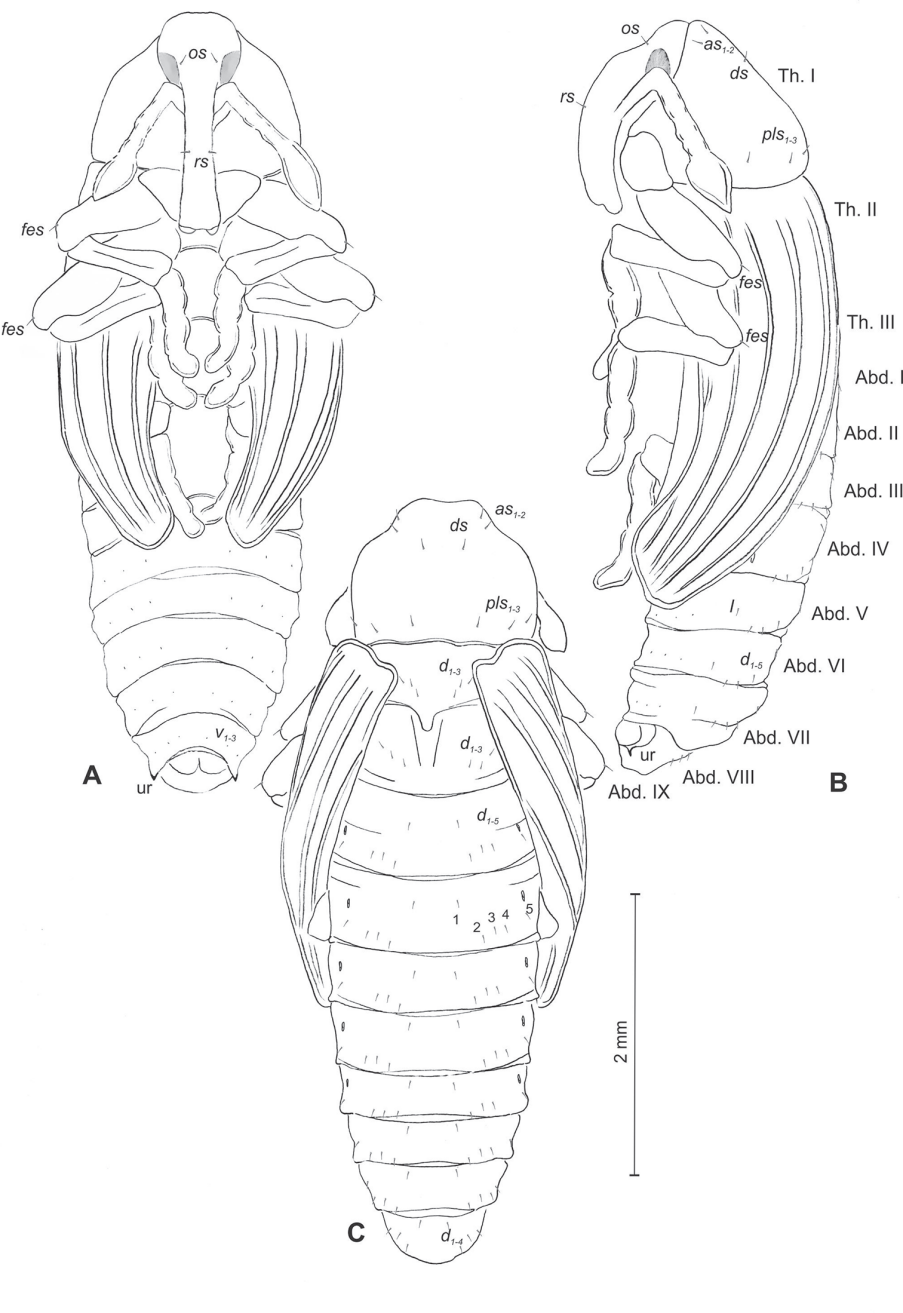
*Mecinus heydenii* pupa habitus and chaetotaxy **A** ventral view **B** lateral view **C** dorsal view. Abbreviations: Th. I–III – number of thoracic segments, Abd. I–IX – number of abdominal segments, ur – urogomphi, setae: *as* – apical, *d* – dorsal, *ds* – discal, *fes* – femoral, *l*, *ls* – lateral, *os* – orbital, *pls* – posterolateral, *rs* – rostral.

(Figure 30A–C). ***Measurements*** (in mm). Head width: 0.30–0.60. Body width: 0.73–1.13. Body length: 2.33–2.93.

***Body*** elongated, white. Rostrum slender, about five times as long as wide, but reaching up only to procoxae. Antennae slender and elongated. Pronotum 1.4 times as wide as long. Urogomphi (ur) very short, conical, with sclerotised apex, reaching outline of the body, directed downward (Figure 30A–C).

***Chaetotaxy*** sparse, setae short, unequal length. Head with one *os*. Rostrum with one *rs* placed medially. Setae on head and rostrum straight, as long as those on prothorax (Figure 30A). Pronotum with two *as*, one *ds* and three *pls.* Dorsal parts of meso- and metathorax with three setae placed medially. Abdominal segments I–VIII with one seta laterally and three very short setae ventrally. Dorsal parts of abdominal segments I–VII with five setae (*d_1_* placed anteromedially, *d_2–4_* posteromedially, *d_5_* posterolaterally); segment VIII with four setae dorsally. Abdominal segment IX with two micro-setae ventrally.

##### Biological notes.

This monophagous species is associated with yellow toadflax, *Linaria vulgaris* Mill. The adults are active from early spring, following the appearance of the first growing shoots of its host plant. The adults exhibit extreme cryptic behavior, which makes them difficult to collect. Oviposition occurs on actively growing young shoots, usually in the top or middle part of the stem. Females often lay several eggs distributed along the host plant shoot. Oviposition provokes primitive shoot swelling and hypertrophy that leads to the formation of a pseudo-gall of the young shoot. Larval development occurs inside this pseudo-gall, and pupation takes place in larval chambers prepared very close to the stem surface. Emerged adults stay inside the stem until August, when all adults leave their host plant within a two-week period. Overwintering takes place in the soil litter near the host plant.

##### Remarks and comparative notes.

This species is widely distributed in Europe and is the only one of its group present in northern Europe, from Germany to Sweden. The adult is distinguishable by the rostrum very strongly curved from base to apex, especially before antennal insertion, in both sexes. However, it is somewhat difficult to morphologically separate this taxon from the two cryptic species *M. peterharrisi* and *M. laeviceps*. They are well distinguishable, however, by molecular and biological data (Toševski et al. 2014).

The study of the immatures allowed us to add numerous other interesting differences: larvae of *M. heydenii* differ from those of *M. laeviceps* by the pronotum with eight *prns* (instead of nine), the thoracic segments with three *pds* (instead of two), each pedal lobe with four *pda* (instead of three), *pds* of abdominal segments I–VIII distinctly smaller, and the head with four *pes* (instead of one). Both species differ from *M. peterharrisi* by *fs_1_* and *fs_2_* absent and the antennae with two *sb* (instead of four).

The pupae of the three species are also slightly different in the presence or lack of some setae on the rostrum and pronotum (without *sls* and *ls* in *M. heydenii* and *M. peterharrisi*, respectively) and femora (with *fes* in *M. heydenii* and *M. laeviceps*), and their number in the abdominal segments.

#### Mecinus laeviceps

Taxon classificationAnimaliaColeopteraCurculionidae

Tournier, 1873

001C3D5C-8F13-55A2-8124-E4A453F6A0D7

##### Material examined.

8 L3 larvae and 10 pupae, Serbia, Slankamen, 22.06.2017 45°08.343'N, 20°15.042'E, 177 m., ex *Linaria genistifolia*, lgt. I. Toševski. Accession numbers of sequenced specimen MN992003.

##### Description of mature larva

**Figure 31.**
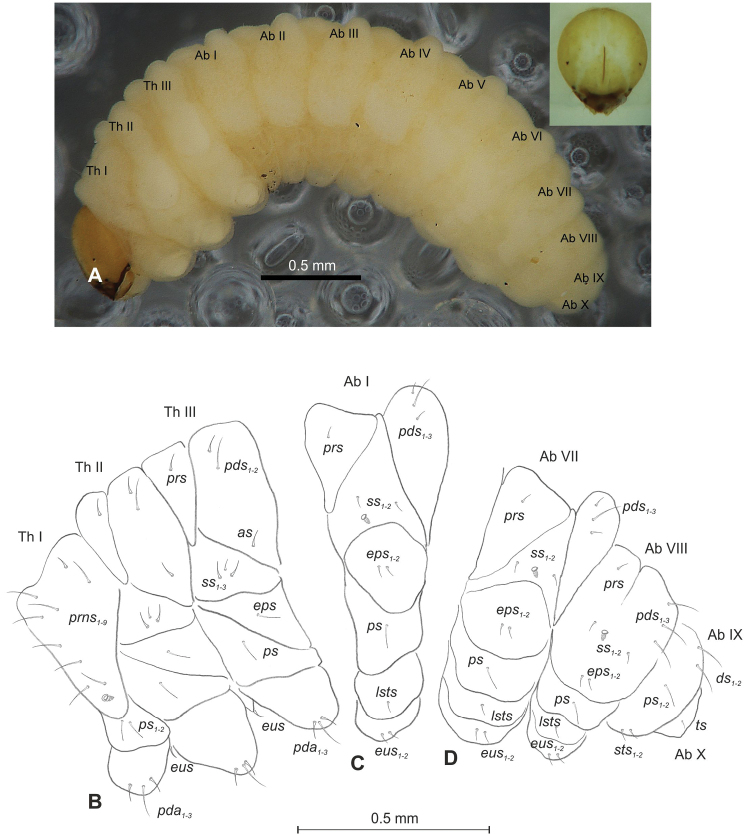
*Mecinus laeviceps* mature larva, habitus and chaetotaxy **A** habitus of the body and frontal view of the head **B** lateral view of thoracic segments **C** lateral view of abdominal segment I **D** lateral view of abdominal segments VII–X. Abbreviations: Th. I–III – number of thoracic segments, Abd. I–X – number of abominal segments, setae: *as* – alar, *ds* – dorsal, *eps* – epipleural, *eus* – eusternal, *lsts* – laterosternal, *pda* – pedal, *pds* – postdorsal, *prns* – pronotal, *prs* – prodorsal, *ps* – pleural, *ss* – spiracular, *sts* – sternal, *ts* – terminal.

**Figure 32.**
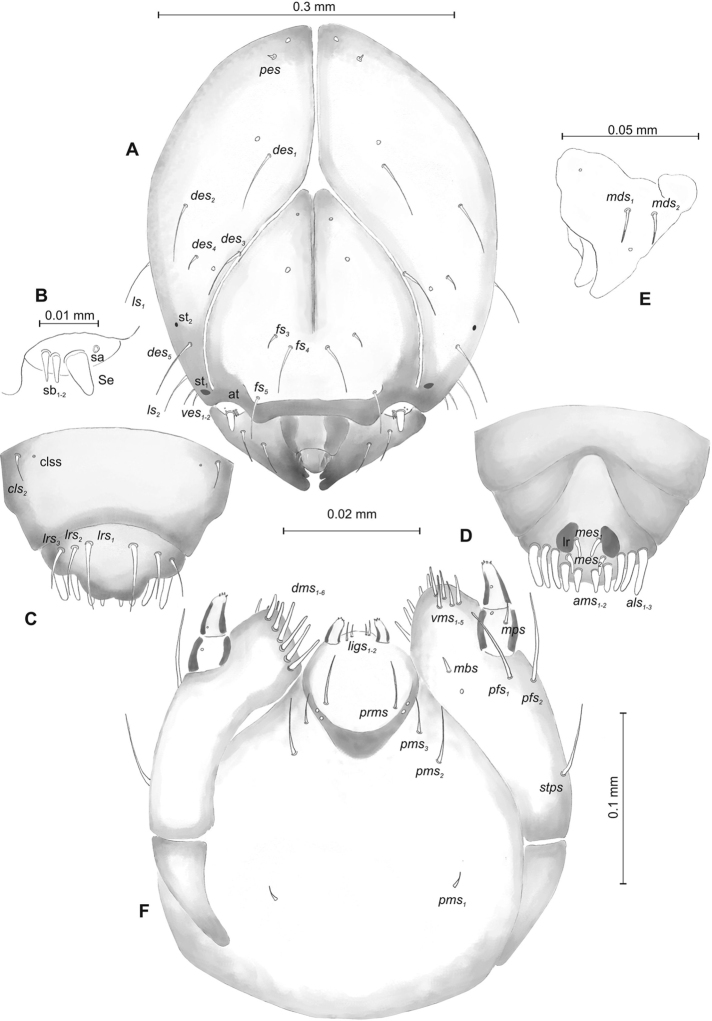
*Mecinus laeviceps* mature larva, head and mouth parts **A** head, frontal view **B** antenna **C** clypeus and labrum, dorsal view **D** epipharynx **E** left mandible **F** maxillolabial complex, ventral aspect. Abbreviations: *at* – antenna, *clss* – clypeal sensorium, *des* – dorsal epicranial, lr – labral rods, sa – sensillum ampullaceum, sb – sensillum basiconicum, Se – sensorium, st – stemmata, setae: *als* – anterolateral, *ams* – anteromedial, *cls* – clypeal, *dms* – dorsal malar, *fs* – frontal, *ligs* – ligular, *lrs* – labral, *ls* – lateral epicranial, *mbs* – malar basiventral, *mds* – mandibular, *mes* – median, *mxps* – maxillary palp, *pes* – postepicranial, *ves* – ventral, *pfs* – palpiferal, *pms* – postlabial, *prms* – prelabial, *stps* – stipal, *vms* – ventral malar.

(Figures 31A–D, 32A–F). ***Measurements*** (in mm). Body length: 1.67–2.67. Body width (abdominal segments I–II): 0.37–0.83. Head width: 0.30–0.40.

***Body*** (Figure 31A–D) yellowish, slender. Chaetotaxy rather weakly developed, setae capilliform, variable in length, light yellow. Prothorax (Figure 31B) with nine *prns* of unequal length (eight relatively long, one medium); two long *ps* and one medium *eus.* Meso- and metathorax (Figure 31B) with one short *prs*, two *pds*, different in length (medium, long), one short *as*, three short *ss*, one medium *eps*, one medium *ps* and one medium *eus.* Pedal area with three *pda*, different in length. Abdominal segments I–VIII (Figure 31C, D) with one short *prs*, three *pds* of different length (*pds_1,3_* short, *pds_2_* medium; all *pds* on segment VIII very long, equal in length), arranged along the posterior margin, two short *ss*, two short *eps*, one medium *ps*, one short *lsts* and two short *eus.* Abdominal segment IX (Figure 31D) with two long *ds*, located close to posterior margin, one long *ps* and two short *sts.* Each lateral anal lobe with one minute seta.

***Head capsule*** (Figures 31A, 32A–F) light yellow, distinctly narrowed bilaterally. *Des_1–3,5_* long, equal in length; *des_4_* very short; *des_4_* located in the central part of epicranium. *Fs_3_* short to very short, *fs_4,5_* long, equal in length. *Les_1_* and *les_2_* long, equal in length; two *ves* and one *pes* very short (Figure 32A). Antenna (Figure 32B) with sensorium (Se) conical, three times as long as wide, and three sensilla: one sa and two sb. Clypeus (Figure 32C) trapezium-shaped, anterior margin slightly concave; *cls_2_* medium; *clss* placed close to *cls_2_*. Labrum (Figure 32C) almost semi-circular, anterior margin sinuated; *lrs_1_* long, both *lrs_2_* and *lrs_3_* medium. Epipharynx (Figure 32D) with three finger-like *als* of almost equal length; two finger-like *ams*, both medium; two short finger-like *mes*, surface smooth; labral rods short, kidney-shaped. Mandibles (Figure 32E) conical, wide, with a small protuberance in the middle of the cutting edge; both *mds* capilliform, medium, equal in length, placed transversely. Maxilla (Figure 32F) with one *stps* and two *pfs* of equal length; *mbs* short; mala with six rod-like *dms* different in length (*dms_1,2_* medium, *dms_3–6_* long to very long), five *vms* different in length. Maxillary palpi: basal palpomere wider than distal, both of almost equal length. Prelabium (Figure 32F) oval-shaped with one long *prms*; ligula with two short *ligs*; premental sclerite clearly visible, U-shaped. Postlabium (Figure 32F) with very short *pms_1_*, long *pms_2_*, and medium *pms_3_*.

##### Description of pupa

**Figure 33.**
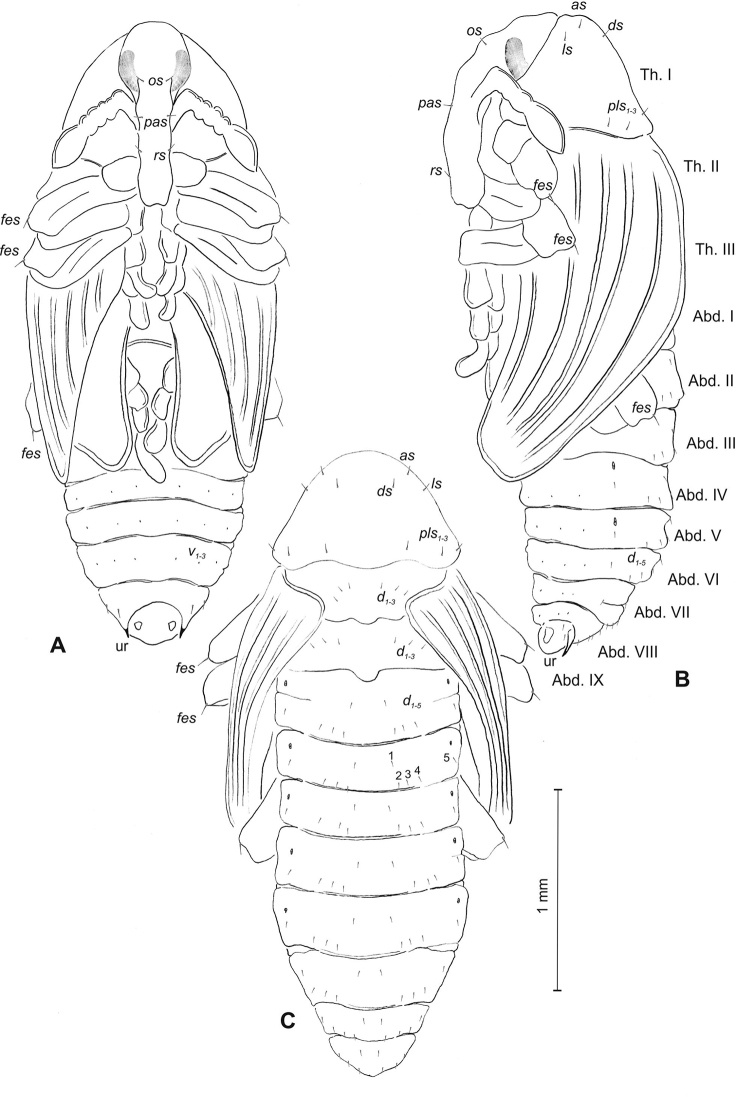
*Mecinus laeviceps* pupa habitus and chaetotaxy **A** ventral view **B** lateral view **C** dorsal view. Abbreviations: Th. I–III – number of thoracic segments, Abd. I–IX – number of abdominal segments, ur – urogomphi, setae: *as* – apical, *d* – dorsal, *ds* – discal, *fes* – femoral, *l*, *ls* – lateral, *os* – orbital, *sls* – postantennal, *pls* – posterolateral, *rs* – rostral.

(Figure 33A–C). ***Measurements*** (in mm). Head width: 0.35–0.40. Body width: 0.87–1.07. Body length: 2.12–2.50.

***Body*** elongated, white. Rostrum slender, about four times as long as wide, reaching up to mesocoxae. Antennae slender and elongated. Pronotum 1.5 times as wide as long. Urogomphi (ur) very short, conical, with sclerotised apex, only slightly reaching outline of the body, directed downward (Figure 33B, C).

***Chaetotaxy*** sparse, setae short, unequal length. Head with one *os*. Rostrum with one *rs* and one *pas*. Setae on head and rostrum straight, as long as those on prothorax (Figure 33A). Pronotum with one *as*, one *ls*, one *ds* and three *pls.* Dorsal parts of meso- and metathorax with three setae placed medially. Abdominal segments I–VIII with three very short setae ventrally, distributed in regular lines. Dorsal parts of abdominal segments I–VII with five setae (*d_1_* placed anteromedially, *d_2–4_* posteromedially, *d_5_* posterolaterally); segment VIII with four setae dorsally. Abdominal segment IX with two micro-setae ventrally.

##### Biological notes.

This is a monophagous species associated with broomleaf toadflax, *Linaria genistifolia* (L.) Mill. The adults are active from early spring, following the appearance of the growing shoots of its host plant. The adults feed intensively on shoot points and apical leaves. Females oviposit batches of 3–6 eggs into the lower to middle part of the young growing shoots. Larval development usually induces stunted growth in the young shoot. The larvae develop in the central part of the stem, forming a relatively short tunnel and the formation of a pseudo-gall in which pupation takes place in the larval chamber very close to the stem surface. Like *M. heydenii*, the adults stay inside the stem until mid-August, when all adults leave their host plants. Adults overwinter in the soil close to the host plant.

##### Remarks and comparative notes.

There are three cryptic subspecies of this species that are distinguishable by molecular and biogeographical data (Toševski et al. 2014). We studied the nominal subspecies distributed in the Czech Republic, Hungary, northern Serbia, and southern Russia; the subspecies meridionalis Toševski & Jović, 2014 is distributed in Serbia and Bulgaria and the subspecies corifoliae  Toševski & Jović, 2014 is distributed in Turkey. As reported in the remarks on *M. heydenii*, several characters allow us to distinguish this last species and *M. peterharrisi* from *M. laeviceps*.

#### Mecinus peterharrisi

Taxon classificationAnimaliaColeopteraCurculionidae

Toševski & Caldara, 2013

06998318-BC5D-5AE9-A4E1-FF13AE179A15

##### Material examined.

25 L3 larvae and 20 pupae, Mecedonia, Prilep, 25.07.2017, (41°17.354'N, 21°29.983'E, 618 m.) ex *Linaria dalmatica macedonica*, lgt. I. Toševski. Accession numbers of sequenced specimen MN992004.

##### Description of mature larva

**Figure 34.**
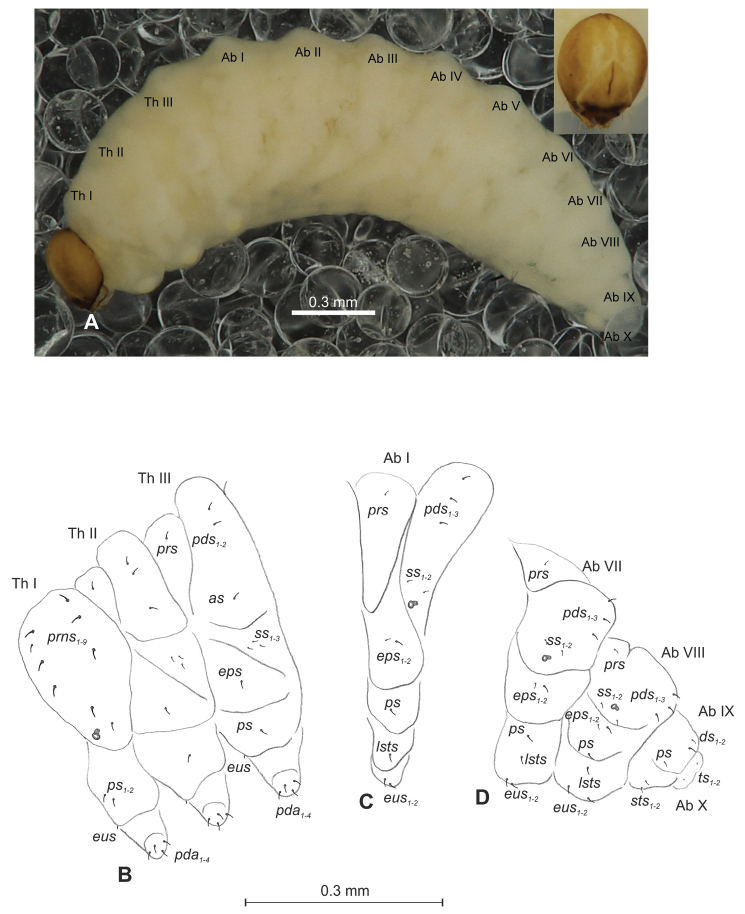
*Mecinus peterharrisi* mature larva, habitus and chaetotaxy **A** habitus of the body and frontal view of the head **B** lateral view of thoracic segments **C** lateral view of abdominal segment I **D** lateral view of abdominal segments VII–X. Abbreviations: Th. I–III – number of thoracic segments, Abd. I–X – number of abominal segments, setae: *as* – alar, *ds* – dorsal, *eps* – epipleural, *eus* – eusternal, *lsts* – laterosternal, *pda* – pedal, *pds* – postdorsal, *prns* – pronotal, *prs* – prodorsal, *ps* – pleural, *ss* – spiracular, *sts* – sternal, *ts* – terminal.

**Figure 35.**
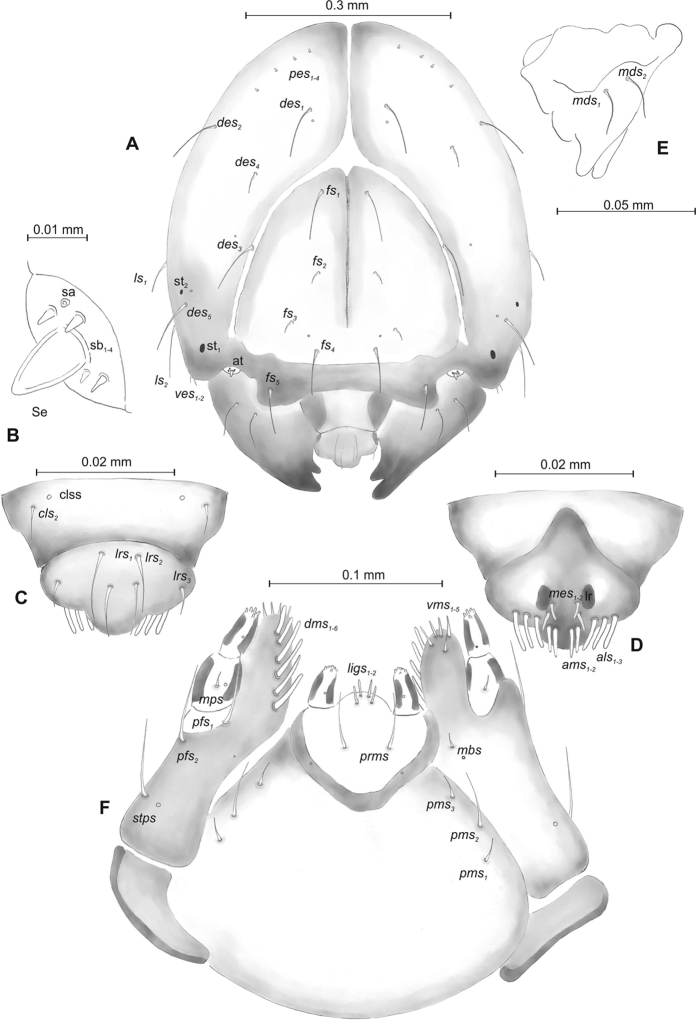
*Mecinus peterharrisi* mature larva, head and mouth parts **A** head, frontal view **B** antenna **C** clypeus and labrum, dorsal view **D** epipharynx **E** left mandible **F** maxillolabial complex, ventral aspect. Abbreviations: *at* – antenna, *clss* – clypeal sensorium, *des* – dorsal epicranial, lr – labral rods, sa – sensillum ampullaceum, sb – sensillum basiconicum, Se – sensorium, st – stemmata, setae: *als* – anterolateral, *ams* – anteromedial, *cls* – clypeal, *dms* – dorsal malar, *fs* – frontal, *ligs* – ligular, *lrs* – labral, *ls* – lateral epicranial, *mbs* – malar basiventral, *mds* – mandibular, *mes* – median, *mxps* – maxillary palp, *pes* – postepicranial, *ves* – ventral, *pfs* – palpiferal, *pms* – postlabial, *prms* – prelabial, *stps* – stipal, *vms* – ventral malar.

(Figures 34A–D, 35A–F). ***Measurements*** (in mm). Body length: 2.00–3.75. Body width (metathorax and abdominal segments I–II): 0.65–1.00. Head width: 0.35–0.43.

***Body*** (Figure 34A–D) yellowish, very slender. Chaetotaxy weakly developed, setae extremely short, difficult to observe. Prothorax (Figure 34B) with nine short *prns*; two short *ps* and one short *eus.* Meso- and metathorax (Figure 34B) with one short *prs*, two short *pds*, one short *as*, three minute *ss*, one short *eps*, one short *ps* and one short *eus.* Pedal area with four short *pda.* Abdominal segments I–VIII (Figure 34C, D) with one short *prs*, three short *pds* arranged along the posterior margin, two minute *ss*, two short *eps*, one short *ps*, one short *lsts* and two short *eus.* Abdominal segment IX (Figure 34D) with two *ds* located close to the posterior margin, one short *ps* and two short *sts.* Each of anal lobe with two minute setae.

***Head capsule*** (Figures 34A, 35A–C) pale yellow, distinctly narrowed bilaterally. Frontal suture well visible. *Des_1–3,5_* very long, equal in length; *des_4_* three times shorter than other *des. Fs_1_* as long as *des_1_*, *fs_2_* and *fs_3_* very short, *fs_4–5_* long. *Les_1_* shorter than *les_2_*; two *ves* and four *pes* very short (Figure 35A). Two stemmata of different size. Antennae (Figure 35B) with sensorium (Se) conical, thrice as long as wide, located medially, and three sensilla of different types: one sa and four sb. Clypeus (Figure 35C) trapezium-shaped, anterior margin slightly concave; *cls_2_* medium; *clss* clearly visible. Labrum (Figure 35C) with sinuate anterior margin; *lrs_1_* very long, *lrs_2_* shorter than *lrs_1_*, *lrs_3_* three times shorter than *lrs_1_*. Epipharynx (Figure 35D) with three relatively long, finger-shaped *als* of almost equal length; two rod-like *ams*, equal in length; two rod-like *mes* of medium length; surface smooth; labral rods short, rounded. Mandibles (Figure 35E) conical, wide, with a small protuberance in the middle of the cutting edge; both *mds* capilliform, relatively long, equal in length, placed mediolaterally. Maxilla (Figure 35F) with one *stps* and two *pfs* of equal length; *mbs* very short; mala with six finger-like *dms* different in length (*dms_1,2_* medium, *dms_3–6_* long to very long), five *vms* different in length. Maxillary palpi: basal palpomere wider than distal, both of almost equal length. Prelabium (Figure 35F) close to oval-shaped with one very long *prms*; ligula with two *ligs* of equal length; premental sclerite clearly visible, U-shaped. Postlabium (Figure 35F) with short *pms_1_*, long *pms_2_*, and short *pms_3_*.

##### Description of pupa

**Figure 36.**
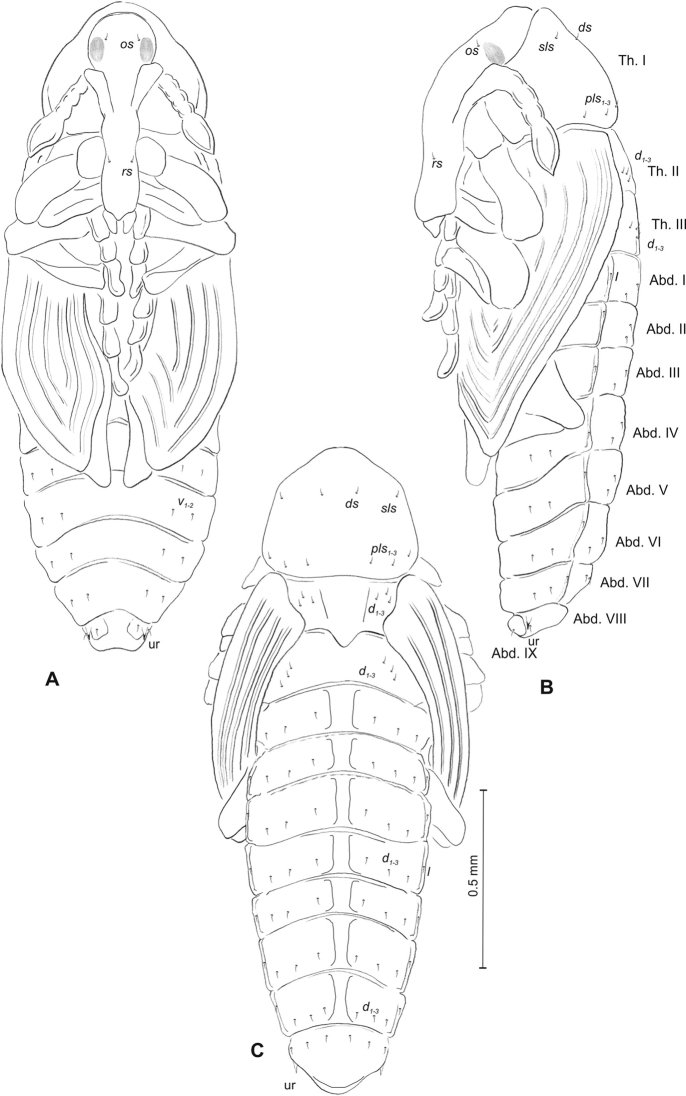
*Mecinus peterharrisi* pupa habitus and chaetotaxy **A** ventral view **B** lateral view **C** dorsal view. Abbreviations: Th. I–III – number of thoracic segments, Abd. I–IX – number of abdominal segments, ur – urogomphi, setae: *as* – apical, *d* – dorsal, *ds* – discal, *fes* – femoral, *l* – lateral, *os* – orbital, *pls* – posterolateral, *rs* – rostral, *sls* – superlateral.

(Figure 36A–C). ***Measurements*** (in mm). Head width: 0.36–0.43. Body width: 0.83–1.50. Body length: 2.46–3.66.

***Body*** elongated, slender, white. Rostrum slender, about five times as long as wide, reaching up to mesocoxae. Antennae slender and moderately elongated. Pronotum 1.1 times as wide as long. Urogomphi (ur) very short, conical, with sclerotised apex, reaching outline of the body, directed downward (Figure 36A, B).

***Chaetotaxy*** sparse, setae very short, equal in length. Head with one *os*. Rostrum with one *rs* placed medially. Setae on head and rostrum straight, as long as those on prothorax (Figure 36A). Pronotum with one *ds*, one *sls* and three *pls.* Dorsal parts of meso- and metathorax with three setae placed medially. Abdominal segments I–VIII with one seta laterally, two very short setae ventrally and three setae dorsally, placed along posterior margin. Abdominal segment IX with two micro-setae ventrally. (Figure 36B).

##### Biological notes.

This species is known only from the southwest region of North Macedonia and northwestern part of Greece following the distribution of *Linaria dalmatica macedonica* (Griseb.) D.A. Sutton, as well as from sparse populations of *L. dalmatica dalmatica* (L.) Mill. present at the Montenegrin Sea coast in the vicinity of Kotor Bay (Toševski et al. 2014). Adults appear in the field at the beginning of March and aggregate on young growing shoots, causing significant damage to the shoot points and apical leaves. Like *M. laeviceps*, the larvae develop within short larval tunnels, and the newly emerged adults remain and feed in the pupal chamber until mid-August. Adults overwinter in the soil close to the host plant.

##### Remarks and comparative notes.

This species, found only in the Balkans (Macedonia, Greece, Montenegro) as above reported, is very similar to *M. laeviceps*, from which it differs by the body being more robust and usually longer, the integument of the pronotum more distinctly bluish, and the penis with longer tip. Moreover, the vestiture is usually formed by slightly broader scales and is therefore generally more distinct. As reported in the remarks on *M. heydenii* and in the keys, the study of the immatures has revealed other interesting differences between these three species that are very useful for their separation.

### Key to known mature larvae of *Mecinus* species

The following key is based on the larvae, described in this paper, of 12 *Mecinus* species.

**Table:** 

1	Head rounded or almost rounded, with only a single pair of stemmata. Abdominal segment X reduced to three anal lobes of equal size	**2**
–	Head distinctly flattened laterally, with two pairs of stemmata. Abdominal segment X reduced to three anal lobes of those lateral are the largest, and dorsal the smallest (sometimes absent)	**5**
2	Head brown, only slightly narrowed bilaterally. Endocarina reaching 1/2 of the frons. Body slightly pressed dorso-ventrally. Premental sclerite, pedal lobes and spiracular area of meso- and metathorax dark pigmented. Thoracic spiracle bicameral. *Cls_1_* present. Labial palpi two-segmented	***Mecinus collaris* Germar, 1821**
–	Head white, rounded. Endocarina reaching 3/4 of the frons. Body rounded in cross section. Premental sclerite, pedal lobes and spiracular area of meso- and metathorax as pigmented as rest of the body. Thoracic spiracle unicameral. *Cls_1_* absent. Labial palpi one-segmented	**3**
3	Cuticle tuberculate. *Fs_1_* absent. Thoracic segments with two *pds.* Abdominal segments I–VIII with three *pds.* Anterior margin of labrum almost straight. Posterior extension of premental sclerite elongated, with acute apex	***Mecinus pirazzolii* (Stierlin, 1867)**
–	Cuticle smooth. *Fs_1_* present. Thoracic segments with three *pds.* Abdominal segments I–VIII with four *pds.* Anterior margin of labrum sinuated. Posterior extension of premental sclerite short, with dull apex	**4 (*Mecinus pascuorum* group)**
4	Pronotum with 11 *prns*. Thoracic *pds* various in length (first short, second and third elongated). Thoracic *ss* various in length (first minute, second and third medium). Each pedal lobes with four *pda.* Setae on abdominal segment VIII relatively elongated. *Prms* very short. Antenna with two sb and one sa	***Mecinus labilis* (Herbst, 1795)**
–	Pronotum with eight *prns*. Thoracic *pds* equal in length. Thoracic *ss* equal in length. Each pedal lobes with five *pda.* Setae on abdominal segment VIII medium size. *Prms* elongated. Antenna with three sb	***Mecinus pascuorum* (Gyllenhal, 1813)**
5	Abdominal segments I–VIII with four *pds* and usually three *ss.* Labial palpi two-segmented. Surface of postlabium (at least partially) densely covered with asperities	**6** (***Mecinus janthinus* group)**
–	Abdominal segments I–VIII with three *pds* and always two *ss.* Labial palpi one-segmented. Surface of postlabium smooth	**8**
6	Abdominal segments I–VIII with two *ss.* Only posterior part of postlabium covered with asperities	***Mecinus sicardi* Hustache, 1920**
–	Abdominal segments I–VIII with three *ss.* Whole postlabium covered with asperities	**7**
7	Pronotum with eight *prns*. *Ss* on thoracic segments various in length (two minute, one medium). Each pedal lobes with five *pda.* Head with one *ves* and four *pes.* Antenna with two sb and one sa. Mala with six *dms.* Ligula with three *ligs*	***Mecinus janthinus* Germar, 1821**
–	Pronotum with 11 *prns*. *Ss* on thoracic segments medium, equal in length. Each pedal lobes with six *pda.* Head with two *ves* and three *pes.* Antenna with four sb. Mala with seven *dms.* Ligula with two *ligs*	***Mecinus janthiniformis* Toševski & Caldara, 2011**
8	Thoracic spiracle bicameral. Endocarina reaching to 1/2 of the frons. *Cls_1_* present. Premental sclerite cup-like. All *dms* equal in length	**9** (***Mecinus circulatus* group)**
–	Thoracic spiracle unicameral. Endocarina reaching to 4/5 of the frons. *Cls_1_* absent. Premental sclerite U-shaped. *Dms_1_*,*_2_* always shorter than next one	**10** (***Mecinus heydenii* group)**
9	Pronotum with eight *prns*. Each pedal lobes with three *pda.* Anal lobes with one *ts.* Head with five *pes.* Mandible with two *mds.* Mala with four *vms. Prms* short	***Mecinus circulatus* (Marsham, 1802)**
–	Pronotum with 11 *prns*. Each pedal lobes with five *pda.* Anal lobes with two *ts.* Head with four *pes.* Mandible with one *mds.* Mala with five *vms. Prms* elongated	***Mecinus pyraster* (Herbst, 1795)**
10	*Fs_1_* long, *fs_2_* minute. Antenna with four sb	***Mecinus peterharrisi* Toševski & Caldara, 2013**
–	*Fs_1_* and *fs_2_* absent. Antenna with two sb	**11**
11	Pronotum with eight *prns*. Thoracic segments with three *pds.* Each pedal lobes with four *pda. Pds* of abdominal segments I–VIII minute. Head with four *pes*	***Mecinus heydenii* Wencker, 1866**
–	Pronotum with nine *prns*. Thoracic segments with two *pds.* Each pedal lobes with three *pda. Pds* of abdominal segments I–VIII medium or elongated. Head with one *pes*	***Mecinus laeviceps* Tournier, 1873**

### Key to pupae of known *Mecinus* species

The following key is based on pupae of 12 *Mecinus* species described in this paper.

**Table:** 

1	Body stout, length ratio at most 1.8	**2**
–	Body slender, length ratio at least 2.0	**4**
2	Rostrum slender, very short, distinctly tapering to its top. Setae on head and pronotum extremely short, almost invisible. Abdominal segments I–VII without setae ventrally	***Mecinus pirazzolii* (Stierlin, 1867)**
–	Rostrum moderately elongated, linear, 2–2.5 times as long as wide. Setae on head and proonotum minute or medium. Abdominal segments I–VII with three setae ventrally	**3**
3	Setae of head and pronotum medium, well visible. Pronotum with two protuberances apically	***Mecinus pascuorum* (Gyllenhal, 1813)**
–	Setae of head and pronotum minute, weakly visible. Pronotum without protuberances apically	***Mecinus labilis* (Herbst, 1795)**
4	Abdominal segments I–IV dorsally without setae; segments V–VII with setae growing gradually	***Mecinus collaris* Germar, 1821**
–	Abdominal segments I–VII dorsally with setae equal in length or only slightly growing from segment I to VII	**5**
5	Urogomphi short or very short, only slightly reaching outline of the body, directed downward. Setae of head and pronotum medium or short. Abdominal segments I–VII with five or less setae dorsally	**6**
–	Urogomphi relatively elongated, distinctly reaching outline of the body, directed outside. Setae of head and pronotum elongated. Abdominal segments I–VII with six or seven setae dorsally	**10 (*Mecinus janthinus* group)**
6	Body moderately slender. Head with one *vs*, one or two *sos* and one *sls*; Pronotum with two *sls* and one or two *ls.* Dorsal parts of meso- and metathorax with two setae dorsally	**7** (***Mecinus circulatus* group**)
–	Body very slender. Head without *vs* and *sos*, and *sls* also usually absent. Pronotum without or with one *sls* and *ls.* Dorsal parts of meso- and metathorax with three setae dorsally	**8 (*Mecinus heydenii* group)**
7	Head with two *sos* and two *os*. Pronotum with two *as*, one *ds*, two *ls*, and three *pls.* Abdominal segments I–VII with five setae dorsally	***Mecinus pyraster* (Herbst, 1795)**
–	Head with one *sos* and one *os*. Pronotum with one *as*, *ds* absent, one *ls*, and two *pls.* Abdominal segments I–VII with three setae dorsally	***Mecinus circulatus* (Marsham, 1802)**
8	Pronotum without *as.* Abdominal segments I–VII with three setae dorsally. Femora without *fes*	***Mecinus peterharrisi* Toševski & Caldara, 2013**
–	Pronotum with some *as.* Abdominal segments I–VII with five setae dorsally. Femora with one *fes*	**9**
9	Rostrum with one *pas*. Pronotum with one *ls*	***Mecinus laeviceps* Tournier, 1873**
–	Rostrum without *pas*. Pronotum without *ls*	***Mecinus heydenii* Wencker, 1866**
10	Rostrum with one *sls* and without *rs.* Pronotum with four *pls*	***Mecinus sicardi* Hustache, 1920**
–	Rostrum with two *sls* and one *rs.* Pronotum with three *pls*	**11**
11	Dorsal parts of meso- and metathorax with three setae dorsally	***Mecinus janthiniformis* Toševski & Caldara, 2011**
–	Dorsal parts of meso- and metathorax with two setae dorsally	***Mecinus janthinus* Germar, 1821**

## Discussion

### Comparison with immature stages of known Mecinini

The present detailed descriptions of the immature stages of 12 species of *Mecinus* constitute a good sample, comprising approximately 20% of the known species of this genus, allowing a comparison with other genera within the tribe Mecinini. Unfortunately, some descriptions previously published on immature stages of species belonging to other genera of Mecinini are somewhat problematic because of missing details about the chaetotaxy and/or the absence of quality drawings (see Skuhrovec et al. 2018), making such comparisons still difficult. Only the recent descriptions of six *Cleopomiarus* and three *Miarus* species (Skuhrovec et al. 2018; Szwaj et al. 2018), three *Gymnetron* species (Jiang and Zhang 2015), and two *Rhinusa* species (Gosik 2010; Ścibior and Łętowski 2018) were sufficiently complete for such a comparison. Skuhrovec et al. (2018) emphasised that the taxonomical interpretation of some characters (e.g., thoracic and abdominal dorsal setae) in the papers above is very disputable. This can cause an incorrect differential diagnosis and preclude the composition of a key to the tribe. Our new data might be able to resolve some of these uncertainties.

The number of palpomeres of the labial palpi was shown to be one of the most important morphological characters of larvae in this tribe (Skuhrovec et al. 2018). Some *Gymnetron* species have only one palpomere (May 1993; Jiang and Zhang 2015), but the basal state in weevils is the presence of two palpomeres on labial palpi (Marvaldi 1997). *Mecinus* species can be clearly separated into two groups based on the presence of both states. Some species groups have the plesiomorphic condition, with two palpomeres on the labial palpi, but other species groups have only one palpomere, such as *Gymnetron* species. A completely different situation has been observed in some *Cleopomiarus* species (Skuhrovec et al. 2018), where there is not a distinct separation of the basal palpomere from the labium, and it can appear to be only one palpomere. This state in *Cleopomiarus* and partially in *Miarus* could be an intermediate stage in the reduction to *Gymnetron* species. This should also be compared with a higher number of *Rhinusa* species, and only then the evolutionary history of this character in the whole tribe can be discussed.

Skuhrovec et al. (2018) suggested that the number of air tubes of the thoracic and abdominal spiracles is the next crucial genus-specific character in Mecinini larvae. Larvae of *Mecinus* species have two states of this character: (1) all spiracles unicameral, as in *Gymnetron* species (Jiang and Zhang 2015), or (2) thoracic spiracle bicameral and abdominal spiracles unicameral, as in some *Rhinusa* (Anderson 1973; May 1993). A completely unique situation is seen in all known larvae of *Cleopomiarus* and *Miarus* species, which have bicameral spiracles on the thorax and abdomen.

Another disputable state is the number of epipharyngeal setae (especially *ams* and *mes*), which is not yet completely resolved in Curculionidae and was also discussed several times for different groups, e.g., Lixinae (Gosik and Skuhrovec 2011; Stejskal et al. 2014; Trnka et al. 2015). In our view, the final decision of the number of each seta is important, but not crucial, and the comparison between groups/genera should be made together for all three of these epipharyngeal setae in order to make fewer mistakes in the creation of a differential diagnosis for genera in the tribe.

The last important characteristic observed within the Mecinini tribe is the integument of the body with distinct asperities (Skuhrovec et al. 2018). This feature is very variable within each genus (*Mecinus*, *Cleopomiarus*, *Miarus*), probably due to specific environmental conditions within plant tissues. This feature may be discussed after other detailed descriptions are made within the Mecinini tribe.

### Comparison of the immature stages of *Mecinus* species groups

Before this study, larvae of only four *Mecinus* species had been described – *M. pascuorum*, *M. pyraster*, *M. heydenii*, and *M. janthinus* (Emden 1938; Scherf 1964; Anderson 1973), while a description of pupae was available for only three of these species (Scherf 1964; Anderson 1973). Unfortunately, these descriptions did not include the chaetotaxy, with a few exceptions, and included only general characteristics, such as the number of teeth on the mandible or the colouration of the head and body. Therefore, a detailed description of all four species has been necessary for their incorporation into our key.

The main differential characters in larvae and pupae among known species are presented in the diagnosis of species groups and in the keys. The 12 species described here belong to five groups and one complex of the seven groups and two complexes detected by Caldara et al. (2013) on the basis of morphological and biological apomorphies. It should be recalled that these authors defined their complexes as an assemblage of species formed by several taxa, which are mostly very similar to each other but lacking synapomorphies in contrast with the species forming the groups. Therefore, we now have the opportunity to assess whether the differences found in the immatures are able to confirm or refute the conclusions obtained after a phylogenetic study of the imagoes.

Based on several unique characters, we can confirm that all six assemblages of species obtained in the present study completely agree with those based on adult characters. Moreover, we found distinctive characters in the previously considered “complex” of *M. pascuorum*, which can now be considered a “true” group.

The main differential characters in larvae among the known species include the following: (1) the number of palpomeres of the labial palpi, (2) the number of air tubes of the thoracic and abdominal spiracles, and (3) the shape of the head and the number of stemmata on the head. The combination of the states of these three characters can easily separate all species groups. Only future studies of the whole tribe together with adult morphology and biological information may identify the values of each character and verify its effect on evolution within this tribe. Skuhrovec et al. (2018) reported that fewer genus-specific character states in larvae than in pupae, which are more conservative in chaetotaxy, were also shown in another tribe of the Curculioninae (Tychiini) with regard to the genera *Tychius* Germar, 1817 and *Sibinia* Germar, 1817 (see Skuhrovec et al. 2014, 2015; Gosik et al. 2017).

According to the two above-mentioned main characters of larvae, the groups of *Mecinus* could be assembled as follows: (1) two palpomeres on the labial palpi: *M. collaris*, *M. janthinus* groups; (2) one palpomere: *M. heydenii*, *M. pascuorum*, *M. circulatus*, *M. simus* groups; (3) all spiracles unicameral: *M. heydenii*, *M. pascuorum*, *M. simus* group; (4) thoracic spiracle bicameral and abdominal spiracles unicameral: *M. collaris*, *M. janthinus*, *M. circulatus* groups. However, if the two characters are combined, we obtain the following groupings: (1) two palpomeres on the labial palpi + thoracic spiracle bicameral and abdominal spiracles unicameral: *M. collaris* and *M. janthinus* groups; (2) one palpomere + all spiracles unicameral: *M. simus*, *M. heydenii* and *M. pascuorum* groups; (3) one palpomere + thoracic spiracle bicameral and abdominal spiracles unicameral: *M. circulatus* group.

Species of the genus *Mecinus* feed on different genera of host plants in two different tribes belonging to the family Plantaginaceae: *Plantago* (Plantagineae) and *Linaria*, *Antirrhinum* and *Anarrhinum* (Antirrhineae). The *M. heydenii* group and the *M. janthinus* group include all the species of *Mecinus* living on Antirrhineae. Until now, this ecological character was the unique putative synapomorphy that allows the assemblage of these two groups, although these species are clearly similar overall, and only these groups include species with blue elytral integument. This character is not possessed by any other Mecinini, and the other species of these groups have black elytral integument. In contrast, most species living on *Plantago*, at least in part, have reddish integument. Caldara et al. (2013) did not find other consistent synapomorphies that allow the separation of the *M. janthinus* + *M. heydenii* groups feeding on Antirrhineae from all other species living on *Plantago*. Similarly, a molecular study did not relate these two groups which possibly evolved in parallel (I. Toševski, unpublished data).

Unfortunately, the morphology of the immatures does not seem to shed more light on this situation. In fact, the two groups living on Antirrhineae share only the following two characters in larvae: (1) head brown, distinctly narrowed bilaterally, with two pairs of stemmata and (2) endocarina reaching 4/5 of the frons. The characters of the head are also possessed by the *M. circulatus* group, which, in contrast, has the endocarina reaching only half of the frons. With regard to the pupae, the relationship of these three groups might be suggested by abdominal segments I–VII dorsally with setae. However, if the available data are assembled, one could assume that the *M. janthinus* + *M. heydenii* groups are not monophyletic sharing only a few homoplasies and suggesting that the switch from Plantaginaceae s. str. to Antirrhineae occurred independently in both the *M. heydenii* and *M. janthinus* species groups. It is noteworthy that the species of *Mecinus* that share an unusual elongated body, i.e., *M. circulatus*, *M. janthinus* and *M. heydenii*, are generally stem borers, with larval feeding and mining in the central part of the stem producing no externally visible damage or small external gall-like deformations – except for *M. dorsalis* Aubé, 1850 of the *M. heydenii* group, which produces globose galls – suggesting that the elongated body is an adaptative character for their ecological niche. At present, the only known gall-inducing species living on *Plantago* is *M. collaris*, which appears morphologically distinct from all these species.

### Differences between immatures at the species level

All the studied immatures have characters that allow us to distinguish them from each other. Whereas it was expected that very characteristic species such as *M. collaris* or *M. labilis* belong to different groups on the basis of the morphology of the adult, it is also true for species within the same group. Moreover, it is even more noteworthy that cryptic species, such as *M. janthinus* and *M. janthiniformis* and *M. heydenii*, *M. peterharrisi* and *M. laeviceps*, are clearly distinguishable by the morphological characters of the immatures. Differences were found in the lack or presence and, in the latter case, the number of setae both in larvae (head, pronotum, thoracic and abdominal segments) and pupae (rostrum, pronotum, abdomen).

### Biological and evolutionary considerations

It appears probable that all *Mecinus* species live on Plantaginaceae. The majority of them feed on species of *Plantago*, whereas a quarter of *Mecinus* species live on Antirrhineae, especially *Linaria* and occasionally *Antirrhinum* and *Anarrhinum*. As reported above, neither the study on adults by Caldara et al. (2013) nor ours on the immature stages have found consistent synapomorphies that allow the separation of the *M. janthinus* + *M. heydenii* groups feeding on Antirrhineae from all other species living on *Plantago*. It is noteworthy that no mecinines other than *Mecinus* live on *Plantago* (Caldara et al. 2013), whereas several species belonging to *Rhinusa*, the sister-group of *Mecinus*, live on Antirrhineae (Caldara et al. 2010). These data as a whole tend to suggest that Mecinini, during their evolution, switched more than once from Plantagineae to Antirrhineae or vice versa, and that this switch could easily have occurred independently in both the *M. heydenii* and *M. janthinus* species groups.

The larvae of closely related species of *Mecinus* seem to differ in their modes of parasitism, although less significantly than *Rhinusa* larvae (see Caldara et al. 2010). Concerning the species living on *Plantago*, the apparently more primitive species belonging to the *M. pascuorum* group feed on pyxidia without producing externally visible damage. In contrast, *Mecinus circulatus* and *M. pyraster* of the *M. circulatus* group are stem borers with larval feeding and mining in the central part of the stem, producing no externally visible damage or small external gall-like deformations. At present, the only known gall-inducing species living on *Plantago* is *M. collaris*, which appears morphologically distinct from all other species. The situation of the *M. simus* group is very interesting: whereas *M. pirazzolii* feed on seeds, *M. comosus* Boheman, 1845 tunnels into the central axis of the spike of *Plantago maritima* (Prena and Caldara 2017). With regard to the species living on Antirrhineae, the same variability in biological habits is found in the *M. heydenii* group: from *M. heydenii* producing weak deformation of the stems to *M. dorsalis* producing globose galls. In contrast, in the *M. janthinus* group, there are true stem borers that produce no visible damage (*M. janthinus* and *M. barbarus*) or only small gall-like deformations (*M. janthiniformis*, *M. sicardi*). One thing seems clear: all the larvae of *Mecinus* species with elongated bodies develop along stems or, as in *M. comosus*, along spikes. It is worth noting that the genus *Mecinus* was created for these elongated species and that the other species with short bodies were described and considered *Gymnetron* for many years, until Caldara (2001) transferred them from *Gymnetron* to *Mecinus* on the basis of several synapomorphies in the male genitalia. It is also noteworthy that it was very simple to identify the species of *Mecinus* as they were formerly defined, since there are very few Curculioninae with similar elongate bodies. If we now consider the very probable polyphyly of species with long bodies on the basis of morphological, biological and molecular characters, we can speculate that the elongated body is an adaptive character for the particular ecological niche, i.e., the somewhat narrow stems of *Plantago* and *Linaria* or the spikes of *Plantago*. It is noteworthy that stems are not used as niches for the growth of larvae of other Curculioninae, except for *Rhinusa asellus* (Gravenhorst, 1807) and *R. tenuirostris* (Stierlin, 1888) (Doğanlar and Üremiş 2014), which are the more elongated species in the *R. tetra* group that usually feed on the seeds of *Verbascum* spp.

## Conclusions

Our detailed descriptions of the immature stages of the Mecinini species demonstrate their importance for the taxonomy and further study of the phylogenetic relationships within the genera of the tribe Mecinini, although the number of described immatures is still low in comparison with the total number of *Mecinus* species. This is our second paper about the Mecinini, after that of *Miarus* and *Cleopomiarus*. We are confident that the description of immatures of the genera *Rhinusa* and *Gymnetron*, which are currently under investigation, will provide an interesting final arrangement to the taxonomy of the tribe.

## Supplementary Material

XML Treatment for Mecinus

XML Treatment for Mecinus pascuorum

XML Treatment for Mecinus pascuorum

XML Treatment for Mecinus labilis

XML Treatment for Mecinus simus

XML Treatment for Mecinus pirazzolii

XML Treatment for Mecinus circulatus

XML Treatment for Mecinus circulatus

XML Treatment for Mecinus pyraster

XML Treatment for Mecinus collaris

XML Treatment for Mecinus collaris

XML Treatment for Mecinus janthinus

XML Treatment for Mecinus janthinus

XML Treatment for Mecinus janthiniformis

XML Treatment for Mecinus sicardi

XML Treatment for Mecinus heydenii

XML Treatment for Mecinus heydenii

XML Treatment for Mecinus laeviceps

XML Treatment for Mecinus peterharrisi
